# Tests for central sensitization in general practice: a Delphi study

**DOI:** 10.1186/s12875-021-01539-0

**Published:** 2021-10-19

**Authors:** Carine den Boer, Berend Terluin, Johannes C. van der Wouden, Annette H. Blankenstein, Henriëtte E. van der Horst

**Affiliations:** Department of General Practice, Amsterdam Public Health Research Institute, Amsterdam UMC, location VUmc, Van der Boechorststraat 7, 1081 BT Amsterdam, The Netherlands

**Keywords:** Tests, Central sensitization, Medically unexplained symptoms, Persistent physical symptoms, Chronic pain

## Abstract

**Introduction:**

Central sensitization (CS) may explain the persistence of symptoms in patients with chronic pain and persistent physical symptoms (PPS). There is a need for assessing CS in the consultation room. In a recently published systematic review, we made an inventory of tests for CS. In this study we aimed to assess which tests might have added value, might be feasible and thus be suitable for use in general practice.

**Methods:**

We conducted a Delphi study consisting of two e-mail rounds to reach consensus among experts in chronic pain and PPS. We invited 40 national and international experts on chronic pain and PPS, 27 agreed to participate. We selected 12 tests from our systematic review and additional searches; panellists added three more tests in the first round. We asked the panellists, both clinicians and researchers, to rate these 15 tests on technical feasibility for use in general practice, added value and to provide an overall judgement for suitability in general practice.

**Results:**

In two rounds the panellists reached consensus on 14 of the 15 tests: three were included, eleven excluded. Included were the Central Sensitization Inventory (CSI), pressure pain thresholds (PPTs) and monofilaments. No consensus was reached on the Sensory Hypersensitivity Scale.

**Conclusion:**

In a Delphi study among an international panel of experts, three tests for measuring CS were considered to be suitable for use in general practice: the Central Sensitization Inventory (CSI), pressure pain thresholds (PPTs) and monofilaments.

**Supplementary Information:**

The online version contains supplementary material available at 10.1186/s12875-021-01539-0.

## Introduction

Central sensitization (CS) may explain the persistence of symptoms in the absence of specific somatic or psychiatric disease, which are very prevalent in healthcare [1–3].

The International Association for the Study of Pain (IASP) defined CS in 2011 as: “an increased responsiveness of nociceptive neurons in the central nervous system to their normal or sub-threshold afferent input” [4]. CS has been studied in relation to chronic pain and persistent physical symptoms (PPS). PPS is replacing the frequently used term medically unexplained (physical) symptoms, covering a large number of symptoms for which no explanation is found [1, 5–7]. We explained aspects of CS in Table 1.

**Table 1:** Explanation of aspects of CS

**Dorsal horn of the spinal cord**: in case of CS, sensory signals from the body can be increased in the dorsal horn, due to structural and functional changes, as an increase of receptors and neurotransmitters [8].	
**Ascending and descending pathways:** sensory signals from the body go through afferent nerves to the dorsal horn of the spinal cord and further through ascending pathways to the brain. These signals are processed in the brain and are sent back to the body through the descendent pathways. The brain can increase the signals in case of danger, and inhibit them in case of no alarm. In case of CS there is reduced inhibition.	
**Hyperalgesia**: increased sensitivity to painful stimuli	
**Allodynia**: painful perception of non-painful stimuli	
**Temporal summation (TS)**: TS refers to the phenomenon of increased pain perception in response to repetitive noxious stimuli over time. In case of CS the amplification of stimuli in the ascending neuronal pathways leads to an increase of TS [9–12].	
**Conditioned pain modulation (CPM):** CPM refers to the phenomenon that ‘pain inhibits pain’, the reduction in experienced pain for a tested stimulus due to the interference of a second stimulus (conditioning stimulus) applied at the same time but to a remote body location. In case of CS CPM will show a smaller reduction in pain sensitivity due to hyperexcitability of the central nervous system and reduction of descending inhibition [13–15].	

For patients with chronic pain and PPS, the explanation of the mechanism of CS might lead to a better understanding of how symptoms persist [16, 17]. They often struggle to accept that there is no conclusive medical explanation for their symptoms and feel misunderstood in their search for a medical diagnosis [18]. Diagnostic procedures often take a long time and may involve many different medical specialists. This can cause delays in an appropriate treatment of the symptoms, contributing to a deterioration of the symptoms [19]. Addressing perpetuating factors, like unhelpful cognitions, emotions, behaviour and social factors, are the most important goal in the treatment of chronic pain and PPS [20, 21]. The longer these perpetuating factors exist, the more difficult they are to change due to loss of physical fitness, work and social contacts [22]. Finally, doctors often feel frustrated, costs for medical care, as well as the societal costs for sickness leave, can be high [23].

But how do the doctor and the patient know that CS is an appropriate explanation for the persistence of the symptoms? After all, there is no gold standard for CS. We collected information in our systematic review on available tests for measuring CS [1, 13]. These tests include various forms of quantitative sensory testing (QST) and two combined QST tests to measure conditioned pain modulation (CPM) [13–15, 24]. QST tests are performed with various stimuli: mechanical stimuli, cold, heat, electricity, ischemia and vibrations [10, 11, 25].

Furthermore, in CS dysregulation of the immune system and an increase of neurotrophins play a role [1]. Cytokines as interleukins and TNF-alpha, and neurotrophins as brain derived neurotrophic factor (BDNF) can be measured in blood samples [26–30]. Structural and/or functional changes in the brain in CS can be demonstrated with (functional) magnetic resonance imaging ((f)MRI), PET, and somatosensory evoked potentials (SEP) [31–33]. Finally, questionnaires like the Central Sensitisation Inventory (CSI) and Sensory Hypersensitivity Scale (SHS) have been used to detect and measure CS-related symptoms.

Until now these tests have mostly been used by medical specialists and physiotherapists and rarely in general practice, so we aimed to assess which tests might have added value and might be feasible and suitable for use in general practice.

To reach consensus on which tests for CS from our systematic review could be feasible and have added value in general practice, we conducted a Delphi study among various experts [34].

## Methods

**Fig. 1:**
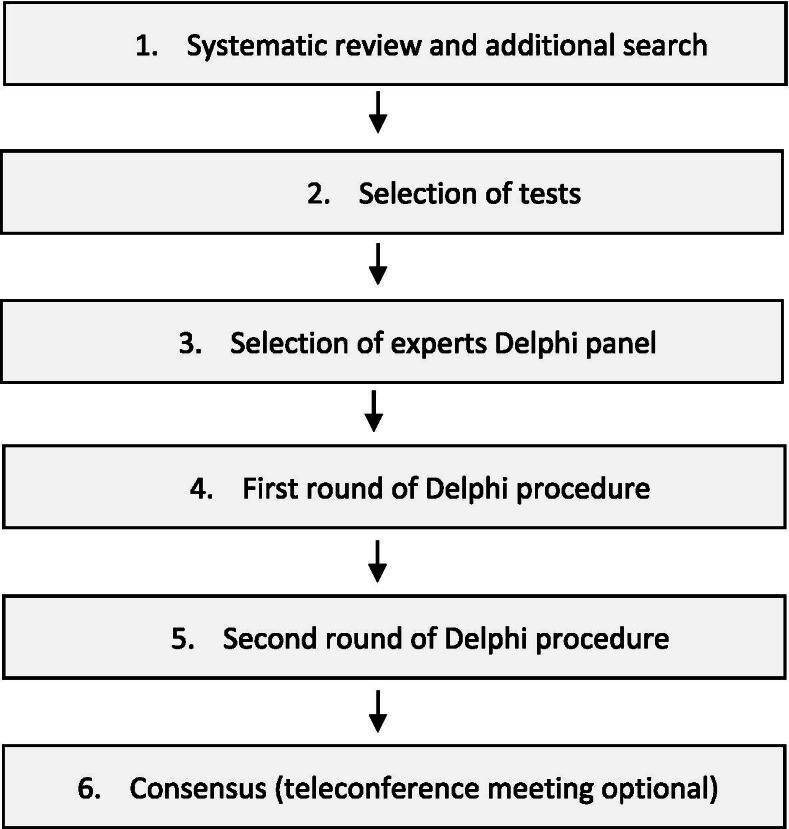
flowchart Delphi study

We provided a flowchart of the Delphi study in Fig. 1.

### Recruitment of participants for the Delphi panel

We compiled a list of potential participants consisting of GPs and other (medical) specialists (e.g. neurologists) with expertise in the domain of chronic pain and/or PPS treatment and research, from the Netherlands as well as from other countries. The authors discussed potential participants based on their professional networks and on a list of authors of CS-related studies. We invited them by email to participate in the Delphi study, offering a modest reward, a credit voucher of 20 euro.

### Selection of tests

We made a list of the tests we retrieved from the literature for our systematic review (Table 2) [1]. These tests comprised mostly physical examination tests but also questionnaires.

**Table 2:** Tests from a previous systematic review

Measurement test	What is measured?	Examples
Quantitative sensory testing (QST)	Hyperalgesia, allodynia, temporal summation	Thermal stimuli: thresholds for cold pain, heat pain, cold detection and heat detection; e.g., putting the hand in an iced water bath Tactile stimuli: pressure pain thresholds (PPTs) Vibratory or vibrotactile stimuli: detection thresholds for vibration or a combination of tactile and vibratory stimuli, e.g., electric toothbrush Electrical stimuli: reaction to electrical pulses with electrodes Distention: distending the rectum or oesophagus with an inflatable balloon Ischemic stimuli: ischemic compression of the arm with a cuff Reaction on specific pain mediators, e.g. reaction on injection with hypertonic saline
Two different quantitative sensory tests together	Conditioned pain modulation (CPM)	Tonic phasic stimulation: phasic heat test with counter irritation of cold Ischemic stimulation: inflating an occlusion cuff, comparing pressure pain prior to and during cuff inflation The nociception withdrawal reflex e.g. H(offman) reflex: stimulation of median nerve with an EMG device, measurement of H wave (a compound muscle action potential) Measurement of the cutaneous silent period (CSP): a brief pause in muscle action potentials following strong stimulation of a cutaneous nerve during a sustained voluntary contraction
MRI^a^, fMRI^a^, PET^a^, somatosensory evoked potentials (SEP)^a^	Structural and functional brain changes	Measurement of changes in brain morphology (global and regional grey matter volumes), changes in density and changes in signalling
Measurement of cytokine levels	Laboratory evaluation	Measurement of serum levels of pro-inflammatory interleukins (Il-1, IL-6, IL-8) and anti-inflammatory interleukins (IL-4, IL-10); serum levels of TNF-alpha, a pro-inflammatory cytokine
Measurement of neurotrophin levels	Laboratory evaluation	Measurement of serum levels of nerve growth factor (NGF) and brain derived neurotrophic factor (BDNF)
Questionnaires	Symptoms, history of functional syndromes	Central Sensitization Inventory (CSI)
	Sensory aspects of hypersensitivity	Sensory Hypersensitivity Scale (SHS)

^a^ Not applicable in general practice, this category was not presented to the panellists

To collect information on test characteristics, we performed additional searches in the PubMed database with search terms covering the name of the test category combined with search terms referring to CS. If there were too many publications (> 100), we restricted the search by adding the search terms ‘specificity OR sensitivity’. For tactile stimulation our first search had 187 hits, after addition of sensitivity OR specificity 7 hits were left. See Table 3 for the search terms in PubMed.

**Table 3:** Search terms PubMed

The search will be conducted per measurement test category.	
“Central Nervous System Sensitization”[Mesh]	
“Central Sensitization” OR “Central Sensitisation” OR “Central Nervous System Sensitization” OR “Central Nervous System Sensitisation”.	
AND	
(all the following search terms will be combined individually with the above-mentioned search terms)	
- Thermal stimulation	
- Tactile stim* OR “Pressure pain threshold*”	
- Vibratory OR vibrotactile stim*	
- Electrical stimuli	
- Distention	
- Ischemic stimulation	
- Hypertonic saline	
- Tonic phasic stimulation	
- Nociception withdrawal reflex OR H reflex OR Hoffman reflex	
- Cutaneous silent period	
- Cytokine*	
- Neurotrophin* OR Nerve growth factor OR NGF OR Brain derived neurotrophic factor (BDNF)	
- Central Sensitization Inventory OR CSI	
- Sensory Hypersensitivity Scale OR SHS	
NOT (“animals”)	
(in case the search provides too many publications, > 100, the following search terms will be added)	
AND	
specif* OR sensit*	

**Fig. 2:**
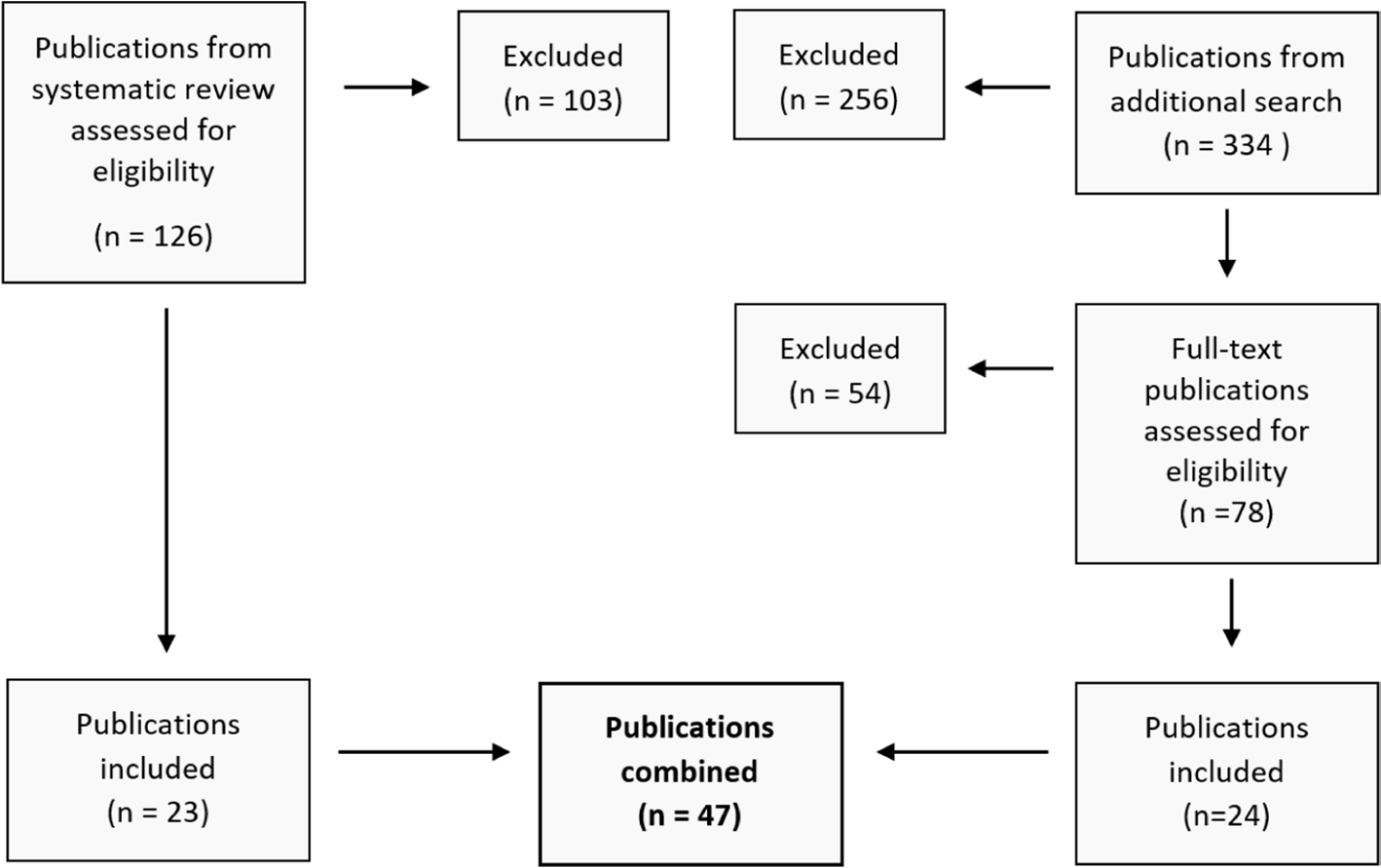
Flowchart publications

We excluded publications that did not focus on CS, were written in other languages than English, German, French or Dutch, were not available in full text or reporting on animal studies. Two persons (CdB and CG) screened the search results and independently selected publications based on title and abstract. These publications were discussed by the project team (Fig. 2, Table 4).

**Table 4:** Articles divided into test categories

Test category	Systematic review	Additional search			
	Included	Hits	Read in full text	Included	Total included
Vibratory stimulation	1	5	2	1	2
Thermal stimulation	0	6	3	2	2
Tactile stimulation	1	7	5	2	3
Electrical stimulation	0	18	3	0	0
Ischemic stimulation	1	38	2	1	2
Ischemic and tactile stimulation	2	18	3	0	2
Tonic phasic stimulation	2	26	6	1	3
Nociception withdrawal reflex	1	18	8	1	2
Cutaneous silent period	2	7	3	1	3
Cytokines	2	19	12	2	4
Neurotrophines	3	88	12	2	5
Central Sensitization Inventory	7	69	19	11	18
Sensory Hypersensitivity Scale	1	15	0	0	1
Total	23	334	69	24	47

We identified twelve tests for CS that might be useful and feasible in general practice. We excluded tests that are, in general, inaccessible or too costly for general practice (brain MRI, fMRI, PET and somatosensory evoked potentials (SEP)) and included tests based on perceived feasibility for general practice: acceptable costs, acceptable execution time and no costly equipment required (Table 5).

**Table 5:** Selected tests for the first round

Test	Description	Category
1	Electric toothbrush	QST
2	Heat and cold	QST
3	Pressure pain thresholds (PPT)	QST
4	Electrical pain and reflex thresholds	QST
5	Ischemia and PPT	CPM
6	Cold and PPT	CPM
7	Heat and cold	CPM
8	Nociceptive flexion reflex (NFR)	Reflexes
9	Cutaneous silent period (CSP)	Reflexes
10	Interleukine 8	Blood test
11	BDNF	Blood test
12	Central Sensitization Inventory	Questionnaire

*QST* quantitative sensory testing

*CPM* conditioned pain modulation

We provided more details about the tests in Table 6.

**Table 6:** Overview test 1–12 First round

Tests 1–4 are quantitative sensory tests (QST) used to measure hyperalgesia, allodynia and temporal summation (TS). These tests comprise different kinds of stimulation.	
Test 1 (electric toothbrush): this test measures vibratory stimuli, this can cause mechanical and thermal stimulation	
Test 2 (heat and cold): a thermode applies heat and cold stimuli to the body.	
Test 3 (pressure pain thresholds): an algometer performs pressure stimulation on different parts of the body and pressure pain thresholds (PPT) and/or pressure tolerance thresholds are measured.	
Test 4 (electrical pain and reflex thresholds): after applying electrical stimuli, electrical pain and reflex thresholds and intensity levels of sensations are measured.	
Tests 5–7 are two different QST tests together to measure conditioned pain modulation (CPM). In CS we expect a smaller reduction of the perceived intensity of the test-stimulus due to reduced inhibition of descending control.	
Test 5 (ischemia and PPT): inflating an occlusion cuff, comparing pressure pain prior to and during cuff inflation.	
Test 6 (cold and PPT): applying cold water in a bucket, comparing pressure pain prior to and during the application of cold water.	
Test 7 (heat and cold): applying cold water in a bucket, comparing pain of heat stimulation prior to and during the application of cold water.	
Test 8 and 9 are tests, which like tests 5–7, reflect dysfunction of the ascending and descending pathways. Test 8 (the nociceptive flexion reflex (NFR)): a protective withdrawal reflex, it measures an increase in the activation of the proximal muscles and this can be considered as a marker of delayed inhibition. The NFR is recorded with an EMG device, mostly on a nerve in the calf (sural nerve) and electromyographic responses are recorded using a pair of surface electrodes placed over the tendon of a muscle in the upper leg (biceps femoris) on the same side.	
Test 9 (the cutaneous silent period (CSP)): this test shows the delayed inhibition of the grasping muscle and this also can be seen as a marker of this delayed inhibition [35, 36]. The CSP is a brief pause in muscle action potentials following strong stimulation of a cutaneous nerve during a sustained voluntary contraction.	
Test 10 (interleukin 8 (IL-8)): this is a laboratory test of the level of the cytokine IL-8. Increased serum levels of cytokines as TNF-alpha and pro-inflammatory interleukins (Il-1, IL-6, IL-8) and reduced anti-inflammatory interleukins (IL-4, IL-10) might lead to neuroinflammation, e.g. of the glia cells, and thus are indicative of dysregulation of the immune system in relation to CS [8]. We expect an increased serum level of IL-8 in patients with CS-related symptoms and this can be measured with a blood sample that has to be processed in a lab.	
Test 11 (brain-derived neurotrophic factor (BDNF)): this is a laboratory test of the level of the neurotrophin BDNF. An increase of neurotrophins, e.g. glutamate, substance P, calcitonin gene-related peptide (CGRP) and BDNF, is described as characteristic of CS. In this test BDNF is analyzed in a blood sample, the lab needs equipment for ELISA processing [26].	
Test 12 (the Central Sensitization Inventory (CSI)): a self-report questionnaire with two parts. Part A consists of 25 statements relating to current health symptoms, rated on a 5-point scale, resulting in 0-100 points. Part B (which is not scored) asks if one has previously been diagnoses with one or more specific disorders, including seven separate central sensitivity syndroms (CSS) like fibromyalgia, chronic fatigue and irritable bowel syndrome and three CSS-related disorders. It is used both as screening instrument and as treatment outcome measure to identify CS-related symptoms in patients [37, 38]. A cut-off point of 40 is generally used to differentiate between healthy controls and persons with CS-related symptoms.	

Selected panellists in the Delphi procedure received by email a list of the 12 tests in a questionnaire with an appendix (Appendix 1 and 2).

For each test the following concise information was provided in the questionnaire:

1. background of the test;
2. procedure of the test;
3. investigated populations;
4. Results

For each test the appendix provided the following additional information:

1. abovementioned information for each individual study
2. materials needed for the test;
3. availability of materials needed for the test;
4. burden for patient;
5. time needed to apply the test;
6. ability of an assistant or practice nurse needed to perform the test;
7. reference list.

The panellists were asked to rate (with a yes/no/?) on two different aspects: firstly, the technical feasibility of the test, and secondly, its added value for general practice. Finally, the panellists were asked to provide an overall judgement on the suitability of the test in general practice. They could motivate their rating or refrain from answering in case of insufficient expertise with regard to a particular test. In this round, they could also provide suggestions for additional tests and general remarks. We selected from the suggestions three additional tests and send these in an additional survey in round 1 (Appendix 3).

Participants were asked to complete and return the score form by e-mail within 2 weeks. The researchers summarized the forms returned by the participants. We defined that consensus was reached if 70% or more of the participants (who had returned the form) agreed selecting a test as suitable or not suitable for general practice. If no agreement was reached on a test (less agreement than the 70% threshold), this test was added to the list for the second round of our Delphi study.

### Second round

Only tests not reaching the threshold of 70% agreement in the first round were included in the second round. Each of these tests was presented with information on the percentage of agreement per item in the first round and a summary of participant comments (Appendixes 4 and 5). In this round, participants could change their rating of a test or motivate their decision again, both in view of the group’s scores. Participants were asked to complete a final score and return it via e-mail within 2 weeks.

As in the first round, consensus was considered to be reached if 70% or more of the participants who had returned the form agreed the test was suitable or not suitable for general practice. Tests for which in the second round less than 70% agreement was reached, were put on a new list for the third round.

### Third round

A third round was planned in case of remaining disagreements. However, we decided to cancel the third round for good reasons, see the results.

### Comments of the panellists

In order to provide an overview of the comments of the panellists, we performed a qualitative analysis. Two researchers (CdB, JCvdW) independently listed the comments and coded all relevant items with the program ATLAS.ti version 8.0. We categorized the codes into families and performed a thematic analysis of the data.

## Results

### Delphi study

Off the invited 40 participants, 26 agreed to participate. In the first round, a few panellists recommended additional experts of whom we invited three, one agreed to participate. Thus, the final panel consisted of 27 experts. 21 panellists were Dutch and 12 panellists were GPs (Appendix 6). In the first round, panellists had the opportunity to add tests. Nine panellists suggested 18 additional tests; after analysis and discussion in the research group, we decided to add the monofilaments, the clothes peg and the Sensory Hypersensitivity Scale. As in our first selection of tests, we selected tests that might be useful and feasible for general practice and excluded tests that are, in general, inaccessible or too costly for general practice.

We added these three tests recommended by the panellists in an extension of the first round, making a total of fifteen tests. We provided the information in the same way as for the other 12 tests, an overview of test 13–15 can be found in Table 7.

**Table 7:** Overview test 13–15

Test 13 (monofilaments): a QST test to assess temporal summation and slowly repeated evoked pain (SREP) using monofilaments of different weights [9]. A monofilament is a thick thread which gives a pricking sensation when applied to the skin. To measure temporal summation, the monofilament is applied several times with a time interval, e.g. 10 times with a time interval of one second. In case of CS we expect a stronger increase in pain after applying the monofilament then in healthy controls due to temporal summation.	
Test 14 (clothes peg): a QST test with a calibrated clothes peg to measure pressure pain [39]. A calibrated clothes peg is applied for 10 s and patients rate the pain intensity on a 0 to 10 numerical rating scale, the clothes peg is applied on the middle fingers or earlobe. In case of CS we expect a higher pain intensity then in healthy controls.	
Test 15 (the Sensory Hypersensitivity Scale (SHS)): a 25-item self-report measure of sensory hypersensitivity. The SHS assesses both general sensitivity and modalityspecific sensitivity (e.g. touch, taste, and hearing) [40].	

### First round

In the first round the panel reached consensus on eight of the fifteen tests: one to be included and seven to be excluded (Table 8).

**Table 8:** Results Delphi study

Instrument	1st round				2nd round				
	Yes	no	?	result	yes	no	?	result	decision
1.Electric toothbrush	11	14	1	Inconclusive	5	21		81% no	Excluded
2.Heat and cold	11	13	2	Inconclusive	2	24		92% no	Excluded
3.Pressure pain thresholds (PPT)	15	9	3	Inconclusive	20	7		74% yes	Included
4.Electrical pain and reflex thresholds	4	21	1	81% no					Excluded
5.Ischemia and PPT	8	17	1	Inconclusive	5	21		81% no	Excluded
6.Cold and PPT		27	1	96% no					Excluded
7.Heat and cold	6	19	1	73% no					Excluded
8.Nociceptive flexion reflex (NFR)	5	20	1	76% no					Excluded
9.Cutaneous silent period (CSP)	2	24		92% no					Excluded
10.Cytokine levels	5	19	2	73% no					Excluded
11.Neurotrophin levels	4	22		84% no					Excluded
12.Central Sensitization Inventory (CSI)	21	4	2	81% yes					Included
13.Monofilaments	16	7	2	Inconclusive	18	8		69% yes	Included^a^
14.Clothes peg	13	12		Inconclusive	3	23		88% no	Excluded
15.Sensory Hypersensitivity Scale (SHS)	11	13	1	Inconclusive	13	12	1	52% yes	Inconclusive

?: doubt or no expertise

^a^69% was so close to 70% that we decided to include the test

Arguments in favour of the one included test, CSI, were: easy to use, cheap, accessible, to have good test characteristics and ‘the best so far’. However, the panellists were critical on the construct validity of the CSI.

Seven tests were excluded in this round. The electrical pain and reflex thresholds tests were discarded mostly because electrical stimuli were considered unattractive to apply to patients; an EMG device is expensive and was considered to be too complex for use in general practice.

The CPM test with the combination of cold and pressure pain thresholds was unanimously discarded because of the following arguments: the test is complex, expensive, the use of ice water is messy and the temperature is difficult to control.

Three- quarter of the panellists discarded the CPM test with the combination of heat and cold stimuli because the thermosensory unit is very expensive and again the use of ice water was considered to be messy.

The nociceptive flexion reflex (NFR) and the cutaneous silent period (CSP) were considered to be valid tests but discarded because an EMG is too expensive for general practice. Maybe this test could be made available as a diagnostic test in a hospital or diagnostic centre for primary care.

Some panellists found assessing cytokine and neurotrophin levels in blood useful while others stated that these tests are not specific enough as a test of CS; moreover, the tests are not available yet in general practice.

### Second round

One test was included in the second round, the pressure pain threshold (PPT) test. Some panellists changed their minds based on additional information on the reliability and costs of the handheld algometer. A digital algometer was considered to be too expensive, but a handheld algometer of 200 euros was considered reasonable.

Four tests were excluded in this round. Panellists discarded the electric toothbrush test because the validity of the test is not clear and hygiene is an issue. The painful heat or cold stimuli test appeared, due to additional information of the panellists, to be more expensive than we had stated in the first round, so it was discarded because of the costs and complexity of the test. The clothes peg was discarded because it is not sufficiently studied and the panellists found this test not professional, it lacks face validity. The CPM test with combination of ischemic stimuli and pressure pain thresholds was discarded because it was considered too complex for use in general practice.

### Inconclusive

For two tests the panel reached no consensus in the second round. For the monofilaments the panel almost reached ‘positive’ consensus (69%). Because 69% consensus for monofilaments was so close to 70% we considered this test to be included.

No consensus was reached for the sensory hypersensitivity scale (SHS), 52% scored yes. Arguments pro were that the questionnaire is cheap and measures more specific items than the CSI. Arguments contra were that the SHS is not available and validated in Dutch, correlations with other measures are low and the CSI is validated better. There were little shifts in arguments and judgements between round 1 and 2. As we did not expect a third round would produce important new information, we decided to cancel this round.

Finally, we included three tests: the CSI in the first round, the “pressure pain thresholds” test (PPT) and monofilaments in the second round; these tests were considered to be suitable for use in general practice.

### Expert comments

The analysis of the free text comments provided by the panellists were categorized into ten themes (Table 9).

**Table 9:** Themes and number of codes of each theme

Themes	Number of codes
Performance	202
Test characteristics	155
Costs	129
Time	91
Burden for patient	30
Reference values	19
Specific test materials	16
Test population	14
Follow up	3

Most mentioned themes were the performance of the test, test characteristics, costs and time. Panellists agreed on easy performance of the tests, e.g. electric toothbrush, PPTs, taking blood samples, questionnaires and the use of monofilaments and the clothes peg. Electrical pain thresholds were considered difficult to perform. CPM tests scored equally positive and negative on performance, especially the GPs in the panel found the combination of tests too complex and messy.

Test characteristics were mostly mentioned when they were unclear, e.g. in the electrical pain and reflex threshold test and in almost all CPM tests.

Costs were an issue, especially when the costs were high the panellists were negative about the test. This was particularly the case in tests where an expensive EMG device was needed, as in the nociception flexion reflex (NFR), the cutaneous silent period (CSP) and the electrical pain and reflex thresholds.

Time was frequently mentioned and here again we saw a difference in opinions between the GPs in the panel and other panellists like physiotherapists and medical specialists. Due to the consultation time of GPs, which is 10–20 min, tests should not take more time than 10 min. Especially the GPs from the UK, where the consultation time is often limited to 10 min, found most tests too long for performing in GP practice.

Burden for the patient was an issue when the test was unpleasant, as in the electrical pain and reflex threshold test.

Reference values were mentioned as a negative argument when they were not available. Specific test materials needed was only mentioned in the tests with an EMG device. Test population was mentioned as a negative argument when it was too specific, as in the electric toothbrush test only patients with temporomandibular disorder were tested. The possibility of follow up of the patient was mentioned as a positive argument, e.g. with the CSI.

### General remarks

Many panellists used the opportunity to provide general remarks. Some panellists were very critical, they considered the tests more as research tools, not yet ready for application in general practice and only interpretable on group level but not in an individual patient. Other panellists even questioned CS in general and its relation to PPS. And some doubted the added value above good history taking and physical examination and were afraid of medicalisation of the symptoms. Finally, there was criticism on the information provided to the panellists, there was not enough information provided on reliability and validity of the tests.

Other panellists were very enthusiastic about the idea of applying these tests in general practice. They reported that they used the tests themselves, that a battery of tests was better than a single test and were happy with all the provided information on the tests.

## Discussion

We aimed to obtain consensus from experts on which tests to measure CS could be feasible and suitable to use in general practice. In two rounds we reached consensus on fourteen of the fifteen tests: eleven were discarded, the CSI, PPTs and monofilaments were included. After the second round, we did not reach consensus on one test, but as we did not expect a third round would produce important new information, we decided to cancel this round and discarded the test.

### Scientific evidence for the three included tests

We included the CSI, PPTs and monofilaments.

The CSI is a self-report questionnaire that has been validated in several studies and that can be used both as a screener and as treatment outcome measure [37, 41–46]. The CSI generates reliable and valid data and is able to quantify the severity of CS symptoms [38]. At the time of this writing it has been translated into 19 different languages, is freely online available on www.pridedallas.com and a user manual is available [47].

Nevertheless, there are disadvantages of the CSI. First, it was developed to measure central sensitivity syndromes (CSS), which is not quite the same as central sensitization. CSS are syndromes like fibromyalgia, irritable bowel syndrome and chronic fatigue syndrome. These syndromes share common symptoms and CS is one of the underlying mechanisms in the development of CSS, but not the only one [48–50]. Secondly, some of the panellists expressed doubts about the construct validity of the test. The items of the CSI measure different constructs, like physical, psychological and cognitive functioning, physical symptoms and others. These constructs are probably related to CS, but further research is needed to establish the validity of the CSI [51].

PPTs are widely used to measure CS and are validated well [25, 52–54]. In general practice measurement of pressure pain thresholds seems more appropriate than pressure tolerance thresholds. The PPT is the minimum force applied to induce pain and is measured with an algometer or dolorimeter, which is a calibrated force gauge. A handheld algometer is cheap, the procedure is short, around 10 min, and fits in the normal consultation schedule of the GP.

Monofilaments are thick nylon threads of different thicknesses and can be used to measure temporal summation, which is specific for CS. GPs use the monofilaments also to assess lower extremity neuropathy in patients with diabetes. They are cheap and the time to perform the test, 10 min, fits in the schedule of the GP. There is scant research on the use of monofilaments to assess CS, but the available studies have convincing results, e.g. for slowly repeated evoked pain (SREP) a sensitivity of 0,89 and specificity of 0,87 in discriminating between fibromyalgia and rheumatoid arthritis patients [9, 53, 55].

### Strengths and limitations

We invited 43 panellists, 27 (63%) agreed to participate and all 27 panellists completed all surveys. Among the panellists, we had 12 general practitioners, 8 physiotherapists and 7 participants from other disciplines. Less than half of the panellists were GPs, this might be considered as a limitation. But as GP’s have little experience with the application of these tests, we decided to invite also a number of medical specialists who frequently use these tests in practice and some physiotherapists. A strength is that most panellists have both research experience and clinical experience in the field of PPS or chronic pain.

We invited 27 panellists from the Netherlands and 16 from 9 other countries. In the final panel 21 panellists from the Netherlands and 6 from 4 other countries participated. So we had more refusals from international experts. The GPs from the Netherlands and UK were involved in PPS, chronic pain and/or research, their opinion might not be representative for the GPs as a group.

We excluded tests that were considered to be too expensive or not available in general practice, like (f)MRI, PET and sensomotory evoked potentials (SEP). However, we could have added SEP as a test to assess CS; SEP is evoked with an electrical stimulus of a peripheral nerve and potentials are measured [56–58].

Because the presented tests are not used yet in general practice, the GPs did not have clinical experience with the tests and had to answer from a theoretical point of view. Other panellists had more experience with the presented tests, but were less critical on time constraints and costs, e.g. physiotherapists have more time and hospitals have more money to spend on needed devices.

Whereas the CSI and PPTs have a solid base in the literature, monofilaments are less well-studied.

More research is needed to establish their characteristics to assess CS.

Note that the choices for the most suitable tests were based solely on expert opinion about what is practical in general medical practice, not which ones were perceived to be the best at diagnosing CS. We will conduct a study to assess whether GPs are able to apply the tests to their patients in practice and investigate their experiences with the tests.

### Recommendations for further research

We do not yet know whether the tests are sensitive and specific enough to diagnose CS on an individual level. The tests are mostly used on population level and further research is needed to assess if and how these tests are transferable to clinical practice with regard to interpretation, practicality and feasibility.

More research is also needed to assess whether the characteristics of these tests, as test-retest reliability and temporal stability, are sufficient when the tests are applied by general practitioners in a primary care population [55]. Additionally, further research should establish reference values, cut-off points and assessment of the construct validity of the tests) [59].

## Conclusion

After a consensus study among an international panel of 27 experts, three tests for measuring CS were considered to be potentially feasible and suitable to be used in general practice: the Central Sensitization Inventory (CSI), pressure pain thresholds (PPTs) and monofilaments. It is worthwhile to conduct further research on the feasibility of these tests in general practice because they might have additional diagnostic value and offer an acceptable explanation for PPS.

## Supplementary Information

**Additional file 1: Appendix 1.** Survey first round

**Additional file 2: Appendix 2.** Appendix first round

**Additional file 3: Appendix 3.** Survey and appendix additional tests first round

**Additional file 4: Appendix 4.** Survey second round

**Additional file 5: Appendix 5.** Appendix second round

**Additional file 6: Appendix 6.** List of participants

## Data Availability

The datasets used and/or analysed during the current study are available from the corresponding author on reasonable request.
