# Supplementary material for: Tests for central sensitization in general practice: a Delphi study
Source: BMC Fam Pract. 2021 Oct 19;22:206. doi: 10.1186/s12875-021-01539-0 (PMC8527602; doi:10.1186/s12875-021-01539-0)
Supplement: Supplementary file 1 — Additional file 1: Appendix 1. Survey first round [file 12875_2021_1539_MOESM1_ESM.docx]

Appendix 1: survey first round

Tests for central sensitization in general practice: a Delphi **study**

Carine den Boer, MD^1^

Berend Terluin MD, PhD^1^
Johannes C. van der Wouden PhD^1^
Annette H. Blankenstein MD, PhD^1^
Henriëtte E. van der Horst MD, PhD^1^

1. Amsterdam UMC, location VUmc, Department of General Practice, Amsterdam Public Health research institute, the Netherlands.

Correspondence:

C. den Boer

Amsterdam UMC, location VUmc

Department of General Practice

Amsterdam Public Health research institute

Van der Boechorststraat 7

1081 BT Amsterdam

The Netherlands

Telephone: +31613693267

Email: [c.denboer@amsterdamumc.nl](mailto:c.denboer@amsterdamumc.nl)

Appendix 1: survey first round

Amsterdam, 25 January 2019

Dear participant,

Many thanks for accepting our invitation to participate in our Delphi procedure. Aim of the procedure is to reach consensus on measurement instruments for central sensitization suitable for use in general practice. This letter provides a brief outline of the project.

In this document, we have summarized the information about the instrument and their test characteristics. We have provided additional background information in the appendix.

In our systematic review on CS (attached as PDF) we identified a number of CS measurement instruments. We carried out an additional PubMed search to retrieve additional information on the instruments and to search for additional instruments**.**

We identified twelve tests for CS that may be suitable for general practice. We excluded the tests that are inaccessible or too costly for general practice (e.g., brain MRI, fMRI, PET and somatosensory evoked potentials, sensory hypersensitivity scale ).

For each of the measurement instruments, we provide a summary of relevant information and kindly ask you to rate each measurement instrument (with a +/-/?) on two different aspects:
1. **Technical feasibility: how would you rate the feasibility of the test in a general practice setting;
2. Added value of the test: what is your opinion about the added diagnostic value of a test?**We would appreciate if you could briefly motivate your ratings.

We will summarize the results and assess whether each instrument reaches at least 70% agreement after each round. We have two email rounds and when necessary a teleconference as third round. Extra information about the Delphi procedure is provided in the attached appendix.

If you have any questions, don’t hesitate to contact us.

We hope you can find the time to respond within two weeks, before 10 February, so we can prepare the second round, which you will receive half February or soon afterwards.

Warm greetings,

Carine den Boer, MD GP, email: [c.denboer@vumc.nl](mailto:c.denboer@vumc.nl);, mobile phone 0031613693267

Christijan Georgiev, medical student, email: c.georgiev@student.vu.nl
Amsterdam UMC, Vrije Universiteit Amsterdam
Department of general practice and elderly care medicine

Amsterdam Public Health research institute, the Netherlands

Introduction Delphi procedure

We are asking you to rate twelve tests.

Tests 1-4 are quantitative sensory tests (QST). QST is used to measure hyperalgesia (increased sensitivity to painful stimuli), allodynia (painful perception of non-painful stimuli) and temporal summation (TS). TS refers to the phenomenon of increased pain perception in response to repetitive noxious stimuli over time (ascending facilitation, referring to central sensitization). Hyperalgesia and allodynia can also refer to peripheral sensitization, but when measured on another part of the body it refers to CS.

Test 5-7 are tests of conditioned pain modulation (CPM). CPM refers to the phenomenon that ‘pain inhibits pain’, the reduction in pain sensitivity for a tested stimulus due to the interference of a second stimulus (conditioning stimulus) applied at the same time but to a remote body region (figure). CS leads to a smaller reduction in pain sensitivity due to hyperexcitability of the central nervous system (CNS) and reduction of descending inhibition.

Test 8 and 9 are tests which also refer to dysfunction of the ascending and descending pathways. The nociceptive flexion reflex (NFR) is a protective withdrawal reflex and upregulation can be considered as marker of dysregulation of descending control. There is not only an increase in the activation of the proximal muscles (NFR) but also a delay in the inhibition of the grasping muscles, the cutaneous silent period (CSP) can be seen as a marker of this delay of inhibition.

Test 10 refers to dysregulation of the immune system, especially the cytokines, in relation to CS. Higher serum levels of TNF-alpha and pro-inflammatory interleukines (Il-1, IL-6, IL-8) and a reduction of anti-inflammatory interleukines (IL-4, IL-10) might lead to neuroinflammation, e.g. of the glia cells.

Test 11 refers to dysregulation of neurotrophines in relation to CS. An increase of neurotrophines, e.g. glutamate, substance P, CGRP and BDNF is described as operationalization of CS.

Test 12 is a questionnaire. The Central sensitization inventory (CSI) is used as screening instrument to identify CS in patients.

1. Electric toothbrush test

Background:

Producing vibrotactile stimuli by an electric toothbrush can cause mechanical and thermal stimulation, leading to temporal summation (TS).

Method:

An electric toothbrush is used to apply vibrotactile stimuli to various areas of the head and arms, 1 pound pressure for 30 seconds in four different areas. Pain intensity is recorded at 0, 15, 30 and 60 seconds on a 0 to 10 scale (<1 minute in total). The test takes 8 minutes (less than 2 minutes per stimulus area).

Investigated population:

Women with temporomandibular disorders (TMD), chronic orofacial pain and arthralgia.

Results:

Sensitivity of 57% and specificity of 92% over all four areas, in one of the tests. Patients had higher pain sensitivity and lower pressure point threshold values compared to the control group.

| Electric toothbrush test | Score (+,-,?) | Motivation |
| --- | --- | --- |
| Technical feasibility |  |  |
| Added value test |  |  |
| Overall judgment: suitable for use in general practice? YES/NO | | |

1. The painful heat or cold stimuli test

Background:

Repetitive painful heat or cold stimuli may induce TS. Perception thresholds are assessed, pain thresholds (when is the stimulus experienced as painful) and pain tolerance thresholds (when is the pain felt as intolerable). Lower pain thresholds to cold and hot stimulation have been found in FM patients, suggesting hyperexcitability of the nociceptive system.

Method:

Painful heat and cold test: a thermode applies heat and cold stimuli to the hands and shoulders. The temperature is adjusted and participants are instructed to rate their pain on an visual analogue scale (VAS). Perception and pain thresholds are assessed.

For this test a thermoregulatory unit is needed, this consists of a computer which generates thermal stimuli and a Peltier electrode for administering the stimuli to the skin, and costs circa 150 euro. The patient experiences pain with a mean VAS of 3 (on a scale of 10).

Investigated population:
Patients with fibromyalgia (FM), local (neck/shoulder) musculoskeletal pain (LMP) and controls

Results:
Heat tests: the studies showed that this test can distinguish between FM and local musculoskeletal pain and between these and control patients.

Cold-heat test: significantly lower pain threshold to cold and hot stimulation in FM patients.

| The painful heat or cold stimuli test | Score (+,-,?) | Motivation |
| --- | --- | --- |
| Technical feasibility |  |  |
| Added value test |  |  |
| Overall judgment: suitable for use in general practice? YES/NO | | |

1. Pressure pain thresholds (PPT) and pressure tolerance thresholds

Background:

Most QST studies use pressure pain thresholds (PPTs) for measuring central sensitization. Pressure pain detection threshold is set at a point where comfortable pressure turns into pain; the pressure pain tolerance threshold is set at a point where the pain is felt intolerable.

Method:
A pressure algometer performs pressure stimulation on different parts of the body. PPTs are measured by increasing pressure. The patient scores the pain on a VAS scale. The patient can press a button when the pain becomes too much. A pressure algometer can be manual (circa 200 euro) or computer-controlled (circa 2000 euro).

Investigated population:

Patients with FM and chronic pain patients.

Results:
Patients with FM had an increased pain perception intensity compared to control patients. Using PPT as measure of pain hypersensitivity had a good ability in distinguishing both chronic neck pain patients and chronic low back patients from healthy control subjects.

| Pressure pain thresholds (PPT) and pressure tolerance thresholds | Score (+,-,?) | Motivation |
| --- | --- | --- |
| Technical feasibility |  |  |
| Added value test |  |  |
| Overall judgment: suitable for use in general practice? YES/NO | | |

1. The electrical pain and reflex thresholds

Background:

Electrical pain and reflex thresholds are measures of central pain sensitivity. One potential application of these assessments in clinical practice is the detection of central hyper- or hyposensitivity in individual patients. In order to identify these disturbances in the central pain processing of individual patients, knowledge of reference values is essential.

Method:

Pain thresholds and intensity level of sensations are measured after (repeated) electrical stimulation using a VAS scale.

Electrical stimuli are applied with an electrode to the sural nerve in the calf (with a time interval of 6-10 seconds). The withdrawal response is measured, using an electromyography device (EMG), in the tendon of the biceps femoris on the same side of the body, above the calf where the electrical stimulus is applied. For the patient an electrical stimulus can give an uncomfortable sensation. For the test an EMG device is necessary which costs between 750 and 2000 euro.

Investigated population:

Patients with FM, whiplash-associated disorders (WAD) and controls. Reference values in healthy controls are available (study with 300 healthy controls).

Results:
Patients with FM and WAD, both in acute and chronic phase, had lower withdrawal responses, lower pain thresholds and higher levels of sensation to a certain electrical stimulus compared to controls.

| The electrical pain and reflex thresholds | Score (+,-,?) | Motivation |
| --- | --- | --- |
| Technical feasibility |  |  |
| Added value test |  |  |
| Overall judgment: suitable for use in general practice? YES/NO | | |

5. CPM: combination of ischemic stimuli and PPT

Background:

Conditioned pain modulation (CPM) means that a test-stimulus and a conditioning stimulus are used together in the test. A combination of ischemic stimuli and PPTs is used here as CPM. In healthy controls the conditioning stimulus leads to a reduction in the perceived intensity of the test-stimulus. In central sensitization there is a smaller reduction of the perceived intensity of the test-stimulus due to reduced inhibition of descending control.

Method:

The QSTs which are used for CPM combine the following: pressure pain thresholds (PPTs) by an algometer; ischemic stimulation by occlusion cuff (also used for blood pressure measurement) inflated on the arm to a painful intensity (VAS 3 or 4).

Investigated population:
Patients with knee osteoarthritis (KOA), chronic patients with whiplash associated disorder (WAD) and healthy controls.

Results:

TS of pressure pain was significantly decreased in healthy controls compared to patients with chronic WAD. In contrast, TS was quite similar prior to and during cuff inflation. This provides evidence for dysfunctional CPM in these patients. Lack of endogenous pain inhibitory pathways provide additional evidence for presence of CS.

Another study showed a significant increase in PPT during cuff stimulation in controls. These patients with KOA had significant facilitation of TS and significantly less CPM as compared with controls.

| CPM: combination of ischemic stimuli and PPT | Score (+,-,?) | Motivation |
| --- | --- | --- |
| Technical feasibility |  |  |
| Added value test |  |  |
| Overall judgment: suitable for use in general practice? YES/NO | | |

6. CPM: combination of PPT and cold pain

Background:

Conditioned pain modulation (CPM) means that a test-stimulus and a conditioning stimulus are used together in the test. A combination of cold pain stimulation and PPTs is used here as CPM. In healthy controls the conditioning stimulus leads to a reduction in the perceived intensity of the test-stimulus. In central sensitization there is a smaller reduction of the perceived intensity of the test-stimulus due to reduced inhibition of descending control.

Method:

The QSTs which are used for this CPM test combine the following: PPTs by an algometer to a verbal pain score of 4 out of 10; cold pain stimulation applied by immersing the foot in a bucket of ice water or a neurosensory analyser to a verbal pain score of 6 out of 10.

Investigated population:
Patients with chronic non-specific low back pain (CLBP), KOA and controls

Results:

Patients with CLBP had significant decrease of PPT compared to controls (in CPM). The PPT in controls increased. In contrast, by differentiating in sex, men had no significant difference in PPTs compared to women. This suggesting that the difference is only driven by changes in women, not in men, indicating an enhanced widespread sensitivity in women. Another study showed negative values in CPM, indicating pain inhibition and positive values indication pain facilitation in the patients. Patients with positive values were suggested to be linked with CS.

| CPM: combination of PPT and cold pain | Score (+,-,?) | Motivation |
| --- | --- | --- |
| Technical feasibility |  |  |
| Added value test |  |  |
| Overall judgment: suitable for use in general practice? YES/NO | | |

7. CPM: combination of heat and cold stimuli

Background:

Conditioned pain modulation (CPM) means that a test-stimulus and a conditioning stimulus are used together in the test. A combination of heat stimulation and cold stimulation is used here as CPM. In healthy controls the conditioning stimulus leads to a reduction in the perceived intensity of the test-stimulus. In central sensitization there is a smaller reduction of the perceived intensity of the test-stimulus due to reduced inhibition of descending control.

Method:

The QSTs which are used for this CPM test combine the following: heat stimulation by a thermode and cold stimulation by cold water in a bucket.

Investigated population: patients with IBS, premenopausal female controls, chronic musculoskeletal pain and healthy controls.

Results:
Patients with IBS had a smaller CPM compared to controls, demonstrating a deficient descending control. Another study showed CPM serving as marker, indicating impairment of the descending pain-modulatory system in patients with chronic pain.

| CPM: combination of heat and cold stimuli | Score (+,-,?) | Motivation |
| --- | --- | --- |
| Technical feasibility |  |  |
| Added value test |  |  |
| Overall judgment: suitable for use in general practice? YES/NO | | |

8. The nociceptive flexion reflex (NFR)

Background:

The nociceptive flexion reflex (NFR) is a protective withdrawal reflex and upregulation can be considered as marker of dysregulation of descending control.

Method:

The NFR is recorded with an EMG device, mostly on a nerve in the calf (sural nerve) and electromyographic responses are recorded using a pair of surface electrodes placed over the tendon of a muscle in the upper leg (biceps femoris) on the same side. The R‐III reflex (objective threshold) is identified as a multiphasic signal appearing at least 90 msec but less than 250 msec after each stimulation. An EMG device is needed with surface electrodes and a trained assistant or practice nurse. The test takes 15 minutes.

Investigated population:

Patients with fibromyalgia and controls

Results:

The median NFR threshold in patients with FM (22.7 mA [range 17.5-31.7]) is significantly decreased compared with that in controls (33 mA [range 28.1-41]). Diagnostic test performance: a cut-off value of <27.6 mA for NFR provides sensitivity of 73% and specificity of 80% for detecting central allodynia in the setting of FM.

| The nociceptive flexion reflex (NFR) | Score (+,-,?) | Motivation |
| --- | --- | --- |
| Technical feasibility |  |  |
| Added value test |  |  |
| Overall judgment: suitable for use in general practice? YES/NO | | |

9. The cutaneous silent period (CSP)

Background:

The NFR and cutaneous silent period (CSP) are excitatory and inhibitory parts of the same spinal protective reflex, respectively. The CSP is a brief pause in muscle action potentials following strong stimulation of the cutaneous nerve during a sustained voluntary contraction, and is considered a protective reflex mediated by the spinal inhibitory circuit and reinforced by parallel modulation of the motor cortex.

Method:

The CSP is recorded in the upper and left lower extremities or measured from the abductor pollicis brevis muscle evoked by electrical stimulation of the right fifth digit. An EMG device is needed with surface electrodes and a trained assistant or practice nurse. The test takes 15 minutes. The burden for the patient is low.

Investigated population:

Fibromyalgia patients and controls

Results:

In central sensitization the CSP has prolonged latencies and a longer duration.

| The cutaneous silent period | Score (+,-,?) | Motivation |
| --- | --- | --- |
| Technical feasibility |  |  |
| Added value test |  |  |
| Overall judgment: suitable for use in general practice? YES/NO | | |

10. Cytokine level in blood samples

Background:

Cytokines are proteins with an effect on other cells on communication and interaction. There are two categories of cytokines, pro-inflammatory and anti-inflammatory cytokines. Higher serum levels of tumor necrosis factor-alpha (TNF-α) and pro-inflammatory interleukines (Il-1, IL-6, IL-8) and a reduction of anti-inflammatory interleukines (IL-4, IL-10) might lead to neuroinflammation, e.g. of the glia cells. These changes are also associated with several components of sickness behaviour and have been found changed in chronic pain and MUS.

Method:

Collecting a blood sample and measurement of TNF-alpha and interleukines, e.g. IL-8, a pro-inflammatory cytokine at different moments during treatment. The blood sample has to be processed in a lab which has the equipment to measure interleukine-8. Not all labs offer this. Low burden for patients. Costs dependent of the lab.

Investigated population:

Fibromyalgia patients

Results:

Patients with chronic pain have increased TNF-alpha levels compared to controls.

IL-8 is increased in patients with FM, the level can reduce significantly during multidisciplinary treatment of FM. After successful treatment, circulating IL-8 level can be in the normal range.

| Cytokine level in blood samples | Score (+,-,?) | Motivation |
| --- | --- | --- |
| Technical feasibility |  |  |
| Added value test |  |  |
| Overall judgment: suitable for use in general practice? YES/NO | | |

11. Neurotrophin level in blood samples

Background:

Neurotrophines are proteins, regulating neuronal function and affecting the growth and survival of neurons, in the peripheral and central nervous system. In chronic (musculoskeletal) pain and overactive bladder, serum or urine levels of neurotrophines such as nerve growth factor (NGF) and brain derived neurotrophic factor (BDNF) have been found to be increased.

Method:

A blood sample is analyzed for BDNF. The lab needs equipment for ELISA processing, not all labs offer this test. Time for processing depends on the lab. Burden for patient is low. Costs dependent of the lab.

Investigated population:
Fibromyalgia patients, chronic pain patients, controls

Results:

BDNF is inversely correlated with the PPT. Statistically significant correlation between BDNF level and decrease of PPTs. No reference values are available. BDNF levels fluctuate in the body, dependent on time of the day, season and lifestyle factors.

In another study, BDNF and TNF-alpha levels were higher in MUS patients compared to chronic pain patients and normal controls.

| Neurotrophin level in blood samples | Score (+,-,?) | Motivation |
| --- | --- | --- |
| Technical feasibility |  |  |
| Added value test |  |  |
| Overall judgment: suitable for use in general practice? YES/NO | | |

12. Central sensitization inventory (CSI)

Background:

The CSI is a self-report questionnaire that has been validated in several studies and that can be used both as a screener and as treatment outcome measure.

The CSI consists of two parts. Part A consists of 25 statements relating to current health symptoms, rated on a 5-point scale, resulting in 0-100 points. Part B consists of 10 items relating to seven previously diagnosed MUS syndromes, whiplash, anxiety and depression.

In one study a short variant of the CSI, the CSI-9 was described.

Method:

Patients have to fill in the questionnaire, the researcher has to score the questions and interpret the results. The CSI is freely available in 12 different languages , including Dutch, on [www.Pridedallas.com](http://www.Pridedallas.com). The patient needs 10-20 minutes for the test and the researcher 10 minutes to score.

Investigated population:

Many studies in different countries in patients with chronic pain and different MUS symptoms.

Results:

A cut-off point of 40 is reliable enough to differentiate between healthy controls and persons with CS or central sensitivity syndromes (CSS); patients with more severe symptoms have higher scores; the scores on the CSI decrease during treatment. Five severity levels have been developed to assess clinical interpretation of the CSI: subclinical 0-29, mild 30-39, moderate 40-49, severe 50-59, extreme 60-100).

Diagnostic test performance: sensitivity 81-95%, specificity 75-90%.

| Central sensitization inventory | Score (+,-,?) | Motivation |
| --- | --- | --- |
| Technical feasibility |  |  |
| Added value test |  |  |
| Overall judgment: suitable for use in general practice? YES/NO | | |

Thank you for your participation!

Do you know of any additional measurement instruments we have missed?

Please list these below.

We will discuss these and when appropriate we will add them in the next round of the Delphi procedure.

| Suggestions for additional measurement instruments |
| --- |
|  |
|  |
|  |
|  |

| General remarks |
| --- |
|  |
