# Supplementary material for: Tests for central sensitization in general practice: a Delphi study
Source: BMC Fam Pract. 2021 Oct 19;22:206. doi: 10.1186/s12875-021-01539-0 (PMC8527602; doi:10.1186/s12875-021-01539-0)
Supplement: Supplementary file 2 — Additional file 2: Appendix 2. Appendix first round [file 12875_2021_1539_MOESM2_ESM.docx]

Appendix 2: appendix first round

Tests for central sensitization in general practice: a Delphi **study**

Carine den Boer, MD^1^

Berend Terluin MD, PhD^1^
Johannes C. van der Wouden PhD^1^
Annette H. Blankenstein MD, PhD^1^
Henriëtte E. van der Horst MD, PhD^1^

1. Amsterdam UMC, location VUmc, Department of General Practice, Amsterdam Public Health research institute, the Netherlands.

Correspondence:

C. den Boer

Amsterdam UMC, location VUmc

Department of General Practice

Amsterdam Public Health research institute

Van der Boechorststraat 7

1081 BT Amsterdam

The Netherlands

Telephone: +31613693267

Email: [c.denboer@amsterdamumc.nl](mailto:c.denboer@amsterdamumc.nl)

Appendix Delphi procedure on measurement instruments for central sensitization

Table of contents

Introduction

**The Delphi procedure
Categories on the score form explained**

**Quantitative sensory testing (QST)**

**Conditioned pain modulation (CPM)**

Quantitative sensory tests (QST)

1. Vibratory/vibrotactile stimulus: the electric toothbrush test
2. Thermal stimulus: the painful heat or cold stimuli test

3. Tactile stimulus: pressure pain thresholds (PPT) and pressure tolerance thresholds
4. Electrical stimulus: the electrical pain and reflex thresholds

Conditioned pain modulation (CPM)

5. CPM: combination of ischemic stimuli and PPT
6. CPM: combination of PPT and cold pain
7. CPM: combination of heat and cold stimuli

Reflexes

8. The nociceptive flexion reflex (NFR)
9. The cutaneous silent period (CSP)

Measurement of cytokine levels
10. Cytokine levels in blood samples

Measurement of neurotrophin levels
11. Neurotrophin levels in blood samples

Questionnaire
12. The central sensitization inventory (CSI)

**Appendix 1: List of participants**

**Appendix 2: Table of measurement instruments from systematic review**

**Search strategy**

**References**

Introduction

Central sensitization (CS) is a mechanism explaining the cause and persistence of symptoms in absence of a specific somatic or psychiatric disease. Various definitions of CS have been proposed. In 1983 Clifford Woolf stated that chronic pain has both a peripheral sensitization component and a central sensitization component with changes in spinal cord activity [1]. This statement resulted in various theories of mechanisms for CS. Different developments such as quantitative sensory testing (QST), neurotransmitter measurement in blood samples and (functional) magnetic resonance imaging ((f)MRI) have been used to acknowledge and measure CS [2, 3].

The International Association for the Study of Pain (IASP) defined CS in 2011: “[CS is] an increased responsiveness of nociceptive neurons in the central nervous system to their normal or sub-threshold afferent input” [4]. Since then authors are using this definition in their articles but also discuss it. Hansson for example expressed his doubts about this definition because it might be too broad [5].

In 2011 Clifford Woolf wrote an article on the implications of CS for the diagnosis and treatment of pain; he concluded that it remains unclear what triggers and sustains CS, and what are the risk factors in individuals for CS [6]. He considered further research necessary to clarify these issues.

CS has been studied in relation to medically unexplained symptoms (MUS) e.g. fibromyalgia (FM), chronic fatigue syndrome (CFS), irritable bowel syndrome (IBS) and chronic pain (pain longer than 3 months) [7-9].

It is important to mention that there is no gold standard for measurement of CS. Many studies imply that specific symptoms and syndromes are linked to central sensitization, or conclude that certain quantitative sensory tests imply CS ; however, some QST can also measure peripheral sensitization.

In conclusion, CS might be the neurophysiological mechanism explaining the abovementioned symptoms. Tests measuring CS have been collected in our systematic review [10]. Until now these tests are not being used in general practice and there are no clear guidelines for the application of these measurement instruments.

To reach consensus on which measurement instruments for CS from our systematic review could be useful in general practice, we are conducting a Delphi procedure to obtain expert opinion. This procedure will consist of two e-mail rounds and possibly a teleconference at the end among doctors and specialists with experience in the field of chronic pain and/or medically unexplained symptoms.

The Delphi procedure

Delphi procedures are conducted when there is contradictory evidence or no scientific evidence on an issue [11-13]. This consensus method is frequently used in developing guidelines and consists of experts rating to what extent they agree with an issue. Disagreement can be resolved in a structured way, in rounds with controlled and repeated feedback, leading to consensus. The threshold for consensus can differ, we have chosen for 70%. Each participant is asked to vote and motivate his or her rating anonymously, this makes the Delphi procedure reliable as the influence of others is ‘neutralized’. The results after the first round are assessed and summarized and used in the next round as additional feedback. Figure 1 provides an overview of our Delphi-procedure.

Figure 1: Delphi procedure


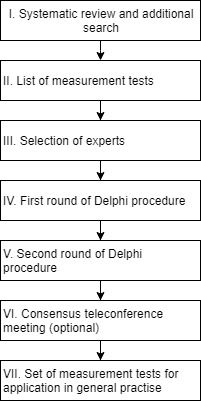


METHODS

Sample of participants

We have compiled a list of potential participants consisting of GPs and other (medical) specialists who have expertise in the domain of MUS and/or chronic pain treatment and research, from the Netherlands as well as from abroad. They received an e-mail with an invitation to participate in this Delphi procedure. Information was provided on the aim of this Delphi-procedure, a summary of the conclusions of our review on definitions, operationalizations and measurement instruments for CS, and an estimation of the time needed to rate the instruments. We offered a modest credit voucher for the effort. A list of participants that agreed on participating is attached in appendix 1.

Selection of measurement instruments

We used the list of the measurement instruments we retrieved from the literature for our systematic review (appendix 2). These comprise various measurement instruments, mostly physical tests, but also questionnaires. We excluded the tests that are inaccessible or too costly for general practice (e.g., brain MRI, fMRI, PET, somatosensory evoked potentials, sensory hypersensitivity scale).

The measurement instruments from our review are divided into categories: quantitative sensory testing (QST), conditioned pain modulation (CPM), measurement of cytokine levels, measurement of neurotrophin levels and questionnaires. We have collected as much relevant information on abovementioned properties for each category, in order to properly rate the measurement instruments. In addition to the articles from the systematic review, we conducted a search in the PubMed database with search terms covering the name of the measurement instrument in relation to central sensitization (appendix 3).

First round

Selected panellists in the Delphi procedure received a list of instruments with the following information:

For each measurement instrument in the survey:

1. Background
2. Method
3. Investigated population
4. Results
5. Scoretable: the panellists will be asked to rate (with a +/-/?) on two different aspects:

1. technical feasibility; 2. added value of the test, and to motivate their rating. Overall judgement on suitability for use in general practice.

In the appendix this information is extended with:

1. Abovementioned information for each study separately

2. Materials needed for measurement

3. Availability of materials needed for measurement

4. Burden for patient;

5. Time needed to apply the test

6. Ability of an assistant or practice nurse to perform the test

7. Reference list.

In case of insufficient expertise with regard to a particular test, the panellist is allowed to refrain from answering. However, we ask the participants to motivate why they can’t provide an answer. Panellists have the opportunity to motivate their rating.

Participants will be asked to complete and return the score form within two weeks by e-mail. When participants want to add tests they think might be relevant, they can add them. These will be included in the next round after assessment by the project team on relevance with regard to measurement of central sensitization.

Assessment of the results

The researchers will summarize the forms returned by the participants. Consensus is reached when 70% or more of the participants (who have returned the form) agree on a test as suitable or not suitable for general practice. If more than 70% conclude that the test is not suitable, we will discard the test. If no agreement has been reached on one or more tests (less agreement than the 70% threshold) or new tests have been added, these will be added to the list for the second round of our Delphi procedure. Each of these tests will have a summary of motivation on different aspects from the panellists.

Second round

Only instruments not reaching the threshold of 70% agreement and added instruments will be included in the second round to the participants. All these instruments will be presented with information on the percentage of agreement per item in the first round, a summary of participant comments and an overview of the participant’s own scores compared to group scores. In this round participants can change their rating of an instrument or motivate their decision again, both in view of the group’s scores. Every participant will be asked to complete a final score and return it via e-mail within two weeks.

Assessment of the results

The researchers will take one week to summarize the results. Similar to after round 1, also in this round instruments will only be included if they reach at least 70% agreement. Tests on which less than 70% agreement has been reached, will be put on a new list with motivations per test. This list will be used when necessary for round 3, which is an organized teleconference with the group of experts to discuss the disagreements. As in round 1 and 2, the threshold for final inclusion of the test is at least 70% consensus of the participants on the test for being suitable in general practice.

Results of the Delphi procedure

The measurement instruments of round 1, 2 and 3 that have been rated by at least 70% of the participants as suitable, will be used in the next phase. Using these results we will develop a diagnostic protocol of measurement instruments to be tested in general practice by general practitioners or their staff.

Explanation of categories score table [12]

Technical feasibility:

How would you rate the feasibility of the test in general practise? Examples of tests not being feasible in general practice are tests requiring too much time, too specific and specialised knowledge or rarely available apparatus. It is obvious that you can have doubts about the technical feasibility.

Added diagnostic value:

What is the added diagnostic value of a test? Does the result of the test enhance the probability of the disease being present of absent, combined with other available information? If a patient already has all the symptoms of a specific MUS syndrome, does this test give any added diagnostic value?

Quantitative sensory testing (QST)

QST describes testing of skin, mucosa or muscle tissue to assess pain or sensory perception pathways. As the name describes, there is a quantification of the patient’s response to types of stimuli (standardized or different types). This can be influenced by the sensitivity of patient’s sensory system or their frame of reference for pain.

The stimulus is usually steadily increasing, and the patient is instructed to notify whenever the sensation in question is for example becoming from comfortable or just pressure to pain. Tolerance can also be used as focus, measuring whenever a stimulus is becoming unbearable for the patient. A(n) (electrical) visual analogue scale (e-VAS) is usually used for assessing the level of sensation or pain.

There are different types of QST: thermal- (cold or heat), vibration- (with different frequencies), pressure- (mechanical) and electrical stimuli. Many studies combine these types of QST. These types will be explained in our measurement instrument list below.

Conditioned pain modulation (CPM)

Research over the last few decades provided evidence indicating that pain may be dependent of various endogenous pain-inhibitory processes. These are responsible for the experience of pain and operate at different levels of the central nervous system (CNS). Research suggests that (pathological) variations in endogenous pain inhibition could be responsible for the persistence or development of pain.

Investigating the differences of pain inhibition in individuals could be done with the use of CPM. This phenomenon, used to be termed as “Diffuse noxious inhibitory control (DNIC)”, refers to a psychophysical process. A noxious stimulus (the conditioned stimulus) reduces or inhibits the perception of another ‘secondary’ noxious stimulus (the test stimulus) applied to a specific area of the body (dependent of the study). This test surveys the net inhibitory and excitatory effect of nociceptive pathways on sensitivity of pain in a specific body part other than the part of the body being conditioned (with a conditioning stimulus). Reduction of pain perception of the test-stimulus or increase of pain threshold induced by the test-stimulus after receiving a conditioning stimulus is considered as an inhibitory CPM effect. In central sensitization a reduction of inhibition is found due to reduced inhibition of descending control.

1. Vibratory/vibrotactile stimulus: The electric toothbrush test

Background

Producing vibrotactile stimuli by an electric toothbrush can cause a (punctuate and dynamic) mechanical and thermal stimulation with temporal summation (a central nerve system condition with an increased perception to repetitive stimuli related to central sensitization, pain in this case [2]).

Study 1
Method: an electric toothbrush will perform a vibrotactile stimulus with 1 pound pressure for 30 seconds in four different areas. These areas are on the masseter, temporomandibular joint, temporalis and mid-ventral spot of the forearm. At 0, 15, 30 and 60 seconds a pain intensity will be recorded from a 0 to 10 scale.

Investigated population: 14 females with temporomandibular disorders (TMD) with myofascial pain and arthralgia.

Results: Sensitivity of 57% over all 4 areas, specificity of 92% over all 4 areas.

Study 2
Method: see study 1

Investigated population: 16 painful TMD (women), 29 normal control without TMD/chronic orofascial pain (women)

Results: Patients with TMD had higher pain sensitivity and lower pressure point threshold values in trigeminal region (p<0.01) and same results for areas outside the trigeminal region (p<0.01).

For all studies:

Materials needed for measurement: Electric toothbrush, circa 40 euro

Availability of materials needed for measurement: accessible for general practise

Burden on patient: low burden on patient

Time needed to apply the test: 8 minutes (less than 2 minutes per stimulus area)

Ability of an assistant or practice nurse to perform the test: Yes

References
Study 1: D.R. Nixdorf, A. Hemmaty, J.O. Look, E.L. Schiffman, M.T. John, Electric toothbrush application is a reliable and valid test for differentiating temporomandibular disorders pain patients from controls, BMC Musculoskelet Disord 10 (2009) 94.

Study 2: L.B. Campi, P.C. Jordani, H.L. Tenan, C.M. Camparis, D.A. Goncalves, Painful temporomandibular disorders and central sensitization: implications for management-a pilot study, International journal of oral and maxillofacial surgery 46(1) (2017) 104-110.

1. The painful heat or cold stimuli test

Background

Central sensitization is characterised by generalized (widespread) hypersensitivity and enhanced temporal summation of pain. Repetitive painful heat stimuli may induce temporal summation by an enhanced response in dorsal horn neurons by repetitive stimulation of C-fibers. This results in an increased perception of pain with a constant or reduced peripheral input. This is an example of enhanced central excitability, referring to central sensitization.

Study 1
Method: participants received 6 heat stimuli on the hands and shoulders while sitting comfortably. The heat probe was brought on the skin for 10 seconds, from 38 degrees to 44/46. There was an interval of at least 1 minute or until painful aftersensations disappeared between each stimulus. During this test participants used an electronic visual analog scale (e-VAS) for rating their pain intensity.

Investigated population: 36 FM patients (as chronic widespread pain), 24 local musculoskeletal pain patients (LMP), 23 control participants

Results:
Shoulder: Post hoc rating showed significant difference only between control and FM patients (p<0.02), other group comparisons were not different (p>0.05).
Hands: Post hoc testing showed higher significant pain ratings in FM than other groups, also LMP subjects had significantly higher pain ratings compared to control group.

Study 2
Method: participants received the heat and cold stimuli in the inside of the forearm using a thermode (from 0 to 50 degrees). This change in temperature was linear and 1 degree/second, starting from the baseline set on 32 degrees. Perception and pain thresholds were assessed.

Investigated population: 85 FM patients, 40 control participants

Results: Thresholds for cold and heat sensation were similar for FM patients and control participants. FM patients had significantly lower cold and heat pain thresholds (p<0.001 and p=0.005). Cold tolerance was much lower (by 66%) in patients with FM compared to control participants (p<0.001).

For all studies:

Materials needed for measurement: thermal sensory analyser (circa 110 euro for a basic thermal monitoring analyser), Peltier thermode (circa 10 euro)

Availability of materials needed for measurement: accessible for primary care

Burden on patient: low to medium burden on patient, mean VAS 3, max VAS 4,7 (on a scale of 10)

Time needed to apply the test: <10 minutes

Ability of an assistant or practice nurse to perform the test: yes

References

Study 1: R. Staud, E.E. Weyl, D.D. Price, M.E. Robinson, Mechanical and heat hyperalgesia highly predict clinical pain intensity in patients with chronic musculoskeletal pain syndromes, J Pain 13(8) (2012) 725-35.

Study 2: J.A. Desmeules, C. Cedraschi, E. Rapiti, E. Baumgartner, A. Finckh, P. Cohen, P. Dayer, T.L. Vischer, Neurophysiologic evidence for a central sensitization in patients with fibromyalgia, Arthritis and rheumatism 48(5) (2003) 1420-9.

3. Pressure pain thresholds (PPTs) and pressure tolerance thresholds

Background

Most QST studies use pressure pain thresholds (PPT) for measuring central sensitization. In addition there are tests which measure increased perceived pain intensity to slowly repeated evoked pain stimuli (SREP). Next studies evaluate pressure pain thresholds (PPT) and thresholds for tolerance in patients with FM, chronic low back pain and neck pain by using an e-VAS and pain sensitization in FM patients in response to SREP.

Study 1

Method
The intensity of subjective evoked pain is assessed (using a VAS) by applying pressure stimulation on the fingernail with a wireless pressure algometer. The marker of central sensitization is slowly repeated evoked pain (SREP). 9 pain stimuli are given with an unknown timing and duration of the SREP stimuli, with each pain stimulus lasting for 5 seconds with a constant pressure.

Investigated population
24 women with FM and 24 healty participants as control group

Results

Sensitivity of 79% for SREP
Specificity of 92% for SREP

Perception of pain intensity during the SREP stimuli increased in the patients with fibromyalgia and did not increase for the healthy participants.

Study 2

Method: pain pressure threshold and tolerance thresholds were measured using an electronic pressure algometer. Pressure started at 0kPa and increased to a pressure of maximum 1000kPa (30kPa/sec). These stimuli were applied in the suprascapular region, second toe, neck (with most severe pain, cases only), a non-painful site caudal to painful area of neck (5 cm caudal, cases only) and lower back.

Investigated population: 40 chronic neck pain patients and 300 control subjects

Results
Crude AUC: 1). Detection: site of the most severe pain at neck 0.93 (0.87–1.00). 2) Tolerance: site of the most severe pain at neck 0.87 (0.78–0.95). Fitted AUC: 1) Detection: site of the most severe pain at neck 0.94 (0.88–1.00) and 2) Tolerance: site of the most severe pain at neck 0.87 (0.78–0.97).

Results:
Pressure stimulation on the most severe pain sites is the most appropriate QST for distinguishing patients with chronic neck pain from asymptomatic control subjects.

Study 3

Method: pain pressure threshold and tolerance thresholds were measured using an electronic pressure algometer. Pressure started at 0kPa and increased to a pressure of maximum 1000kPa. These stimuli were applied in the suprascapular region, second toe, lower back (with most severe pain, cases only), a non-painful site cranial to painful area of lower back (5 cm cranial, cases only) and lower back in absence of painful area (for the controls).

Investigated population: 40 chronic low back pain patients and 300 control subjects

Results:

The best ranked tests with ROC curve >0.80 were the following:
- pressure pain threshold of suprascapular AUC (95% CI) of 0.80 (0.71-0.89) and sites of most severe pain in lower back AUC 0.87 (0.81-0.94)
- Tolerance threshold of most severe pain in lower back AUC (95%) 0.80 (0.71-0.89)

Pressure pain thresholds as measure of pain hypersensitivity had the highest ability in distinguishing chronic low back pain from control subjects.

For all studies:

Materials needed for measurement: (non)electronic pressure algometer (not electronic circa 200 euro)

Availability of materials needed for measurement: accessible for primary care

Burden on patient: medium burden on patient, patients were instructed to press a button once the stimulus became too uncomfortable

Time needed to apply the test: average of 5 to 10 minutes

Ability of an assistant or practice nurse to perform the test: yes

References

Study 1: P. de la Coba, S. Bruehl, M. Moreno-Padilla, G.A. Reyes Del Paso, Responses to Slowly Repeated Evoked Pain Stimuli in Fibromyalgia Patients: Evidence of Enhanced Pain Sensitization, Pain medicine (Malden, Mass.) 18(9) (2017) 1778-1786.

Study 2: A.Y. Neziri, A. Limacher, P. Juni, B.P. Radanov, O.K. Andersen, L. Arendt-Nielsen, M. Curatolo, Ranking of tests for pain hypersensitivity according to their discriminative ability in chronic neck pain, Regional anesthesia and pain medicine 38(4) (2013) 308-20.

Study 3: A.Y. Neziri, M. Curatolo, A. Limacher, E. Nuesch, B. Radanov, O.K. Andersen, L. Arendt-Nielsen, P. Juni, Ranking of parameters of pain hypersensitivity according to their discriminative ability in chronic low back pain, Pain 153(10) (2012) 2083-91.

4. The electrical pain and reflex thresholds

Background

Spontaneous widespread pain, lower pain threshold and mechanical allodynia (innocuous stimuli such as soft touch are perceived as painful) are common in FM and chronic whiplash-associated disorders (WAD) and are suggested to have a link with central sensitization (CS). The aim of following studies is to determine whether abnormalities exist in peripheral and central nociceptive sensory input processing located outside the areas of spontaneous pain. This is an indicator for the presence of widespread hyperalgesia (clinical manifestation of central sensitisation). This is done in patients with FM and patients with WAD using nociceptive withdrawal reflex (NWR)/nociceptive flexion R-III reflex (NFR) (withdrawal reflex after damaging stimuli), pain thresholds and referred pain after electrical stimulation.

Study 1

Method: systematic review
- Lemming et al. (2005): Intramuscular and cutaneous pain thresholds were measured after repeated electrical stimulation compared to a single electrical stimulation in the lower limbs of patients with chronic WAD.

- Kosek and Januszewska (2008): Intramuscular electrical stimulation was used to induce referral pain in patients with WAD.

Investigated population: for all studies: patients with chronic WAD compared to control participants

Results:
- Lemming et al. (2005): Patients with chronic WAD had a lower intramuscular and cutaneous pain threshold after repeated electrical stimulation compared to single electrical stimulation in their lower limbs. This supporting the theory of pain hypersensitivity occurring on healthy areas, is a sign of hyperexcitability of the central nervous system.
- Kosek and Januszweska (2008): Patients with chronic WAD had a higher sensitivity to intramuscular stimulation and needed a lower intensity of conditioning stimulation to induce referred pain compared to the healthy control group. Chronic WAD patients had a larger spread of areas in referral pain. This abnormal spread of referral pain provided evidence that patients with chronic WAD had an altered central nervous system processing nociceptive input.

Materials needed for measurement: EMG (e.g. Medelec EMG device, costs between 750 and 2000 euro)

Availability of materials needed for measurement: accessible for primary care

Burden on patient: low to medium burden on patient, an electrical stimulus can give an uncomfortable sensation.

Time needed to apply the test: average of 10 minutes

Ability of an assistant or practice nurse to perform the test: yes

References
Study 1: J. Van Oosterwijck, J. Nijs, M. Meeus, L. Paul, Evidence for central sensitization in chronic whiplash: a systematic literature review, European journal of pain (London, England) 17(3) (2013) 299-312.

1. CPM: combination of ischemic stimuli and PPT

Background:

Conditioned pain modulation (CPM) means that a test-stimulus and a conditioning stimulus are used together in the test. A combination of ischemic stimuli and PPTs is used here as CPM. In healthy controls the conditioning stimulus leads to a reduction in the perceived intensity of the test-stimulus. In central sensitization there is a smaller reduction of the perceived intensity of the test-stimulus due to reduced inhibition of descending control.

Study 1
Method: in comfortable recumbent position CPM is evaluated by conditioning tonic stimulation of pain using ischemic compression (with a tourniquet cuff) on the left arm until pain was rated as 4cm on e-VAS and PPT from peripatellar region.

Investigated population: 17 knee osteoarthritis patients

Results multiple-regression model: 55% of variance in peak pain intensity in KOA patients (p=0.001). Significant correlations (P < 0.05): PPTs by handheld pressure algometry in peripatellar region vs. TA (R = 0.94), PPTs by computer controlled pressure algometry vs. handheld pressure algometry in peripatellar region (R = 0.71), PPTs by computer-controlled pressure algometry in peripatellar region vs. handheld pressure algometry on TA (R = 0.71) and temporal summation at the knee vs. TA (R = 0.73).

Study 2
Method: CPM was induced by inflating an occlusion cuff at the subject’s left arm to a painful intensity. The occlusion cuff was inflated at a rate of 20 mmHg/s until ‘the first sensation of pain’ and maintained for 30 s. Afterwards, pain intensity, as a result of cuff inflation, was rated on a numerical rating scale (0 = no to 10 = worst possible pain). Next, cuff inflation was increased or decreased until pain intensity at left arm was rated as 3/10. TS assessment was then repeated during maintenance of the cuff inflation.

TS was provoked by means of 10 consecutive pulses at previously determined pressure pain threshold at each location. TS started 2 min after pressure pain threshold measurement. For each pulse, pressure was gradually increased at a rate of 2 kg/s to the determined pressure pain threshold and maintained for 1 s before being released (1 s interstimulus interval). Pain intensity of first, fifth, and 10th pulse was rated on a numerical rating scale (0 = no to 10 = worst possible pain). Afterwards, a rest period of 5 min was allowed.

Investigated population: 35 chronic patients with whiplash associated disorder (WAD), 31 healthy controls

Results: TS of pressure pain was significantly depleted among healthy controls. In contrast, TS was quite similar prior to and during cuff inflation in chronic WAD, providing evidence for dysfunctional CPM in patients with chronic WAD. The present study demonstrates a lack of endogenous pain inhibitory pathways, and in particularly CPM, in patients with chronic WAD, and hence provides additional evidence for the presence of central sensitization in chronic WAD.

Study 3
Method: pressure pain modulation by heterotopic descending noxious inhibitory control (DNIC), ischemic compression of the arm. A 7.5cm wide tourniquet cuff (VBM, Germany) was wrapped around the left arm. The lower rim of the tourniquet cuff was at 3cm proximal to the cubital fossa. The cuff control unit (Aalborg University, Denmark) was programmed to maintain the pressure at 36kPa (above the systolic pressure). After the target pressure was reached, the patient was asked to repeat hand grip for 10 times or more until 4 on the VAS was reached. The patients rated the contraction-evoked pain on an electronic VAS on which “0” represented “no pain” and “10” represented “maximal pain”. When “4” on VAS was reached, PPTs on all test sites and control sites were assessed. The cuff was released once PPT assessments were finished. PPT assessment was repeated 5min after cuff-evoked pain subsided.

Investigated population: 48 pts knee OA, 2 groups 24 VAS >6 ,24<6, 24 controls

Results: There were no significant increases in PPT at the peripatellar region during cuff stimulation in neither group A nor group B. PPT increased significantly during cuff stimulation in controls (ANOVA: F_2,146_=6.1, p<0.01; SNK: p<0.05). Significant increase of PPT during cuff stimulation was found at TA in groups A, B and controls and at forearm in group A and controls (ANOVA: F_2,146_=5.6, p<0.01; SNK: p<0.05;). Patients with osteo-arthrosis had a significant facilitation of temporal summation from both the knee and tibialis anterior, significantly less DNIC as compared with controls.

References:

Study 1: S.T. Skou, T. Graven-Nielsen, L. Lengsoe, O. Simonsen, M.B. Laursen, L. Arendt-Nielsen, Relating clinical measures of pain with experimentally assessed pain mechanisms in patients with knee osteoarthritis, Scandinavian journal of pain 4(2) (2013) 111-117.

Study 2: L. Daenen, J. Nijs, N. Roussel, K. Wouters, M. Van Loo, P. Cras, Dysfunctional pain inhibition in patients with chronic whiplash-associated disorders: An experimental study, Clinical rheumatology 32(1) (2013) 23-31.

Study 3: L. Arendt-Nielsen, H. Nie, M.B. Laursen, B.S. Laursen, P. Madeleine, O.H. Simonsen, T. Graven-Nielsen, Sensitization in patients with painful knee osteoarthritis, Pain 149(3) (2010) 573-81.

1. CPM: combination of PPT and cold pain

Background:

Conditioned pain modulation (CPM) means that a test-stimulus and a conditioning stimulus are used together in the test. A combination of cold pain stimulation and PPTs is used here as CPM. In healthy controls the conditioning stimulus leads to a reduction in the perceived intensity of the test-stimulus. In central sensitization there is a smaller reduction of the perceived intensity of the test-stimulus due to reduced inhibition of descending control.

Study 1
Method: Patients were instructed to immerse their foot in a bucket of ice water on the same side as the lumbar region with the most pain. This was the conditioning stimulus. Inducing the pain on the lumbar region was done by a cold pressor test triggering the CPM response and PPTs were measured in that region and the tibialis anterior (TA) after immersion of the foot in the cold water.

Investigated population: 30 patients with chronic non-specific low back pain (CLBP) compared to 30 healthy subjects

Results: patients with CLBP had significant lower PPT than controls in lumbar region (89.5kPa as mean difference 95% CI 40.9-131.1 kPa) and in the TA (59.45 kPa (mean difference) 95% CI 13.49-105.42 kPa). CPM: patients with CLBP had a significant decrease in PPT in lumbar regio (mean = −47.17 kPa, SD = 73.3) after cold pressor test compared to control group. The control group had an increase in PPT (mean = 71.4, SD = 83.8). The change in PPT between both groups was significant (between-group difference = 118.6 kPa, 95% CI 77.9-159.2 kPa; p<0.001). In men no significant difference has been found in PPTs.

The findings show a significant difference in PPT between CLBP patients and controls only in women. This suggesting that the difference is only driven by changes in women, not in men. Indicating an enhanced widespread sensitivity in women.

Study 2

Method: Patients received an ascending measure of PPT inducing a (verbal) pain score of 4 out of 10. This was done on the anterior shin of the affected knee. A conditioned stimulus was applied in the form of cold stimulus (Medoc TSAII Neurosensory Analyzer) inducing a (verbal) pain score of 6 out of 10. This was applied on the opposite forearm for one minute. PPT was evaluated from before the conditioning stimulus and after.

Investigated population: One hundred thirty-three participants with knee osteoarthritis (KOA), 56.4% female

Results: Negative values indicated pain inhibition and positive values did the opposite, indicating pain facilitation. Patients with positive values were suggested to be linked with CS.

For all studies:

Materials needed for measurement depends on combination of tests:

PPT: handheld or electronic pressure algometer (handheld circa 200 euro), cold stimulation: bucket of ice water, thermometer and Medoc TSAII Neurosensory Analyzer (circa 4700€)

Availability of materials needed for measurement: Yes

Burden on patient: Medium

Time needed to apply the test: 10-15 minutes
Ability of an assistant or practice nurse to perform the test: Yes

References:

Study 1: J.B. Correa, L.O. Costa, N.T. de Oliveira, K.A. Sluka, R.E. Liebano, Central sensitization and changes in conditioned pain modulation in people with chronic nonspecific low back pain: a case-control study, Exp Brain Res 233(8) (2015) 2391-9.

Study 2: J. Gervais-Hupe, J. Pollice, J. Sadi, L.C. Carlesso, Validity of the central sensitization inventory with measures of sensitization in people with knee osteoarthritis, Clinical rheumatology 37(11) (2018) 3125-3132.

1. CPM: combination of heat and cold stimuli

Background:

Conditioned pain modulation (CPM) means that a test-stimulus and a conditioning stimulus are used together in the test. A combination of heat stimulation and cold stimulation is used here as CPM. In healthy controls the conditioning stimulus leads to a reduction in the perceived intensity of the test-stimulus. In central sensitization there is a smaller reduction of the perceived intensity of the test-stimulus due to reduced inhibition of descending control.

Study 1
Method: Participants received a phasic heat test stimulus with a peak temperature of 50 degrees Celsius on their left palm. Their right hand was submerged in painful water of 12 degrees Celsius, this was the counter irritation. The diffuse noxious inhibitory control (DNIC) effect (same as CPM effect) measured reduction in average pain rating (APR) during this procedure, compared with baseline. This was compared in the patients with IBS and the controls.

Investigated population: 48 premenopausal females, 27 with IBS

Results: during this procedure patients with IBS had smaller DNIC than controls (p=0.011, after repeating measures of analysing variance) and greater depression, anger-out, symptoms of catastrophizing and state-anxiety expression (p<0.05). CPM had enhanced group differences for psychological measures and after controlling for non-specific effects during non-painful CS (p=0.001, repeated measures of analysing the covariance). These results demonstrate a deficient DNIC/CPM in patients with IBS.

Study 2
Method: Patients were instructed to immerse their non-dominant hand in cold water (0-1 degrees Celsius) for 1 minute. This stimulus is used as test stimulus to elicit CPM. The conditioning stimulus was done by thermal pain using thermal pain thresholds (TPTs) on the dominant forearm on a pain score of 6/10 during the last 30 seconds of cold-water immersion. The CPM was determined by subtracting the pain scores during this TPT testing.
Investigated population: 77 patients with chronic musculoskeletal pain and 63 healthy controls
Results: CPM served as a marker with effect indicating impairment of descending pain-modulatory system in patients with long-term pain condition.

For all studies:

Materials needed for measurement depends on combination of tests:

Thermal stimuli: thermal sensory analyser (circa 110 euro for a basic thermal monitoring analyser),

Cold pressor test: bucket with cold water (12 degrees Celsius), thermometer

Availability of materials needed for measurement: Yes

Burden on patient: low to medium burden on patients

Time needed to apply the test: 15 minutes
Ability of an assistant or practice nurse to perform the test: yes

References:

Study 1: J.S. Heymen, Central processing of noxious stimuli in patients with irritable bowel syndrome compared to healthy controls, ProQuest Information & Learning, US, 2007, pp. 1976-1976.

Study 2: W. Caumo, L.C. Antunes, J.L. Elkfury, E.G. Herbstrith, R. Busanello Sipmann, A. Souza, I.L. Torres, V. Souza Dos Santos, R. Neblett, The Central Sensitization Inventory validated and adapted for a Brazilian population: psychometric properties and its relationship with brain-derived neurotrophic factor, Journal of pain research 10 (2017) 2109-2122.

8. The nociceptive flexion reflex (NFR)

Background:

Upregulation of defensive reflexes such as the nociceptive flexion reflex (NFR) has been attributed to sensitisation of peripheral and spinal nociceptors and is often considered biomarkers of pain. Experimental modulation of defensive reflexes raises the possibility that they might be better conceptualised as markers of descending cognitive control. The pattern of difference is more consistent with top-down cognitive control reflective of heightened protection of body tissue. The pattern of modulation is dependent on potentially complex evaluative mechanisms. Defensive reflex threshold may reflect a biomarker of a broader psychological construct related to bodily protection, rather than sensitisation of primary nociceptors, spinal nociceptors, or pain.

The NFR is obtained after electrical stimulation applied directly to the sural nerve, circumventing peripheral nociceptors and directly stimulating the nociceptive pain pathway.

An EMG device delivers small electrical pulses on the skin. Patients rest comfortably in a supine position in order to obtain muscular relaxation. Cutaneous electrodes are applied, and the sural nerve is stimulated in its retromaleolar track. The electrical stimulus consists of single rectangular impulses (0.5 msec) delivered with 6–10 second interstimulus interval, by a constant current stimulator at variable intensities (1–100 mA) (Nicolet Viking IV; Nicolet, Madison, WI). Electromyographic responses are recorded using a pair of surface electrodes placed over the tendon of the ipsilateral biceps femoris. The R‐III reflex (objective threshold) is identified as a multiphasic signal appearing at least 90 msec but less than 250 msec after each stimulation and is considered to be present when the corrected computed surface is >0.5 mV/msec (positive response).

Study 1
Method: Systematic review with meta-analysis

Investigated population: 17 studies, chronic pain group 1188, control group 1035

Results A sensitivity analysis, including only those studies with a ‘low risk’ of bias, resulted in an increase in effect size [− 0.9 (95% CI, − 1.27 to − 0.52), p < 0.0001] and little influence on the heterogeneity between studies (I2 = 83%). Pain groups had an overall lower reflex threshold than controls, with a large effect size [− 0.83 (95% CI, −1.18 to −0.47), p < 0.0001].

Study 2
Method: An electrical stimulation is applied at variable intensities (1-100mA) on the sural nerve with interstimulus interval of 6-10 seconds. This will obtain a nociceptive flexion R-III reflex (NFR). The electromyographic responses were recorded by using surface electrodes placed on the tendon of the ipsilateral biceps femoris. The R-III reflex was considered to be a positive response when the corrected computed surface was larger than 0.5mV/msec. Following the electrical stimulation, patients were asked to describe their sensation using three scales (numerical rating scale, sensitive scale with 7 categories and affective scale with 7 categories). The person is rested in a supine position to obtain muscular relaxation.

Investigated population: 85 outpatients with FM attending a self‐management program and 40 non-FM controls

Results:
Sensitivity of 73,1% for NFR with a cutoff value of <27,6mA
Specificity of 80,4% for NFR with a cutoff value of <27,6mA

The NFR threshold in patients with FM was 33% lower compared to the control group.

For all studies:

Materials needed for measurement: EMG (e.g. Medelec EMG device, costs between 750 and 2000 euro)

Availability of materials needed for measurement: available

Burden on patient: Low to medium burden on patients (max 4,7 (mean) VAS for patients with FM).

Time needed to apply the test: 15 minutes
Ability of an assistant or practice nurse to perform the test: training necessary

References:

Study 1: S.B. Wallwork, L. Grabherr, N.E. O'Connell, M.J. Catley, G.L. Moseley, Defensive reflexes in people with pain - a biomarker of the need to protect? A meta-analytical systematic review, Reviews in the neurosciences 28(4) (2017) 381-396

Study 2: J.A. Desmeules, C. Cedraschi, E. Rapiti, E. Baumgartner, A. Finckh, P. Cohen, P. Dayer, T.L. Vischer, Neurophysiologic evidence for a central sensitization in patients with fibromyalgia, Arthritis and rheumatism 48(5) (2003) 1420-9

Study 3: V. Skljarevski, N.M. Ramadan, The nociceptive flexion reflex in humans -- review article, Pain 96(1-2) (2002) 3-8

9. The cutaneous silent period

Background:

The NFR and cutaneous silent period are excitatory and inhibitory parts of the same spinal protective reflex, respectively. The CSP is a brief pause in muscle action potentials following strong stimulation of the cutaneous nerve during a sustained voluntary contraction, and is considered a protective reflex mediated by the spinal inhibitory circuit and reinforced by parallel modulation of the motor cortex.

The formation of CSP has 3 phases: (1) peripheral conduction time managed by A-delta fibers; (2) duration of the inhibition in the spinal cord; and (3) transmission time from the spinal cord to muscle motor fibers

The CSP is recorded in the right upper and left lower extremities or measured from the abductor pollicis brevis muscle muscle evoked by electrical stimulation of the right fifth digit.

During steady submaximal (50% of the maximal contraction) thumb abduction, 10 consecutive painful electrical stimuli of standard 80-mA intensity and 0.5-ms duration are applied to the index ﬁnger and responses were superimposed. The trace of the responses on the screen and EMG audio signal were taken into account to provide submaximal constant contraction during voluntary contraction.

Study 1

Method: Measurement of CSP

Investigated population: Twenty-nine patients with myofascial pain syndrome (MPS) and 30 healthy volunteers were included in the study.

Results::In both upper and lower extremities, patients had prolonged CSP latencies (P = 0.034 and P = 0.049 respectively) and shortened CSP durations (P = 0.009 and P = 0.008, respectively).

S

Study 2

Method: Measurement of CSP

Investigated population: 24 patients with FM and 24 age- and sex-matched healthy volunteers.

Results:: Mean CSP latencies did not differ between patients (55.50 ± 10.97 ms) and healthy controls (60.23 ± 11.87 ms; p = 0.158), although the mean CSP duration was significantly longer in patients (73.75 ± 15.67 ms) than in controls (63.50 ± 14.05 ms; p = 0.021).

Study 3

Method: Measurement of CSP

Investigated population: Twenty-eight patients with FM and 18 healthy volunteers

Results: The CuSP onset latencies were significantly longer in patients with FM. There was not any significant difference in CuSP duration between patients and controls.

For all studies:

Materials needed for measurement: EMG (for example Medelec EMG device, costs between 750 and 2000 euro); surface electrodes (1 euro pro person).

Availability of materials needed for measurement: sometimes available in a lab.

Burden on patient: low to medium burden on patients

Time needed to apply the test: 15 minutes
Ability of an assistant or practice nurse to perform the test: training necessary

References

Study 1: O. Kilinc, S. Sencan, T. Ercalik, P.K. Koytak, H. Alibas, O.H. Gunduz, T. Tanridag, K. Uluc, Cutaneous silent period in myofascial pain syndrome, Muscle & nerve 57(1) (2018) E24-e28.

Study 2: S.H. Baek, H.Y. Seok, Y.S. Koo, B.J. Kim, Lengthened Cutaneous Silent Period in Fibromyalgia Suggesting Central Sensitization as a Pathogenesis, PloS one 11(2) (2016) e0149248.

Study 3: O. Sahin, S. Yildiz, N. Yildiz, Cutaneous silent period in fibromyalgia, Neurological research 33(4) (2011) 339-43.

10. Cytokine levels in blood samples

Background

Cytokines are proteins with an effect on other cells on communication and interaction. There are two categories of cytokines, pro-inflammatory and anti-inflammatory cytokines. Higher serum levels of tumor necrosis factor-alpha (TNF-α) and pro-inflammatory interleukines (Il-1, IL-6, IL-8) and a reduction of anti-inflammatory interleukines (IL-4, IL-10) might lead to neuroinflammation, e.g. of the glia cells.

These changes are also associated with several components of sickness behaviour and have been found changed in chronic pain and MUS.

However, much research in this field has been carried out in animal studies. Human studies find often contradictory results and no good association between the level of the cytokines and the severity of the pain has been found.

Study 1

Method: Collecting a blood sample and measurement of IL-8, a pro-inflammatory cytokine at different moments during treatment. The blood sample has to be processed in a lab which has the equipment to measure interleukine-8.

Investigated population: 20 fibromyalgia patients and 80 healthy controls. Patients underwent a multidisciplinary therapy as inpatients 3 weeks, 5 days a week.

Results: From day 0 to day 10, IL-8 serum level reduced significantly (P<0.05) and stayed at this level constantly from day 10 to day 21. From day 21 to 6 months, there was a slight decrease of IL-8 level. At the end of the study, circulating IL-8 level was significantly lower than in the beginning of the study (P<0.05), having declined to normal range.

Study 2

Method: Collecting a blood sample and measurement of TNF-α. The blood sample has to be processed in a lab which has the equipment to measure TNF-α.

Investigated population: 23 patients with somatization syndromes (Somatoform Symptom Index-8, SSI-8), 23 age- and sex-matched healthy controls and 23 patients with major depression

Results: this study is the first to demonstrate increased levels of TNF-α and neopterin in patients with somatization syndromes without a diagnosis of depression, which may support a role of immune alterations in somatization syndromes.

Study 3

Method: Collecting a blood sample and measurement of TNF-α. The blood sample has to be processed in a lab which has the equipment to measure TNF-α.

Investigated population: 120 patients with chronic low back pain were matched to a healthy control group and followed 6 months.

Results: in the beginning and at all other time points, there was a significantly higher proportion of TNF-a positive participants in the patients group than in the control group. The proportion of TNF-a positive patients decreased during the first 10 days of a multidisciplinary therapy in the patient group, but after this initial period, TNF-a levels remained constantly high with no further change until the final follow-up.

Study 4

Method: Collecting a blood sample and measurement of basal inflammatory markers C-reactive protein, interleukin-6, and TNF-α. The blood sample has to be processed in a lab which has the equipment to measure these cytokines.

Investigated population: chronic multisite musculoskeletal pain group (n = 754) and a control group (n = 878). Blood levels of the basal inflammatory markers C-reactive protein, interleukin-6, and tumor necrosis factor-alpha were determined.

Results: subjects with chronic multisite musculoskeletal pain showed elevated levels of basal inflammatory markers compared with controls, but statistical significance was lost after adjustment for lifestyle and disease variables.

For all studies:

Materials needed for measurement: blood sample of patient, lab to process the cytokines

Availability of materials needed for measurement: sometimes available in a lab.

Burden on patient: low to medium burden on patients

Time needed to apply the test: dependent on the lab
Ability of an assistant or practice nurse to perform the test: sometimes they can take a blood sample, mostly laboratory technicians do this

References:

Study 1: H. Wang, M. Buchner, M.T. Moser, V. Daniel, M. Schiltenwolf, The role of IL-8 in patients with fibromyalgia: a prospective longitudinal study of 6 months, The Clinical journal of pain 25(1) (2009) 1-4.

Study 2: F. Euteneuer, M.J. Schwarz, A. Hennings, S. Riemer, T. Stapf, V. Selberdinger, W. Rief, Psychobiological aspects of somatization syndromes: contributions of inflammatory cytokines and neopterin, Psychiatry research 195(1-2) (2012) 60-5.

Study 3: H. Wang, M. Schiltenwolf, M. Buchner, The role of TNF-alpha in patients with chronic low back pain-a prospective comparative longitudinal study, The Clinical journal of pain 24(3) (2008) 273-8.

Study 4: E. Generaal, N. Vogelzangs, G.J. Macfarlane, R. Geenen, J.H. Smit, J. Dekker, B.W. Penninx, Basal inflammation and innate immune response in chronic multisite musculoskeletal pain, Pain 155(8) (2014) 1605-12.

11. Neurotrophin levels in blood samples

Background
Neurotrophines are proteins, regulating neuronal function and affecting the growth and survival of neurons, in the peripheral and central nervous system. As neurotrophines and their receptors are located and expressed in areas of the brain responsible for its’ plasticity, it is thought that they could regulate the synaptic plasticity. Much research has been carried out with animals and early animal data suggest that BDNF plays a maladaptive role on descending inhibitory and facilitatory pathways in the brain. In chronic (musculoskeletal) pain and overactive bladder, serum or urine levels of neurotrophines such as nerve growth factor (NGF) and brain derived neurotrophic factor (BDNF) have been found increased.

Study 1

Method: A blood sample is analyzed for BDNF levels in a lab.

Investigated population: cross-sectional study, including 56 females with confirmed FM aged 18–65 years

Results: Statistical significant correlation between BDNF level and decrease of PPTs. No reference values. BDNF was inversely correlated with the PPT.

Study 2

Method: assessesment of serum BDNF in blood samples

Investigated population: cross-sectional study pooled baseline data from three randomized clinical trials. Inclusion of females (n = 114), aged 19–65 years old with disability by chronic pain syndromes (CPS): FM (n = 19), MPS (n = 54), OA (n = 27) and healthy subjects (n = 14).

Results: The BDNF was inversely correlated with the SICI and with the change on NPS (0–10) during CPM-task. These findings suggest greater disinhibition in the motor cortex and the descending pain inhibitory system in FM and MPS than in OA and healthy subjects.

Study 3

Method: measurement of brain-derived neurotrophic factor (BDNF), tumor necrosis factor-a (TNF-a), and interleukins 6 (IL6) and IL-10 in blood samples.
Investigated population: Female patients with MUS symptoms (chronic tension-type headache [n = 30], myofascial pain syndrome [n = 29], fibromyalgia [n = 22]); with chronic pain (osteoarthritis [n = 27] and endometriosis [n = 32]); and in pain-free controls (n = 37).

Results: Patients with MUS symptoms presented higher serum TNF-a (28.61± 12.74 pg/mL) and BDNF (49.87± 31.86 ng/mL) than those with chronic pain (TNF-a= 17.35± 7.38 pg/mL; BDNF= 20.44± 8.30 ng/mL) and controls (TNF-a= 21.41± 5.74 pg/mL, BDNF= 14.09± 11.80 ng/ mL). Moreover, MUS patients presented lower IL levels. Receiver operator characteristics analysis showed the ability of BDNF to screen MUS and chronic pain from controls (cutoff= 13.31 ng/mL, area under the curve [AUC]= 0.86, sensitivity= 95.06%, specificity= 56.76%).

For all studies:

Materials needed for measurement: blood sample of patient, lab to process the neurotrophines

Availability of materials needed for measurement: sometimes available in a lab.

Burden on patient: low to medium burden on patients

Time needed to apply the test: dependent on the lab
Ability of an assistant or practice nurse to perform the test: sometimes they can take a blood sample, mostly laboratory technicians do this.

References

Study 1: S.A. Zanette, J.A. Dussan-Sarria, A. Souza, A. Deitos, I.L. Torres, W. Caumo, Higher serum S100B and BDNF levels are correlated with a lower pressure-pain threshold in fibromyalgia, Molecular pain 10 (2014) 46.

Study 2: W. Caumo, A. Deitos, S. Carvalho, J. Leite, F. Carvalho, J.A. Dussán-Sarria, M.G.L. Tarragó, A. Souza, I.L.S. Torres, F. Fregni, Motor cortex excitability and BDNF levels in chronic musculoskeletal pain according to structural pathology, Frontiers in Human Neuroscience 10(2016JULY) (2016).

Study 3: A. Deitos, J.A. Dussan-Sarria, A. Souza, L. Medeiros, G. Tarrago Mda, F. Sehn, M. Chassot, S. Zanette, A. Schwertner, F. Fregni, I.L. Torres, W. Caumo, Clinical Value of Serum Neuroplasticity Mediators in Identifying the Central Sensitivity Syndrome in Patients With Chronic Pain With and Without Structural Pathology, The Clinical journal of pain 31(11) (2015) 959-67.

12. Central sensitization inventory (CSI)

The CSI is a self-report questionnaire that has been validated in several studies and that can be used both as a screener and as treatment outcome measure.

It has been translated in 12 different languages, also in Dutch, and is free online available on www.pridedallas.com. It has also been validated in many different languages (Dutch, Spanish, Italian, Japanese, Serbian etc.).

Many research has been performed to validate the CSI, and many excellent test characteristics have been found. In the systematic review of Scerbo [14] these characteristics are listed very clearly.

One study mentioned the developing of a short form of the CIS with 9 items [15]. The CSI-9 items include: 1. Unrefreshed in morning; 2. Muscles stiff/achy; 3. Pain all over body; 4. Headaches; 5. Do not sleep well; 6. Difficulty concentrating; 7. Stress makes symptoms worse; 8. Tension in neck and shoulders; 9. Poor memory.

Test characteristics:

Cross-cultural validity (the degree to which the performance of the items on a translated or culturally adapted instrument are an adequate reflection of the performance of the items of the original version of the instrument): the CSI is translated and validated in several languages and performs well.

Internal consistency (the degree to which subsections of an instrument measure the same concept or construct; the degree of interrelatedness among the items): Cronbach’s a for all items of the CSI was around 0,9, demonstrating a good degree of internal consistency [15-18].

Reliability (the extent to which repeated measurements agree with one another. This can include measurement over time (test-retest), by different persons on the same occasion (interrater), or by the same person(s) on different occasions (intrarater)): patients with diagnoses of at least 1 central sensitivity syndrome (CSS) (n = 89) scored, on average, 21.5 points higher on the CSI than the nonpatient control group (n = 129) and 11.5 points higher than the non-CSS patient sample (n = 32); P < 0.001. Sensitivity = 0.81, specificity = 0.75 [19].

Responsiveness (the ability of a measure to detect change over time in the phenomenon of interest): the responsiveness of the CSI has also not been investigated so far. This may be due to the fact that there is no CS gold standard to which compare the CSI scores [20].

Structural validity (the degree to which the scorers of a health related patient-reported outcome (HR-PRO) instrument are an adequate reflection of the dimensionality of the construct to be measured): Normative mean score (149 nontreatment) = 28.9, chronic low back pain mean score = 41.6, chronic widespread pain mean score = 47.5, fibromyalgia mean score = 58.2. Higher scores in populations with conditions the CSI is designed to capture indicate good structural validity [21].

Construct validity (the degree to which the scores of an HR-PRO instrument are consistent with hypotheses based on the assumption that the HR-PRO instrument validly measures the construct to be measured): “The CSI is deemed to identify if a patient's symptoms may be related to CS. However, the CSI includes items measuring different constructs, such as physical functioning, psychological functioning, cognitive functioning, headache, and others. While it is probably true that all these constructs may be related to CS, it is still uncertain whether it is appropriate to conclude that the instrument measures CS” [20].

Face validity (the degree to which (the items of) an HR-PRO instrument indeed look as though they are an adequate reflection of the construct to be measured): personal evaluation

Interpretability (the degree to which one can assign qualitative meaning to an instrument’s quantitative scores or change in scores):

1. 374 participants scored ≥ 40 on the CSI and 290 scored < 40.

2. High (> 40) preoperative CSI scores were associated with higher postoperative disability questionnaire scores (P = 0.009 after adjustment for demographic variables, operation duration, and preoperative health status). This indicates that preoperative CSI scores can potentially be used to predict postoperative health.

3. After adjustment, every 10-point increase in score on the CSI was associated with a 6.4% increase in postoperative length of stay in the hospital (P = 0.035)

4. A non statistically significant but noteworthy finding: after adjustment, every 10-point increase in score on the CSI decreased odds of being discharged home by 17% (P = 0.0709) [22].

Criterion validity (the degree to which the scores of an HR-PRO instrument are an adequate reflection of a gold standard): a majority of the patient sample was diagnosed with more than 1 CSS. A higher number of CSS diagnoses were consistently associated with higher CSI scores. The number of CSS diagnoses and CSI scores were significantly correlated (r = 0.51, P < 0.001) [19].

| 1 | I feel tired and unrefreshed when I wake from sleeping. | Never | Rarely | Sometimes | Often | Always |
| --- | --- | --- | --- | --- | --- | --- |
|  |  |  |  |  |  |  |
| 2 | My muscles feel stiff and achy. | Never | Rarely | Sometimes | Often | Always |
|  |  |  |  |  |  |  |
| 3 | I have anxiety attacks. | Never | Rarely | Sometimes | Often | Always |
|  |  |  |  |  |  |  |
| 4 | I grind or clench my teeth. | Never | Rarely | Sometimes | Often | Always |
|  |  |  |  |  |  |  |
| 5 | I have problems with diarrhea and/or constipation. | Never | Rarely | Sometimes | Often | Always |
|  |  |  |  |  |  |  |
| 6 | I need help in performing my daily activities. | Never | Rarely | Sometimes | Often | Always |
|  |  |  |  |  |  |  |
| 7 | I am sensitive to bright lights. | Never | Rarely | Sometimes | Often | Always |
|  |  |  |  |  |  |  |
| 8 | I get tired very easily when I am physically active. | Never | Rarely | Sometimes | Often | Always |
|  |  |  |  |  |  |  |
| 9 | I feel pain all over my body. | Never | Rarely | Sometimes | Often | Always |
|  |  |  |  |  |  |  |
| 10 | I have headaches. | Never | Rarely | Sometimes | Often | Always |
|  |  |  |  |  |  |  |
| 11 | I feel discomfort in my bladder and/or burning when I urinate. | Never | Rarely | Sometimes | Often | Always |
|  |  |  |  |  |  |  |
| 12 | I do not sleep well. | Never | Rarely | Sometimes | Often | Always |
|  |  |  |  |  |  |  |
| 13 | I have difficulty concentrating. | Never | Rarely | Sometimes | Often | Always |
|  |  |  |  |  |  |  |
| 14 | I have skin problems such as dryness, itchiness, or rashes. | Never | Rarely | Sometimes | Often | Always |
|  |  |  |  |  |  |  |
| 15 | Stress makes my physical symptoms get worse. | Never | Rarely | Sometimes | Often | Always |
|  |  |  |  |  |  |  |
| 16 | I feel sad or depressed. | Never | Rarely | Sometimes | Often | Always |
|  |  |  |  |  |  |  |
| 17 | I have low energy. | Never | Rarely | Sometimes | Often | Always |
|  |  |  |  |  |  |  |
| 18 | I have muscle tension in my neck and shoulders. | Never | Rarely | Sometimes | Often | Always |
|  |  |  |  |  |  |  |
| 19 | I have pain in my jaw. | Never | Rarely | Sometimes | Often | Always |
|  |  |  |  |  |  |  |
| 20 | Certain smells, such as perfumes, make me feel dizzy and | Never | Rarely | Sometimes | Often | Always |
|  | nauseated. |  |  |  |  |  |
|  |  |  |  |  |  |  |
| 21 | I have to urinate frequently. | Never | Rarely | Sometimes | Often | Always |
|  |  |  |  |  |  |  |
| 22 | My legs feel uncomfortable and restless when I am trying to go | Never | Rarely | Sometimes | Often | Always |
|  | to sleep at night. |  |  |  |  |  |
|  |  |  |  |  |  |  |
| 23 | I have difficulty remembering things. | Never | Rarely | Sometimes | Often | Always |
|  |  |  |  |  |  |  |
| 24 | I suffered trauma as a child. | Never | Rarely | Sometimes | Often | Always |
|  |  |  |  |  |  |  |
| 25 | I have pain in my pelvic area. | Never | Rarely | Sometimes | Often | Always |
|  |  |  |  |  |  |  |
|  |  |  |  |  |  |  |

NO YES Year Diagnosed

Restless Leg Syndrome

Chronic Fatigue Syndrome

Fibromyalgia

Temporomandibular Joint Disorder (TMJ)

Migraine or tension headaches

Irritable Bowel Syndrome

Multiple Chemical Sensitivities

Neck Injury (including whiplash)

Anxiety or Panic Attacks

Depression

References CSI

From systematic review:

J. Kregel, P.J. Vuijk, F. Descheemaeker, D. Keizer, R. van der Noord, J. Nijs, B. Cagnie, M. Meeus, P. van Wilgen, The Dutch Central Sensitization Inventory (CSI): Factor Analysis, Discriminative Power, and Test-Retest Reliability, The Clinical journal of pain 32(7) (2016) 624-30.

G. Mayer, R. Neblett, H. Cohen, K.J. Howard, Y.H. Choi, M.J. Williams, Y. Perez, R.J. Gatchel, The development and psychometric validation of the central sensitization inventory, Pain practice : the official journal of World Institute of Pain 12(4) (2012) 276-85.

R. Neblett, M.M. Hartzell, H. Cohen, T.G. Mayer, M. Williams, Y. Choi, R.J. Gatchel, Ability of the central sensitization inventory to identify central sensitivity syndromes in an outpatient chronic pain sample, The Clinical journal of pain 31(4) (2015) 323-32.

Neblett, H. Cohen, Y. Choi, M.M. Hartzell, M. Williams, T.G. Mayer, R.J. Gatchel, The Central Sensitization Inventory (CSI): establishing clinically significant values for identifying central sensitivity syndromes in an outpatient chronic pain sample, The journal of pain : official journal of the American Pain Society 14(5) (2013) 438-45.

Scerbo, J. Colasurdo, S. Dunn, J. Unger, J. Nijs, C. Cook, Measurement Properties of the Central Sensitization Inventory: A Systematic Review, Pain practice : the official journal of World Institute of Pain 18(4) (2018) 544-554.

C.P. van Wilgen, P.J. Vuijk, J. Kregel, L. Voogt, M. Meeus, F. Descheemaeker, D. Keizer, J. Nijs, Psychological Distress and Widespread Pain Contribute to the Variance of the Central Sensitization Inventory: A Cross-Sectional Study in Patients with Chronic Pain, Pain practice : the official journal of World Institute of Pain 18(2) (2018) 239-246.

Additional search:

T. Nishigami, K. Tanaka, A. Mibu, M. Manfuku, S. Yono, A. Tanabe, Development and psychometric properties of short form of central sensitization inventory in participants with musculoskeletal pain: A cross-sectional study, PloS one 13(7) (2018) e0200152.

K. Tanaka, T. Nishigami, A. Mibu, M. Manfuku, S. Yono, Y. Shinohara, A. Tanabe, R. Ono, Validation of the Japanese version of the Central Sensitization Inventory in patients with musculoskeletal disorders, PloS one 12(12) (2017) e0188719.

A. Knezevic, R. Neblett, M. Jeremic-Knezevic, S. Tomasevic-Todorovic, K. Boskovic, P. Colovic, A. Cuesta-Vargas, Cross-Cultural Adaptation and Psychometric Validation of the Serbian Version of the Central Sensitization Inventory, Pain practice : the official journal of World Institute of Pain 18(4) (2018) 463-472.

A.I. Cuesta-Vargas, C. Roldan-Jimenez, R. Neblett, R.J. Gatchel, Cross-cultural adaptation and validity of the Spanish central sensitization inventory, SpringerPlus 5(1) (2016) 1837

A. Chiarotto, C. Viti, A. Sulli, M. Cutolo, M. Testa, D. Piscitelli, Cross-cultural adaptation and validity of the Italian version of the Central Sensitization Inventory, Musculoskeletal science & practice 37 (2018) 20-28.

R. Neblett, T.G. Mayer, The Central Sensitization Inventory (CSI): some background and current trends, The spine journal : official journal of the North American Spine Society 17(11) (2017) 1766-1767.

J. Gervais-Hupe, J. Pollice, J. Sadi, L.C. Carlesso, Validity of the central sensitization inventory with measures of sensitization in people with knee osteoarthritis, Clinical rheumatology 37(11) (2018) 3125-3132.

R.A. Coronado, S.Z. George, The Central Sensitization Inventory and Pain Sensitivity Questionnaire: An exploration of construct validity and associations with widespread pain sensitivity among individuals with shoulder pain, Musculoskeletal science & practice 36 (2018) 61-67.

J. Kregel, C. Schumacher, M. Dolphens, A. Malfliet, D. Goubert, D. Lenoir, B. Cagnie, M. Meeus, I. Coppieters, Convergent Validity of the Dutch Central Sensitization Inventory: Associations with Psychophysical Pain Measures, Quality of Life, Disability, and Pain Cognitions in Patients with Chronic Spinal Pain, Pain practice : the official journal of World Institute of Pain 18(6) (2018) 777-787.

W. Caumo, L.C. Antunes, J.L. Elkfury, E.G. Herbstrith, R. Busanello Sipmann, A. Souza, I.L. Torres, V. Souza Dos Santos, R. Neblett, The Central Sensitization Inventory validated and adapted for a Brazilian population: psychometric properties and its relationship with brain-derived neurotrophic factor, Journal of pain research 10 (2017) 2109-2122.

R. Neblett, M.M. Hartzell, M. Williams, K.R. Bevers, T.G. Mayer, R.J. Gatchel, Use of the Central Sensitization Inventory (CSI) as a treatment outcome measure for patients with chronic spinal pain disorder in a functional restoration program, The spine journal: official journal of the North American Spine Society 17(12) (2017) 1819-1829.

Appendix 1: List of participants

**National experts Figure 2: flowchart participants**

**
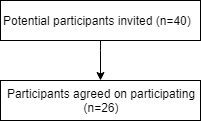

GPs with special MUS clinical and research expertise
Tim olde Hartman
Peter Lucassen**

**Juul Houwen**

**GPs with special MUS clinical expertise:
Nikki Makkes
Annemarie Munster
Jan Willem Ek

GPs with research expertise:
Wim Willems
Wim Verstappen
Otto Maarsingh**

**MUS experts
Yanda van Rood, psychologist**

**Judith van Rosmalen, professor psychosomatics UMCG Groningen**

Irene van Balken, neurologist (specialised in MUS)

Emile Keuter, neurologist (specialised in MUS) **Matthijs Rümke, fysiotherapist**

Dr. Marloes Thoomes-de Graaf, physio-manual therapist

Annet de Jong, fysiotherapist

**Chronic pain experts
Paul van Wilgen
Doeke Keizer, GP
Rob de Bie**

**René Castien**

**Jeroen Kregel**

**André Wolff**

**International experts
Germany:
Maria Kleinstäuber: Maria conducts research in health psychology, abnormal psychology and clinical psychology, at the moment in Auckland.**

**United Kingdom:
Chris Dowrick: professor at department of psychological sciences, university of Liverpool**

**Chris Burton: GP, professor with a particular interest in how doctors and patients deal with persistent physical symptoms, university of Sheffield**

**United States:
David Clarke: clinical assistant professor of gastroenterology emeritus, Oregon Health and Science University, Portland.**

**Belgium
Mira Meeus: fysiotherapist, professor in rehabilitation sciences and kinesiotherapy, university of Antwerp and Gent**

**Jessica van Oosterwijck, v**isiting professor at the University of Antwerp and post-doctoral researcher at Ghent University

Appendix 2: measurement instruments from our systematic review

| Measurement instrument | What is measured? | Examples |
| --- | --- | --- |
| Quantitative sensory testing (QST) | Hyperalgesia, allodynia, temporal summation | Thermal stimuli: thresholds for cold pain, heat pain, cold detection and heat detection; e.g., putting the hand in an iced water bath  Tactile stimuli: pressure pain thresholds (PPTs)  Vibratory or vibrotactile stimuli: detection thresholds for vibration or combination of tactile and vibratory stimuli, e.g., electric toothbrush  Electrical stimuli: reaction to electrical pulses with electrodes  Distention: distending the rectum or oesophagus with an inflatable balloon  Ischemic stimuli: ischemic compression of the arm with a cuff  Reaction on specific pain mediators, e.g. reaction on injection with hypertonic saline |
| Two different quantitative sensory tests together | Conditioned pain modulation (CPM) | Tonic phasic stimulation: phasic heat test with counter irritation of cold [  Ischemic stimulation: inflating an occlusion cuff, comparing pressure pain prior to and during cuff inflation  The nociception withdrawal reflex e.g. H(offman) reflex: stimulation of median nerve with an EMG device, measurement of H wave (a compound muscle action potential)  Measurement of the cutaneous silent period (CSP): a brief pause in muscle action potentials following strong stimulation of a cutaneous nerve during a sustained voluntary contraction |
| MRI*, fMRI*, PET*, somatosensory evoked potentials (SEP) | Structural and functional brain changes | Measurement of changes in brain morphology (global and regional gray matter volumes), changes in density and changes in signaling |
| Measurement of cytokine levels | Laboratory evaluation | Measurement of serum levels of pro-inflammatory interleukines (Il-1, IL-6, IL-8) and anti-inflammatory interleukines (IL-4, IL-10); serum levels of TNF-alpha, a pro-inflammatory cytokine |
| Measurement of neurotrophin levels | Laboratory evaluation | Measurement of serum levels of nerve growth factor (NGF) and brain derived neurotrophic factor (BDNF) |
| Questionnaires | Symptoms, history of functional syndromes | Central sensitization Inventory (CSI) |
|  | Sensory aspects of hypersensitivity | Sensory Hypersensitivity Scale (SHS) |

Appendix 3: search strategy Delphi procedure

We selected 20 studies from our systematic review.

We conducted additional searches in PubMed, the search was conducted per measurement instrument category.

Search terms:

“Central Nervous System Sensitization”[Mesh]

“Central Sensitization” OR “Central Sensitisation” OR “Central Nervous System Sensitization” OR “Central Nervous System Sensitisation”.

AND

(all the following search terms were combined individually with abovementioned search terms)

Thermal stimulation

Tactile stim* OR “Pressure pain threshold*”

Vibratory OR vibrotactile stim*

Electrical stimuli

Ischemic stimulat*

Tonic phasic stimulation

Nociception withdrawal reflex OR H reflex OR Hoffman reflex

Cutaneous silent period

Cytokine*

Neurotrophin* OR Nerve growth factor OR NGF OR Brain derived neurotrophic factor (BDNF)

Central sensitization Inventory OR CSI

Sensory Hypersensitivity Scale OR SHS

Filter: humans

We wanted to select measurement instruments, which are suitable for use in general practice. It is important to know:

1. The properties of the tests: background, methods and costs, investigated population, diagnostic test performance and the results.
2. How the test must be carried out: availability of instruments or materials needed, time needed to perform the test, burden on patient and possibility of an assistant performing the test.

This information and an overview of the corresponding empirical evidence will be provided for all the instruments.

We excluded publications that are not focussing on CS, written in other languages than English, German, French or Dutch and publications that report on animal studies. Two authors (CdB and CG) screened the search results and independently selected publications based on title and abstract. Both authors only included selected publications that are available as full text. These publications were discussed together.
In case the search provided too many publications (>100), the following search term was added:
AND specif*

Figure 3: Flowchart search


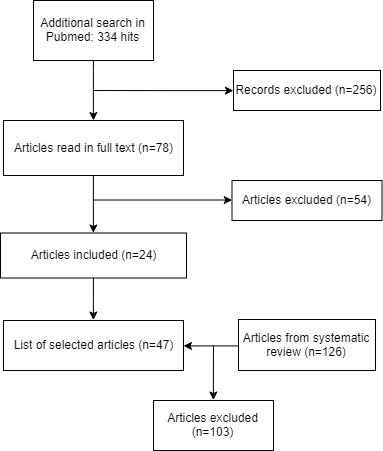


Vibratory or vibrotactile stimuli

From systematic review: 1 [23]

Additional search terms: ((“Central Sensitization” OR “Central Sensitisation” OR “Central Nervous System Sensitization” OR “Central Nervous System Sensitisation” OR “Central Nervous System Sensitization”[Mesh])) AND (Vibratory OR vibrotactile stim*)

Hits: 5

Full text read: 2

Included: 1 [24]

Thermal stimuli

From systematic review: 0

Additional search terms: ((((“Central Nervous System Sensitization”[Mesh] OR “Central Sensitization” OR “Central Sensitisation” OR “Central Nervous System Sensitization” OR “Central Nervous System Sensitisation”)) AND (Thermal stimulation OR Thermal)))

114 hits, added specificity: 6 hits

Full text read: 3

Included: 2 [25, 26]

Tactile stimuli

From systematic review: 1 [27]

Additional search terms: (((((((“Central Nervous System Sensitization”[Mesh] OR “Central Sensitization” OR “Central Sensitisation” OR “Central Nervous System Sensitization” OR “Central Nervous System Sensitisation”))))) AND (tactile stim* OR pressure pain thresholds OR PPT OR pressure pain threshold)))

187 hits, added specificity: 7 hits

Full text read: 5

Included: 2 [28, 29]

Electrical stimuli

From systematic review: 1 [26]

Additional search terms: (((((((“Central Nervous System Sensitization”[Mesh] OR “Central Sensitization” OR “Central Sensitisation” OR “Central Nervous System Sensitization” OR “Central Nervous System Sensitisation”))))))) AND Electrical stimuli

Hits: 38

Full text read: 2

Included: 1 [30]

Ischemic stimuli

From systematic review: 0

Additional search terms: (((((“Central Nervous System Sensitization”[Mesh] OR “Central Sensitization” OR “Central Sensitisation” OR “Central Nervous System Sensitization” OR “Central Nervous System Sensitisation”))))) AND (ischemic OR ischemic stim*)

Hits: 18

Full text read: 3

Included: 0

CPM: Tonic phasic stimulation

From systematic review: 2 [31, 32]

Additional search terms: ((((((“Central Nervous System Sensitization”[Mesh] OR “Central Sensitization” OR “Central Sensitisation” OR “Central Nervous System Sensitization” OR “Central Nervous System Sensitisation”))))) AND (Tonic phasic stimulation OR tonic OR phasic OR counterirritation))

Hits: 26

Full text read: 6

Included: 1 [33]

CPM: ischemic stimulus and pressure point thresholds (PPT)

From systematic review: 2 [34, 35]

Additional search terms: : (((((“Central Nervous System Sensitization”[Mesh] OR “Central Sensitization” OR “Central Sensitisation” OR “Central Nervous System Sensitization” OR “Central Nervous System Sensitisation”))))) AND (ischemic OR ischemic stim*)

Hits: 18

Full text read: 3

Included: 0

The nociception withdrawal reflex (NFR)

From systematic review: 1 [26]

Additional search terms: ((((((“Central Nervous System Sensitization”[Mesh] OR “Central Sensitization” OR “Central Sensitisation” OR “Central Nervous System Sensitization” OR “Central Nervous System Sensitisation”))))) AND (The nociception withdrawal reflex OR NFR OR H-reflex OR Hoffman)

Hits: 18

Full text read: 8

Included: 1[36]

The cutaneous silent period (CSP)

From systematic review: 2 [37, 38]

Additional search terms: (((((“Central Nervous System Sensitization”[Mesh] OR “Central Sensitization” OR “Central Sensitisation” OR “Central Nervous System Sensitization” OR “Central Nervous System Sensitisation”))))) AND (cutaneous silent period OR CSP)

Hits 7

Full text read: 3

Included: 1[39]

Cytokines: TNF-alpha, pro-inflammatory (IL-1, IL-6, IL-8) and anti-inflammatory (IL-4, Il-10)

From systematic review: 2 [40, 41]

Additional search terms: ((((((((“Central Nervous System Sensitization”[Mesh] OR “Central Sensitization” OR “Central Sensitisation” OR “Central Nervous System Sensitization” OR “Central Nervous System Sensitisation”))))) AND ((TNF)))) OR (interleukins AND cytokin*))

Hits: 19

Full text read: 12

Included: 2 [42, 43]

NGF and BDNF

From systematic review: 3 [3, 40, 44]

Search terms: (("Central Nervous System Sensitization"[Mesh] OR "Central Sensitization" OR "Central Sensitisation" OR "Central Nervous System Sensitization" OR central nervous system sensitisation)) AND ((NGF OR nerve growth factor) OR (BDNF OR brain derived neurotrophic factor))

Hits: 88

Full text read: 12

Included: 2 [45]

Central sensitization inventory

From systematic review: 7 [14, 19, 21, 46-49]

Search terms: (((("Central Nervous System Sensitization"[Mesh] OR "Central Sensitization" OR "Central Sensitisation" OR "Central Nervous System Sensitization" OR central nervous system sensitisation)))) AND central sensitization inventory

Hits: 69

Full text reading: 19

Included: 11 [15-18, 20, 50-55]

Sensory hypersensitivity scale (SHS)

From systematic review: 1 [56]

Search terms: (("Central Nervous System Sensitization"[Mesh] OR "Central Sensitization" OR "Central Sensitisation" OR "Central Nervous System Sensitization" OR central nervous system sensitisation)) AND sensory hypersensitivity scale

15 hits

Full text reading: 0

The SHS is not available, only in the article it is mentioned, therefore excluded.

References

[1] C.J. Woolf, Evidence for a central component of post-injury pain hypersensitivity, Nature 306(5944) (1983) 686-8.

[2] B. Walitt, M. Čeko, J.L. Gracely, R.H. Gracely, Neuroimaging of central sensitivity syndromes: Key insights from the scientific literature, Current rheumatology reviews 12(1) (2016) 55-87.

[3] S.A. Zanette, J.A. Dussan-Sarria, A. Souza, A. Deitos, I.L. Torres, W. Caumo, Higher serum S100B and BDNF levels are correlated with a lower pressure-pain threshold in fibromyalgia, Molecular pain 10 (2014) 46.

[4] IASP taxonomy, 2018. <https://www.iasp-pain.org/Taxonomy>.

[5] P. Hansson, Translational aspects of central sensitization induced by primary afferent activity: What it is and what it is not, Pain 155(10) (2014) 1932-1934.

[6] C.J. Woolf, Central sensitization: implications for the diagnosis and treatment of pain, Pain 152(3 Suppl) (2011) S2-15.

[7] S. Kaya, L. Hermans, T. Willems, N. Roussel, M. Meeus, Central sensitization in urogynecological chronic pelvic pain: A systematic literature review, Pain physician 16(4) (2013) 291-308.

[8] N. Stabell, A. Stubhaug, T. Flægstad, E. Mayer, B.D. Naliboff, C.S. Nielsen, Widespread hyperalgesia in adolescents with symptoms of irritable bowel syndrome: Results from a large population-based study, The Journal of Pain 15(9) (2014) 898-906.

[9] J. Nijs, M. Meeus, J. van Oosterwijck, K. Ickmans, G. Moorkens, G. Hans, L.S. de Clerck, In the mind or in the brain? Scientific evidence for central sensitisation in chronic fatigue syndrome, European Journal of Clinical Investigation 42(2) (2012) 203-212.

[10] C. den Boer, B. Terluin, J.C. van der Wouden, A. H. Blankenstein, C. P. van Wilgen, P. Lucassen, H. E. van der Horst, Central sensitization in chronic pain and medically unexplained symptom research: A systematic review of definitions, operationalizations and measurement instruments, Journal of Psychosomatic Research 117(2 (2019)) 32-40.

[11] J. Jones, D. Hunter, Consensus methods for medical and health services research, BMJ (Clinical research ed.) 311(7001) (1995) 376-80.

[12] O.R. Maarsingh, J. Dros, H.C. van Weert, F.G. Schellevis, P.J. Bindels, H.E. van der Horst, Development of a diagnostic protocol for dizziness in elderly patients in general practice: a Delphi procedure, BMC family practice 10 (2009) 12.

[13] R. Meijer, D. Ihnenfeldt, M. Vermeulen, R. De Haan, J. Van Limbeek, The use of a modified Delphi procedure for the determination of 26 prognostic factors in the sub-acute stage of stroke, International journal of rehabilitation research. Internationale Zeitschrift fur Rehabilitationsforschung. Revue internationale de recherches de readaptation 26(4) (2003) 265-70.

[14] T. Scerbo, J. Colasurdo, S. Dunn, J. Unger, J. Nijs, C. Cook, Measurement Properties of the Central Sensitization Inventory: A Systematic Review, Pain practice : the official journal of World Institute of Pain 18(4) (2018) 544-554.

[15] T. Nishigami, K. Tanaka, A. Mibu, M. Manfuku, S. Yono, A. Tanabe, Development and psychometric properties of short form of central sensitization inventory in participants with musculoskeletal pain: A cross-sectional study, PloS one 13(7) (2018) e0200152.

[16] K. Tanaka, T. Nishigami, A. Mibu, M. Manfuku, S. Yono, Y. Shinohara, A. Tanabe, R. Ono, Validation of the Japanese version of the Central Sensitization Inventory in patients with musculoskeletal disorders, PloS one 12(12) (2017) e0188719.

[17] A. Knezevic, R. Neblett, M. Jeremic-Knezevic, S. Tomasevic-Todorovic, K. Boskovic, P. Colovic, A. Cuesta-Vargas, Cross-Cultural Adaptation and Psychometric Validation of the Serbian Version of the Central Sensitization Inventory, Pain practice : the official journal of World Institute of Pain 18(4) (2018) 463-472.

[18] A.I. Cuesta-Vargas, C. Roldan-Jimenez, R. Neblett, R.J. Gatchel, Cross-cultural adaptation and validity of the Spanish central sensitization inventory, SpringerPlus 5(1) (2016) 1837.

[19] R. Neblett, M.M. Hartzell, T.G. Mayer, H. Cohen, R.J. Gatchel, Establishing Clinically Relevant Severity Levels for the Central Sensitization Inventory, Pain practice : the official journal of World Institute of Pain 17(2) (2017) 166-175.

[20] A. Chiarotto, C. Viti, A. Sulli, M. Cutolo, M. Testa, D. Piscitelli, Cross-cultural adaptation and validity of the Italian version of the Central Sensitization Inventory, Musculoskeletal science & practice 37 (2018) 20-28.

[21] T.G. Mayer, R. Neblett, H. Cohen, K.J. Howard, Y.H. Choi, M.J. Williams, Y. Perez, R.J. Gatchel, The development and psychometric validation of the central sensitization inventory, Pain practice : the official journal of World Institute of Pain 12(4) (2012) 276-85.

[22] E.E. Bennett, K.M. Walsh, N.R. Thompson, A.A. Krishnaney, Central Sensitization Inventory as a Predictor of Worse Quality of Life Measures and Increased Length of Stay Following Spinal Fusion, World neurosurgery 104 (2017) 594-600.

[23] D.R. Nixdorf, A. Hemmaty, J.O. Look, E.L. Schiffman, M.T. John, Electric toothbrush application is a reliable and valid test for differentiating temporomandibular disorders pain patients from controls, BMC Musculoskelet Disord 10 (2009) 94.

[24] L.B. Campi, P.C. Jordani, H.L. Tenan, C.M. Camparis, D.A. Goncalves, Painful temporomandibular disorders and central sensitization: implications for management-a pilot study, International journal of oral and maxillofacial surgery 46(1) (2017) 104-110.

[25] R. Staud, E.E. Weyl, D.D. Price, M.E. Robinson, Mechanical and heat hyperalgesia highly predict clinical pain intensity in patients with chronic musculoskeletal pain syndromes, The journal of pain : official journal of the American Pain Society 13(8) (2012) 725-35.

[26] J.A. Desmeules, C. Cedraschi, E. Rapiti, E. Baumgartner, A. Finckh, P. Cohen, P. Dayer, T.L. Vischer, Neurophysiologic evidence for a central sensitization in patients with fibromyalgia, Arthritis and rheumatism 48(5) (2003) 1420-9.

[27] P. de la Coba, S. Bruehl, M. Moreno-Padilla, G.A. Reyes Del Paso, Responses to Slowly Repeated Evoked Pain Stimuli in Fibromyalgia Patients: Evidence of Enhanced Pain Sensitization, Pain medicine (Malden, Mass.) 18(9) (2017) 1778-1786.

[28] A.Y. Neziri, A. Limacher, P. Juni, B.P. Radanov, O.K. Andersen, L. Arendt-Nielsen, M. Curatolo, Ranking of tests for pain hypersensitivity according to their discriminative ability in chronic neck pain, Regional anesthesia and pain medicine 38(4) (2013) 308-20.

[29] A.Y. Neziri, M. Curatolo, A. Limacher, E. Nuesch, B. Radanov, O.K. Andersen, L. Arendt-Nielsen, P. Juni, Ranking of parameters of pain hypersensitivity according to their discriminative ability in chronic low back pain, Pain 153(10) (2012) 2083-91.

[30] J. Van Oosterwijck, J. Nijs, M. Meeus, L. Paul, Evidence for central sensitization in chronic whiplash: a systematic literature review, European journal of pain (London, England) 17(3) (2013) 299-312.

[31] J.B. Correa, L.O. Costa, N.T. de Oliveira, K.A. Sluka, R.E. Liebano, Central sensitization and changes in conditioned pain modulation in people with chronic nonspecific low back pain: a case-control study, Exp Brain Res 233(8) (2015) 2391-9.

[32] J.S. Heymen, Central processing of noxious stimuli in patients with irritable bowel syndrome compared to healthy controls, ProQuest Information & Learning, US, 2007, pp. 1976-1976.

[33] S.T. Skou, T. Graven-Nielsen, L. Lengsoe, O. Simonsen, M.B. Laursen, L. Arendt-Nielsen, Relating clinical measures of pain with experimentally assessed pain mechanisms in patients with knee osteoarthritis, Scandinavian journal of pain 4(2) (2013) 111-117.

[34] L. Arendt-Nielsen, H. Nie, M.B. Laursen, B.S. Laursen, P. Madeleine, O.H. Simonsen, T. Graven-Nielsen, Sensitization in patients with painful knee osteoarthritis, Pain 149(3) (2010) 573-81.

[35] L. Daenen, J. Nijs, N. Roussel, K. Wouters, M. Van Loo, P. Cras, Dysfunctional pain inhibition in patients with chronic whiplash-associated disorders: An experimental study, Clinical rheumatology 32(1) (2013) 23-31.

[36] V. Skljarevski, N.M. Ramadan, The nociceptive flexion reflex in humans -- review article, Pain 96(1-2) (2002) 3-8.

[37] S.H. Baek, H.Y. Seok, Y.S. Koo, B.J. Kim, Lengthened Cutaneous Silent Period in Fibromyalgia Suggesting Central Sensitization as a Pathogenesis, PloS one 11(2) (2016) e0149248.

[38] O. Kilinc, S. Sencan, T. Ercalik, P.K. Koytak, H. Alibas, O.H. Gunduz, T. Tanridag, K. Uluc, Cutaneous silent period in myofascial pain syndrome, Muscle & nerve 57(1) (2018) E24-e28.

[39] O. Sahin, S. Yildiz, N. Yildiz, Cutaneous silent period in fibromyalgia, Neurological research 33(4) (2011) 339-43.

[40] A. Deitos, J.A. Dussan-Sarria, A. Souza, L. Medeiros, G. Tarrago Mda, F. Sehn, M. Chassot, S. Zanette, A. Schwertner, F. Fregni, I.L. Torres, W. Caumo, Clinical Value of Serum Neuroplasticity Mediators in Identifying the Central Sensitivity Syndrome in Patients With Chronic Pain With and Without Structural Pathology, The Clinical journal of pain 31(11) (2015) 959-67.

[41] E. Generaal, N. Vogelzangs, G.J. Macfarlane, R. Geenen, J.H. Smit, J. Dekker, B.W. Penninx, Basal inflammation and innate immune response in chronic multisite musculoskeletal pain, Pain 155(8) (2014) 1605-12.

[42] F. Euteneuer, M.J. Schwarz, A. Hennings, S. Riemer, T. Stapf, V. Selberdinger, W. Rief, Psychobiological aspects of somatization syndromes: contributions of inflammatory cytokines and neopterin, Psychiatry research 195(1-2) (2012) 60-5.

[43] H. Wang, M. Schiltenwolf, M. Buchner, The role of TNF-alpha in patients with chronic low back pain-a prospective comparative longitudinal study, The Clinical journal of pain 24(3) (2008) 273-8.

[44] W. Caumo, A. Deitos, S. Carvalho, J. Leite, F. Carvalho, J.A. Dussán-Sarria, M.G.L. Tarragó, A. Souza, I.L.S. Torres, F. Fregni, Motor cortex excitability and BDNF levels in chronic musculoskeletal pain according to structural pathology, Frontiers in Human Neuroscience 10(2016JULY) (2016).

[45] H. Wang, M. Buchner, M.T. Moser, V. Daniel, M. Schiltenwolf, The role of IL-8 in patients with fibromyalgia: a prospective longitudinal study of 6 months, The Clinical journal of pain 25(1) (2009) 1-4.

[46] J. Kregel, P.J. Vuijk, F. Descheemaeker, D. Keizer, R. van der Noord, J. Nijs, B. Cagnie, M. Meeus, P. van Wilgen, The Dutch Central Sensitization Inventory (CSI): Factor Analysis, Discriminative Power, and Test-Retest Reliability, The Clinical journal of pain 32(7) (2016) 624-30.

[47] R. Neblett, M.M. Hartzell, H. Cohen, T.G. Mayer, M. Williams, Y. Choi, R.J. Gatchel, Ability of the central sensitization inventory to identify central sensitivity syndromes in an outpatient chronic pain sample, The Clinical journal of pain 31(4) (2015) 323-32.

[48] R. Neblett, H. Cohen, Y. Choi, M.M. Hartzell, M. Williams, T.G. Mayer, R.J. Gatchel, The Central Sensitization Inventory (CSI): establishing clinically significant values for identifying central sensitivity syndromes in an outpatient chronic pain sample, The journal of pain : official journal of the American Pain Society 14(5) (2013) 438-45.

[49] C.P. van Wilgen, P.J. Vuijk, J. Kregel, L. Voogt, M. Meeus, F. Descheemaeker, D. Keizer, J. Nijs, Psychological Distress and Widespread Pain Contribute to the Variance of the Central Sensitization Inventory: A Cross-Sectional Study in Patients with Chronic Pain, Pain practice : the official journal of World Institute of Pain 18(2) (2018) 239-246.

[50] R. Neblett, T.G. Mayer, The Central Sensitization Inventory (CSI): some background and current trends, The spine journal : official journal of the North American Spine Society 17(11) (2017) 1766-1767.

[51] J. Gervais-Hupe, J. Pollice, J. Sadi, L.C. Carlesso, Validity of the central sensitization inventory with measures of sensitization in people with knee osteoarthritis, Clinical rheumatology 37(11) (2018) 3125-3132.

[52] R.A. Coronado, S.Z. George, The Central Sensitization Inventory and Pain Sensitivity Questionnaire: An exploration of construct validity and associations with widespread pain sensitivity among individuals with shoulder pain, Musculoskeletal science & practice 36 (2018) 61-67.

[53] J. Kregel, C. Schumacher, M. Dolphens, A. Malfliet, D. Goubert, D. Lenoir, B. Cagnie, M. Meeus, I. Coppieters, Convergent Validity of the Dutch Central Sensitization Inventory: Associations with Psychophysical Pain Measures, Quality of Life, Disability, and Pain Cognitions in Patients with Chronic Spinal Pain, Pain practice : the official journal of World Institute of Pain 18(6) (2018) 777-787.

[54] W. Caumo, L.C. Antunes, J.L. Elkfury, E.G. Herbstrith, R. Busanello Sipmann, A. Souza, I.L. Torres, V. Souza Dos Santos, R. Neblett, The Central Sensitization Inventory validated and adapted for a Brazilian population: psychometric properties and its relationship with brain-derived neurotrophic factor, Journal of pain research 10 (2017) 2109-2122.

[55] R. Neblett, M.M. Hartzell, M. Williams, K.R. Bevers, T.G. Mayer, R.J. Gatchel, Use of the Central Sensitization Inventory (CSI) as a treatment outcome measure for patients with chronic spinal pain disorder in a functional restoration program, The spine journal : official journal of the North American Spine Society 17(12) (2017) 1819-1829.

[56] E.A. Dixon, G. Benham, J.A. Sturgeon, S. Mackey, K.A. Johnson, J. Younger, Development of the Sensory Hypersensitivity Scale (SHS): a self-report tool for assessing sensitivity to sensory stimuli, Journal of behavioral medicine 39(3) (2016) 537-50.
