# Supplementary material for: Tests for central sensitization in general practice: a Delphi study
Source: BMC Fam Pract. 2021 Oct 19;22:206. doi: 10.1186/s12875-021-01539-0 (PMC8527602; doi:10.1186/s12875-021-01539-0)
Supplement: Supplementary file 3 — Additional file 3: Appendix 3. Survey and appendix additional tests first round [file 12875_2021_1539_MOESM3_ESM.docx]

Appendix 3: survey and appendix additional tests first round

Tests for central sensitization in general practice: a Delphi **study**

Carine den Boer, MD^1^

Berend Terluin MD, PhD^1^
Johannes C. van der Wouden PhD^1^
Annette H. Blankenstein MD, PhD^1^
Henriëtte E. van der Horst MD, PhD^1^

1. Amsterdam UMC, location VUmc, Department of General Practice, Amsterdam Public Health research institute, the Netherlands.

Correspondence:

C. den Boer

Amsterdam UMC, location VUmc

Department of General Practice

Amsterdam Public Health research institute

Van der Boechorststraat 7

1081 BT Amsterdam

The Netherlands

Telephone: +31613693267

Email: [c.denboer@amsterdamumc.nl](mailto:c.denboer@amsterdamumc.nl)

Additional measurement instruments Delphi procedure

In the first round of our Delphi procedure we asked whether you had any suggestions for additional measurement instruments for central sensitization that we should consider for our study.

We received a number of suggestions, which we have discussed in our team. We have performed additional searches and ultimately selected another three instruments for the Delphi procedure.

As before, we provide summarized relevant information for each of the instruments, and in the Appendix more detailed information is provided.

We hope you will be able to send your completed form within two weeks. After we have received and processed your answers we will continue with the second round of the Delphi procedure.

Measurement instruments:

Sensory hypersensitivity scale 2

Monofilaments 3

Clothes peg 4

Appendix

Sensory hypersensitivity scale 5

Monofilaments 7

Clothes peg 9

Search strategy 11

Reference list 11

Sensory hypersensitivity scale

Background

The Sensory Hypersensitivity Scale (SHS) is a 25-item self-report measure of sensory hypersensitivity. The SHS assesses both general sensitivity and modalityspecific sensitivity (e.g. touch, taste, and hearing). The aim in developing this tool was to focus on the sensory aspects of hypersensitivity, largely independent from psychological constructs of depression and anxiety. The SHS appears suitable as a screening measure for sensory hypersensitivity, though additional research is warranted to determine its suitability as a proxy for central sensitization.

Method

Patients have to fill out the questionnaire, the researcher has to score the questions and interpret the results. For the moment, the test is only available in the original publication (E.A. Dixon, G. Benham, J.A. Sturgeon, S. Mackey, K.A. Johnson, J. Younger, Development of the Sensory Hypersensitivity Scale (SHS): a self-report tool for assessing sensitivity to sensory stimuli, Journal of Behavioral Medicine 39(3) (2016) 537-50).

Investigated population

1202 participants (157 individuals with chronic pain, the other healthy controls, most students)

Results

Overall SHS scores showed significant but relatively modest correlations (Pearson’s r) with three measures of sensory testing: cold pain tolerance (−0.34); heat pain tolerance (−0.285); heat pain threshold (−0.271). Women reported significantly higher scores on the SHS than men, although gender-based differences were small. In the chronic pain group, individuals with fibromyalgia demonstrated significantly higher SHS scores than did individuals with osteoarthritis or back pain.

| Sensory hypersensitivity scale (SHS) | Score (+,-,?) | Motivation |
| --- | --- | --- |
| Technical feasibility |  |  |
| Added value test |  |  |
| Overall judgment: suitable for use in general practice? YES/NO | | |

Quantitative sensory testing: monofilaments (Semmes Weinstein)

Background

Semmes Weinstein monofilaments have been developed as a set of 20 filamets, but a mini set of 5 filaments has also been used.

Method

Mechanical temporal summation and slowly repeated evoked pain (SREP) is assessed using monofilaments of different weights. To measure mechanical temporal summation the monofilament is applied 30 times with a rate of 1/sec and the patients have to rate the pain.

To measure SREP the series of slowly repeated evoked pain consist of 9 supra-threshold painful pressure stimuli with monofilaments 5 seconds in duration with an interstimulus interval of 30 seconds and patients have to rate the pain.

Investigated population

Fibromyalgia patients, rheumatoid arthritis patients, healthy controls

Results

Compared with temporal summation, slowly repeated evoked pain (SREP) demonstrated higher overall diagnostic accuracy (87.7% versus 64.6%), greater sensitivity (0.89 versus 0.57), and greater specificity (0.87 versus 0.73) in discriminating between fibromyalgia and rheumatoid arthritis patients. SREP demonstrated higher specificity in discriminating fibromyalgia and control groups relative to pain threshold or tolerance.

| Monofilaments | Score (+,-,?) | Motivation |
| --- | --- | --- |
| Technical feasibility |  |  |
| Added value test |  |  |
| Overall judgment: suitable for use in general practice? YES/NO | | |

Clothes peg

Background

A clothes peg can be used to measure pain sensitivity. Clothes pegs can have different clamping forces which can be calibrated.

Method

A calibrated clothes peg is applied for 10 seconds and patients rate the pain intensity on a 0 to 10 numerical rating scale. In one study clothes pegs tests were compared to measurement of pain detection threshold (PPdt) and pressure pain tolerance threshold (PPtt) with a standard (electronic) algometer. Both methods, clothes pegs and test with the algometer, were performed on both middle fingers and ear lobes.

Investigated population

157 inpatients with different pain types (orthopedic, psychosomatic)

Results

Clothes peg values correlate at a clinically meaningful level with pressure pain detection thresholds (PPdt) and pressure pain tolerance thresholds (PPtt) measured by an electronic algometer. Clothes peg values correlated with PPdt values for finger testing with r = -0.54 and for earlobe testing with r = -0.55, values of r > 0,5 correspond to a large correlation.

| Clothes peg | Score (+,-,?) | Motivation |
| --- | --- | --- |
| Technical feasibility |  |  |
| Added value test |  |  |
| Overall judgment: suitable for use in general practice? YES/NO | | |

Appendix

1. Sensory hypersensitivity scale

The Sensory Hypersensitivity Scale (SHS) is a 25-item self-report measure of sensory hypersensitivity. The SHS assesses both general sensitivity and modalityspecific sensitivity (e.g. touch, taste, and hearing). The aim of the development of this tool was to focus on the sensory aspects of hypersensitivity, largely independent from psychological constructs of depression and anxiety.

Five studies were performed to validatie this questionnaire with 1202 participants (157 individuals with chronic pain), The SHS demonstrated an adequate overall internal reliability (Cronbach’s alpha) of 0.81, suggesting the tool can be used as a cross-modality assessment of sensitivity. SHS scores demonstrated only modest correlations (Pearson’s r) with depressive symptoms (0.19) and anxiety (0.28), suggesting a low level of overlap with psychiatric complaints. Overall SHS scores showed significant but relatively modest correlations (Pearson’s r) with three measures of sensory testing: cold pain tolerance (−0.34); heat pain tolerance (−0.285); heat pain threshold (−0.271). Women reported significantly higher scores on the SHS than did men, although gender-based differences were small. In a chronic pain sample, individuals with fibromyalgia syndrome demonstrated significantly higher SHS scores than did individuals with osteoarthritis or back pain. The SHS appears suitable as a screening measure for sensory hypersensitivity, though additional research is warranted to determine its suitability as a proxy for central sensitization.

Materials needed for measurement: test on paper or online (not yet available)
Availability of materials needed for measurement: test not available online, only from the research article

Burden on patient: light, answering 25 questions on a 5-point scale

Time needed to apply the test: 10 minutes to fill in, 5 minutes to score

Ability of an assistant or practice nurse to perform the test: easy

The Sensory Hypersensitive Scale (Likert scale 1-5):

I suffer from allergies

I am allergy-free

I have a number of allergies

I often feel too hot in an environment where others don’t seem to be bothered

I am easily disturbed by high temperatures

I often feel too cold in an environment where others don’t seem to be bothered

I am easily disturbed by low temperatures

My eyes are sensitive to sunlight

I am sensitive to bright light

I am not really bothered by bright lights

I am quite sensitive to pain

I can tolerate a large amount of pain

Things that would ordinarily hurt others are not painful to me

I often react to odors that other do not initially notice

I seem to notice smells that other people do not

I rarely notice smells

When I read, it must be totally quiet

I cannot study or read if there is any conversation or noise around

I can work even in noisy circumstances

I tend to be a picky eater

There are many foods that taste bad to me

I can eat almost anything

I am generally unable to wear clothes made of rough material

I am sensitive to rough textures

I can wear almost any kind of fabric without it bothering me

References:

E.A. Dixon, G. Benham, J.A. Sturgeon, S. Mackey, K.A. Johnson, J. Younger, Development of the Sensory Hypersensitivity Scale (SHS): a self-report tool for assessing sensitivity to sensory stimuli, Journal of Behavioral Medicine 39(3) (2016) 537-50.

QST: monofilaments (Von Frey/ Semmes Weinstein)

Background

Max von Frey used horse hair of various thickness to test sensibility. Later Semmes and Weinstein developed nylon variants. A normal testkit consists of 20 filaments, a mini set of 5 filaments has also been used.

Method

Allodynia, mechanical temporal summation and SREP (slowly repeated evoked pain) is assessed using monofilaments of different weights. To measure allodynia the monofilament is applied a few times and patients were asked to rate the pain. To measure mechanical temporal summation the monofilament is applied 30 times with a rate of 1/sec and the patients have to rate the pain.

To measure SREP the SREP series consists of 9 suprathreshold painful pressure stimuli 5 seconds in duration with an interstimulus interval of 30 seconds and patients have to rate the pain.

Investigated population

Fibromyalgia patients, rheumatoid arthritis patients, healthy controls

For all studies:

Materials needed for measurement: set of monofilaments

Availability of materials needed for measurement: easy to buy, a standardized set of 5 filaments is reliable enough and costs about 100 euro.

Burden on patient: low burden on patients

Time needed to apply the test: a filament is placed 1.5 sec. on the skin, kept for 1.5 sec on the skin and in 1.5 sec. removed. The filaments 1.65-4.08 must been placed 3x after each other, that is in one session to assess the pressure pain threshold. Than all filaments are 3x tested (so the first tested filaments are 9x tested). The monofilament has to be placed perpendicular to the skin and pressure must be carried out until the filament bows in a C. Total time 10-15 minutes.
Ability of an assistant or practice nurse to perform the test: easy to learn

Study 1:

Method: a standard mechanical TSP protocol (10 stimuli of 1-second duration at the thenar eminence using a 300-g monofilament with 1 second interstimulus interval) and the SREP protocol (9 suprathreshold pressure stimuli of 5-second duration applied to the fingernail with a 30-second interstimulus interval). To evaluate reliability for both protocols, they were repeated in a second session 4-7 days later.

To assess subjective pain intensity, a 10-cm visual analogue scale (VAS) was completed after each stimulus, with the anchors being “no pain” and “extremely painful.” The difference in pain assessment formats between the TSP and SREP protocols was necessitated by the different rates at which pain stimuli were presented in each. The standard TSP protocol uses a verbal NRS response format because of the need for rapid assessment after each pain stimulus (in our case, 1 pain rating/second). The SREP protocol is much slower and allows for the use of a written VAS (1 pain rating/30 seconds). For consistency with their originally published SREP protocol, the authors elected to maintain the VAS to assess the SREP pain stimuli, while using the required NRS format for pain assessment in the TSP protocol. To minimize any potential confounding influence of the different pain assessment formats and to insure that responses to both were as similar as possible, each participant received instructions and training in use of both NRS and VAS ratings before the primary study procedures

Investigated population: Thirty-five fibromyalgia (FM) patients and 30 rheumatoid arthritis (RA) patients completed, in pseudorandomized order

Results: Evidence for significant pain sensitization over trials (increasing pain intensity ratings) was observed for SREP in FM (p < .001) but not in RA (p = .35), whereas significant sensitization was observed in both diagnostic groups for the TSP protocol (p < .008). Compared with TSP, SREP demonstrated higher overall diagnostic accuracy (87.7% versus 64.6%), greater sensitivity (0.89 versus 0.57), and greater specificity (0.87 versus 0.73) in discriminating between FM and RA patients. Test-retest reliability of SREP sensitization was good in FM (intraclass correlations = 0.80), and moderate in RA (intraclass correlations = 0.68).

Study 2

Method: A SREP protocol was administered to all subjects, consisting of a single series of nine low-intensity pressure stimuli of five-second duration and thirty-second interstimulus interval. Subjective evoked pain intensity was assessed with a visual analogical scale. Clinical fibromyalgia pain was assessed with the McGill Pain Questionnaire.

Investigated population: twenty-four fibromyalgia patients and 24 healthy participants

Results: perceived pain intensity increased during the SREP protocol in fibromyalgia patients but not in healthy participants. Neither pain threshold nor pain tolerance was associated with SREP. Degree of SREP sensitization was associated with McGill Pain Questionnaire-Sensory ratings of fibromyalgia pain. The effect size for differences between the fibromyalgia and healthy control groups was greater, and the overlaps of the groups distributions lower, for SREP sensitization than for traditional evoked pain measures of pain threshold and tolerance. SREP demonstrated higher specificity in discriminating fibromyalgia and control groups relative to pain threshold or tolerance.

References:

Study 1: P. de la Coba, S. Bruehl, M. Moreno-Padilla, G.A. Reyes Del Paso, Responses to Slowly Repeated Evoked Pain Stimuli in Fibromyalgia Patients: Evidence of Enhanced Pain Sensitization. Pain Medicine (Malden, Mass.) 18(9) (2017) 1778-1786.

Study 2: P. de la Coba, S. Bruehl, C.M. Galvez-Sanchez, G.A. Reyes Del Paso, Slowly Repeated Evoked Pain as a Marker of Central Sensitization in Fibromyalgia: Diagnostic Accuracy and Reliability in Comparison With Temporal Summation of Pain. Psychosom Med 80(6) (2018) 573-580.

Clothes peg

Background

A clothes peg can be used to measure pain sensitivity. Clothes pegs can have different clamping forces which can be calibrated.

Method

A calibrated clothes peg was applied for 10 seconds and patients rated the pain intensity on a 0 to 10 numerical rating scale. Pressure pain detection threshold (PPdt) and pressure pain tolerance threshold (PPtt) were measured with a standard electronic algometer. Both methods were performed on both middle fingers and ear lobes.

To measure pressure pain detection threshold on the middle finger pressure is gradually increased until the pressure is considered painful, this was performed 3 times. To measure pressure pain tolerance threshold was measured applying the algometer to the middle finger of the dominant hand until maximally tolerable pain level, this was performed one time.

The pressure of the clothes peg on the middle finger is considered to be beneath or slightly above the pressure pain threshold. The pressure pain of the clothes peg on the earlobe is perceived as consistently and clearly above the pain threshold and assesses the ability of the patient to endure pain. Therefore, clothes peg exposure tests integrate aspects of pain sensitivity which are otherwise tested separately.

Investigated population

We tested 157 in-patients with different pain types (orthopedic, psychosomatic). In a subgroup of 47 patients repeatability (test-retest reliability) was calculated.

Materials needed for measurement: calibrated clothes peg, algometer

Availability of materials needed for measurement: clothes peg cheap, equipment to calibrate the clothes peg needed, handheld algometer (270 euro). In this study clothes pegs were used with a clamping force of 10 Newton at an extension of 5 mm.

Burden on patient: low to medium burden on patients

Time needed to apply the test: 15 minutes

Ability of an assistant or practice nurse to perform the test: easy to learn

Results

Clothes peg values correlate at a clinically meaningful level with pressure pain detection tresholds (PPdt) and pressure pain tolerance thresholds (PPtt) measured by an electronic algometer.

Clothes peg values correlated with PPdt values for finger testing with r = -0.54 and for earlobe testing with r = -0.55 (all p-values < 0.001). Clothes peg values also correlated with PPtt values for finger testing with r = -0.55 (p < 0.001). R (rho) is the spearmans rank correlation coefficient. According to Cohen’s conventions, r=0.3-0.5 corresponds to a correlation size of a medium effect and r>0.5 corresponds to a large effect.

Test-retest reliability (repeatability) showed equally stable results for clothes peg algometry and the electronic algometer (all r-values > 0.89, all p-values < 0.001). Conclusions: Information on pain sensitivity provided by a calibrated clothes peg and an established algometer correlate at a clinically meaningful level.

**Pain characteristics of the two clinical groups**. Our aim was to compare the two algometric test methods in a wide range of pain types. Therefore we recruited patients from the orthopaedic department and the medical-psychosomatic department. **The left figure** illustrates the distribution of the baseline pain values (NRS) in both groups. **The right figure**illustrates the distribution of the pain sensitivity values (NRS) of the ear lobe provoked by clothes pegs. The box-and-whisker-plots show the median with interquartile range (box: 25^th^and 75^th^percentile) and 5^th^and 95th percentile (whiskers) of the data distribution.

References:

N. Egloff, N. Klingler, R. von Kanel, R.J. Camara, M. Curatolo, B. Wegmann, E. Marti, M.L. Ferrari, Algometry with a clothes peg compared to an electronic pressure algometer: a randomized cross-sectional study in pain patients. BMC Musculoskelet Disord 12 (2011) 174

Search strategy

Sensory hypersensitivity scale

From systematic review: 1 [1]

Search terms: (("Central Nervous System Sensitization"[Mesh] OR "Central Sensitization" OR "Central Sensitisation" OR "Central Nervous System Sensitization" OR central nervous system sensitisation)) AND sensory hypersensitivity scale

15 hits

Full text reading: 0

Selected: 0

Monofilaments

From systematic review: 0

Search terms: (("Central Nervous System Sensitization"[Mesh] OR "Central Sensitization" OR "Central Sensitisation" OR "Central Nervous System Sensitization" OR central nervous system sensitisation)) AND monofilaments

6 hits

Full text reading: 4

Selected: 0

Additional search terms:

From systematic review: 0

((“Central Sensitization” OR “Central Sensitisation” OR “Central Nervous System Sensitization” OR “Central Nervous System Sensitisation” OR “Central Nervous System Sensitization”[Mesh])) AND temporal summation AND specif*

Hits: 17

Full text read: 5

Included: 2 ([2, 3]

Clothes peg

From systematic review: 0

Search terms: (("Central Nervous System Sensitization"[Mesh] OR "Central Sensitization" OR "Central Sensitisation" OR "Central Nervous System Sensitization" OR central nervous system sensitisation))

AND clothes peg

Full text read: 1

Selected: 1 [4]

References:

[1] E.A. Dixon, G. Benham, J.A. Sturgeon, S. Mackey, K.A. Johnson, J. Younger, Development of the Sensory Hypersensitivity Scale (SHS): a self-report tool for assessing sensitivity to sensory stimuli, Journal of behavioral medicine 39(3) (2016) 537-50.

[2] P. de la Coba, S. Bruehl, M. Moreno-Padilla, G.A. Reyes Del Paso, Responses to Slowly Repeated Evoked Pain Stimuli in Fibromyalgia Patients: Evidence of Enhanced Pain Sensitization, Pain medicine (Malden, Mass.) 18(9) (2017) 1778-1786.

[3] P. de la Coba, S. Bruehl, C.M. Galvez-Sanchez, G.A. Reyes Del Paso, Slowly Repeated Evoked Pain as a Marker of Central Sensitization in Fibromyalgia: Diagnostic Accuracy and Reliability in Comparison With Temporal Summation of Pain, Psychosom Med 80(6) (2018) 573-580.

[4] N. Egloff, N. Klingler, R. von Kanel, R.J. Camara, M. Curatolo, B. Wegmann, E. Marti, M.L. Ferrari, Algometry with a clothes peg compared to an electronic pressure algometer: a randomized cross-sectional study in pain patients, BMC Musculoskelet Disord 12 (2011) 174.
