# Supplementary material for: Tests for central sensitization in general practice: a Delphi study
Source: BMC Fam Pract. 2021 Oct 19;22:206. doi: 10.1186/s12875-021-01539-0 (PMC8527602; doi:10.1186/s12875-021-01539-0)
Supplement: Supplementary file 4 — Additional file 4: Appendix 4. Survey second round [file 12875_2021_1539_MOESM4_ESM.docx]

Appendix 4: survey second round

Tests for central sensitization in general practice: a Delphi **study**

Carine den Boer, MD^1^

Berend Terluin MD, PhD^1^
Johannes C. van der Wouden PhD^1^
Annette H. Blankenstein MD, PhD^1^
Henriëtte E. van der Horst MD, PhD^1^

1. Amsterdam UMC, location VUmc, Department of General Practice, Amsterdam Public Health research institute, the Netherlands.

Correspondence:

C. den Boer

Amsterdam UMC, location VUmc

Department of General Practice

Amsterdam Public Health research institute

Van der Boechorststraat 7

1081 BT Amsterdam

The Netherlands

Telephone: +31613693267

Email: [c.denboer@amsterdamumc.nl](mailto:c.denboer@amsterdamumc.nl)

Amsterdam, 3 April 2019

Dear participant,

Many thanks again for accepting our invitation to participate in our Delphi procedure. Aim of the procedure is to reach consensus on measurement instruments for central sensitization suitable for use in general practice. This is the second round of the Delphi procedure.

In the first round you first rated 12 measurement instruments and we added three instruments, so totally 15 instruments have now been rated.

One of them, the CSI , got more than 70% consensus for yes.

Seven instruments got more than 70% consensus for no, here are the arguments:

The electrical pain and reflex thresholds: unattractive, expensive, EMG not available.

CPM: combination of PPT and cold pain: ice water messy, difficult to control temperature.

CPM: combination of heat and cold stimuli: thermatosensory unit very expensive, ice water messy.

The nociceptive flexion reflex (NFR): valid test but EMG is too expensive for general practice, maybe this can be made available as a diagnostic test in a diagnostic center.

The cutaneous silent period (CSP): valid test but EMG is too expensive for general practice, maybe this can be made available as a diagnostic test in a diagnostic center.

Cytokine level in blood samples: could be useful, but test not yet available in labs.

Neurotrophin level in blood samples: not specific enough for measurement of CS.

This leaves us with seven instruments that did not reach 70% consensus. In this document, we have summarized the information about the seven instruments and their test characteristics. We got some new information from the first round, we added this in red.
With each instrument we provide you with all the comments of the other panelists. You can see which number you have in the invitation email. Finally we have listed the general remarks.

In the appendix we have provided additional background information on the seven tests

For each of the seven measurement instruments, we provide a summary of relevant information. We kindly ask you again to rate each measurement instrument: please try to conclude yes or no. You can motivate your choice.

We will again summarize the results and assess whether each instrument reaches at least 70% agreement after this second round. When necessary we will have another email round as third round.

When you have any questions, don’t hesitate to contact us.

We hope you can find the time to respond within two weeks, before 17 April.

Warm greetings,

Carine den Boer, MD GP, email: [c.denboer@vumc.nl](mailto:c.denboer@vumc.nl);, mobile phone 0031613693267

Amsterdam UMC, Vrije Universiteit Amsterdam
Department of general practice and elderly care medicine

Amsterdam Public Health research institute, the Netherlands

Introduction Delphi procedure round 2

We are asking you to rate seven tests.

Tests 1-5 are quantitative sensory tests (QST). QST is used to measure hyperalgesia (increased sensitivity to painful stimuli), allodynia (painful perception of non-painful stimuli) and temporal summation (TS). TS refers to the phenomenon of increased pain perception in response to repetitive noxious stimuli over time (ascending facilitation, referring to central sensitization). Hyperalgesia and allodynia can also refer to peripheral sensitization, but when measured on another part of the body it refers to CS.

Test 6 is a test of conditioned pain modulation (CPM). CPM refers to the phenomenon (figure 1) that ‘pain inhibits pain’, the reduction in pain sensitivity for a tested stimulus due to the interference of a second stimulus (conditioning stimulus) applied at the same time but to a remote body region. CS leads to a smaller reduction in pain sensitivity due to hyperexcitability of the central nervous system (CNS) and reduction of descending inhibition.

Test 7 is a questionnaire.

1. Electric toothbrush test

Background:

Producing vibrotactile stimuli by an electric toothbrush can cause a (punctuate and dynamic) mechanical and thermal stimulation with temporal summation.

Method:

An electric toothbrush is used to apply vibrotactile stimuli to various areas of the head and arms, 1 pound pressure for 30 seconds in four different areas. Pain intensity is recorded at 0, 15, 30 and 60 seconds on a 0 to 10 scale (<1 minute in total). The test takes 8 minutes (less than 2 minutes per stimulus area).

Investigated population:

Women with temporomandibular disorders (TMD), chronic orofacial pain and arthralgia.

Results:

Sensitivity of 57% and specificity of 92% over all four areas, in one of the tests. Patients had higher pain sensitivity and lower pressure point threshold values compared to the control group.

Comments from the first round (TF= technical feasibility, AVT= added value test):

|  | The electric toothbrush test | Rating |
| --- | --- | --- |
| 1 | TF? Easy to perform, however 8 minutes is quite long duration  AVT ? Moderate sensitivity, has to be performed in combination with other test(s); value in other disorders has yet to be established | no |
| 2 | TF + : easy applicable AVT ? : research population are patients with symptoms which occur not often in general practice | yes |
| 3 | TF+: Easy to use, harmless AVT ?: low sensitivity ; not validated for chronic pain/MUS | no |
| 4 | TF - : It’s just weird and feels home-made / unprofessional. How do you calibrate 1 pound pressure? 8 minutes is quite a lot AVT - : Test just formalises something that could be asked | no |
| 5 | TF + 8 minutes is a long time. Maybe using it on one area ie 2 minutes would be feasible AVT + | ? |
| 6 | TF +  AVT - : Sensitivity is poor | no |
| 7 | TF - : I think it is difficult to exert exactly one pound of pressure with a toothbrush on the different areas. Too much pressure will result in exaggerated stimulation of skin and deep tissue tactile receptors. In order to counteract this potential flaw you would need some sort of pressure algometer that measures the exact force that is applied. The time needed to perform this form of QST is too long for a g.p. setting in my opinion, especially if you want to perform other forms of QST as well. AVT ? : You will probably stimulate both vibratory and tactile (superficial and deep) receptors and perhaps even Aδ-fibres with the toothbrush. It will be impossible to distinguish the afferents that are involved. This form of QST will be sufficient if you are only interested in the question whether this (crude form of) stimulation leads to an increased pain perception (i.e. the presence of c.s.) in comparison to healthy controls. You will not be able to distinguish between the afferent fibres involved. | no |
| 8 | TF + : seems easy applicable, I wonder how often you will use it AVT ? : I can't estimate whether this is a big target population | yes |
| 9 | TF + : Easy to do. Cheap. Less than 10 minutes AVT ? : What does the test add when the diagnosis is known. Small sample and specific diagnosis, no diversity (can the test discriminate in a random sample? extrapolation possible?) Low sensitivity What is the standard? Construct validity: how? | no |
| 10 | TF ? : I lack sufficient knowledge and practical skills for this test AVT ? |  |
| 11 | TF + : Easy to perform, low cost. AVT - : The only evidence for this test that exists is in people with TMD, and I am doubtful this test will have equal results in other populations with central sensitization. I believe it is better to recommend a standard battery of test measures for central sensitization which can be used in different disorders. So no because of limited added value for all CS populations | no |
| 12 | TF + : This seems an easy test, although I am not sure how the pound pressure is guaranteed. The pressure points have been removed from the fibromyalgia diagnosis since they were not very reliable. I wonder how reliable these data will be, and what the kappa between different raters would be. AVT ? : I cannot judge this without more details. What was the gold standard for the sensitivity and specificity? How many patients were in the studies? | no |
| 13 | TF +/- : Test can be expensive (material is needed), easily accessible in general practice AVT + : High specificity | yes |
| 14 | TF + : Does not need expensive technical equipment, 8 minutes is economic AVT + : Economic assessment of biological marker of TS | yes |
| 15 | TF - : Not very hygienic, new brushes for every patient. AVT ? : Sens and spec compared to? What is the gold standard for this? Only applicable for TMJ. | no |
| 16 | TF + : Easy viable in practice AVT ? : Possibly additional in combination with symptoms already present | yes |
| 17 | TF - : The amount of pressure is hard to standardise AVT ? : What as the gold standard to determine sens and spec? | no |
| 18 | TF - : too much time needed for doing the test in GP-setting AVT - : TMD/COP > too small group in total GP population | no |
| 19 | TF + : This test is not too specific nor does it require specialised knowledge. The required time concerns me a bit. Compared to the other suggested tests (often 10-15min), the application time of 8 minutes is acceptable. However, I would prefer tests with a shorter application time (e.g. 3-5min), as we probably aim at a combination of different tests and the duration of a GP consultation is 20min at the max AVT + : In general, it is impossible to assess the added diagnostic value of an index test that lacks a reliable reference standard and a well-defined target condition. Hypothetically, every standardized mechanical/thermal provocation could be considered a possible test. However, in absence of such empirical evidence, consensus opinion may provide some guidance. From that perspective – and based on the limited, low-quality evidence, I can imagine this test has some added value. | yes |
| 20 | TF + : Easy to use AVT + : Can be useful in practise, but no , maybe easier to use a pinprick for temporal summation | no |
| 21 | TF + : seems AVT ? : the added value is modest. The test gives on one hand little false-positive results, but on the other hand much false negative results. Distinction with other causes of pain? | yes |
| 22 | TF + : Easy to administer, not time consuming AVT ? : The validation is not sufficient. The sensitivity/specificity is shown without 95% CI. Generalization of the test is limited. | yes |
| 23 | TF + : Very easy to do AVT ? : It has been tested in a limited number of patients in whom initial pain was already low: methodology is questionable (lumping several pain sites together) | no |
| 24 | TF ? : The practice nurse should be very well instructed and I think experienced when doing this as the patients has to concentrate on quite some things (filling out the NRS pain at the right moment) AVT - : Not tested in the big functional syndromes (IBS, FM, CFS, somatoform disorder | no |
| 25 | TF + : Easy implementable, cheap, easy handling for assistant, but when enough for routine? AVT ? : Moderate test characteristics | yes |
| 26 | TF + : Appears to be easy to learn and apply, cheap, low burden on patient AVT ? : High specificity is okay, sensitivity rather low | yes |
| 27 | TF + : No special instruments or training needed AVT ? : Technical, but otherwise not very impressive (for patients) who seek a somatic explanation for their complaints | yes |

| Electric toothbrush test |  |  |
| --- | --- | --- |
| Overall judgment: suitable for use in general practice? YES/NO | | |

1. The painful heat or cold stimuli test

Background:

Repetitive painful heat or cold stimuli may induce temporal summation. Perception thresholds are assessed, pain thresholds (when is the stimulus experienced as painful) and pain tolerance thresholds (when is the pain felt as intolerable). Lower pain thresholds to cold and hot stimulation have been found in FM patients, suggesting hyperexcitability of the nociceptive system.

Method:

Painful heat and cold test: a thermode applies heat and cold stimuli to the hands and shoulders. The temperature is adjusted and participants are instructed to rate their pain on an visual analogue scale (VAS). Perception and pain thresholds are assessed.

For this test a thermoregulatory unit is needed, this consists of a computer which generates thermal stimuli and a Peltier electrode for administering the stimuli to the skin. The costs for a thermatosensory unit like this are around 4500 euro. The patient experiences pain with a mean VAS of 3 (on a scale of 10).

Investigated population:
Patients with fibromyalgia (FM), local (neck/shoulder) musculoskeletal pain (LMP) and controls

Results:
Heat tests: the studies showed that this test can distinguish between FM and local musculoskeletal pain and between these and control patients.

Cold-heat test: significantly lower pain threshold to cold and hot stimulation in FM patients.

Comments from the first round (TF= technical feasibility, AVT= added value test):

|  | The painful heat or cold stimuli test | Rating |
| --- | --- | --- |
| 1 | TF ? Quite long duration AVT + Seems suitable for also more extended disorders like FM | yes |
| 2 | TF + : VAS is subjective AVT + : materials cost money, but acceptable. Interesting research population for general practice. | yes |
| 3 | TF -: purchase of a thermoregulatory unit might be a problem. Moreover, burned skin is a risk in very sensitive people. Performance quite complex. AVT +: cut-off value can be used as a clear tool in demonstrating and explaining CS. | no |
| 4 | TF - : Complicated – it’s a research tool AVT ? : Nicely demonstrates other pain modalities affected – not just pressure pain | no |
| 5 | TF + : Maybe. Cost may be an issue AVT ? : Assumes FM is a distinct clinical entity, which I doubt | ? |
| 6 | TF - : Too expensive AVT - : FM and local M-S pain can be distinguished quite well via the patient’s history, the chronicity of the symptom. | no |
| 7 | TF ? : I am wondering whether you could actually buy a thermoregulatory unit for only 150 euros?? Can you get it up-and-running for this amount of money? I would assume it costs a lot more to obtain a fully functional thermoregulatory unit. Furthermore, I would like to know how much time it costs to assess the thermal thresholds in patients, both perception and pain thresholds. AVT + : Thermal stimulation is capable of stimulating Aδ-fibres specifically. The presence of any measured lowered thresholds in patients would strongly suggest the presence of central sensitisation. The test results may support and/or confirm the GP’s findings during physical examination. | no |
| 8 | TF ? : I think when we purchase this our therapists or nursing specialists can apply this. Costs not high.Takes time, I don't know if this is feasable AVT + : Fine to differentiate fibromyalgia patients better | yes |
| 9 | TF + : Idem 1. AVT ? : What does the test add when the diagnosis is known. Small sample and specific diagnosis no diversity (can the test discriminate in a random sample? extrapolation possible?)  What is the standard? | no |
| 10 | TF ? : I lack sufficient knowledge and practical skills for this test AVT ? |  |
| 11 | TF + : I am quite surprised that you would be able to acquire a thermode system for only 120 euro. Systems which allow high increases/decreases to hot as well as cold like the Medoc are usually quite expensive. Are you sure the minimum price for a fully working set is only 120 euro? If that is indeed the case I would recommend this assessment, if it is more expensive I would not recommend this. AVT + : Thermal perception, pain and tolerance thresholds measured at local and distant locations are a good indicator for widespread hypersensitivity in different populations with chronic pain. | yes |
| 12 | TF + : This is a widely used test validated in several scientific studies . The costs are limited. The information about time needed is missing. AVT + : It can specifically show how sensitive and tolerant a person is to pain. The measure has a high validity, and can be used to explain to the patient the mechanisms underlying chronic pain in that individual. | yes |
| 13 | TF +/- : Low/medium burden on patient, availability materials is needed AVT - : I think it is not valuable to distinguish patients with FM and local musculoskeletal pain | no |
| 14 | TF ? :Staff/room needed for conducting the test, a significant difference between groups is not a sufficient validation of the measure to use it as FMS marker AVT + : Biological marker for fibromyalgia | no |
| 15 | TF ? : Useful for multiple pain regions. I would love to implement this test into primary care, however I could not find the proper equipment able to generate a specific temperature. I called to Conrad to ask if a thermode data logger and peltier thermode could be used for this purpose, they felt it would not become an applicator that could be regulated. If this cannot be used, costs are high (>4500 euro’s) AVT + : Temperal summation is important to measure | yes |
| 16 | TF + : Easy viable in practice AVT ? : Possibly additional in combination with symptoms already present | yes |
| 17 | TF - : Not possible for clinician. Too expensive.  AVT + : But we need norm values? | no |
| 18 | TF ? : It’s not clear yet: easy to use? How much time needed? Costs + AVT ? : Device has to be linked to practical advice what to do with results (most GPs are not familiar with CS). It is able to distinguish > that’s a +. | ? |
| 19 | TF - : This test is too specific for application in general practice. Also, it requires substantial knowledge of the GP. AVT ? : In general, it is impossible to assess the added diagnostic value of an index test that lacks a reliable reference standard and a well-defined target condition. However, in absence of such empirical evidence, consensus opinion may provide some guidance. For this specific test, I doubt the additional diagnostic value. | no |
| 20 | TF - : Difficult for use in practice, validation in practise is difficult AVT - : | no |
| 21 | TF - : too complicated, expertise needed to aplly, not enough patients to get enough skills AVT ? : no data on sensitivity and specificity | no |
| 22 | TF ? : Not easy to measure patients with this device AVT + : | yes |
| 23 | TF + : Easy to perform, costs are reasonable AVT + : | yes |
| 24 | TF - : Too difficult to perform in daily primary care practice as it is a very technical test (searching for pain tresholds and pain tolerance tresholds is quite difficult in a research setting, but almost impossible in a daily practice setting AVT - : Too much effort | no |
| 25 | TF + : Easy. No so cheap. I think a computer is necessary AVT ?: Unknown testcharacteristics | yes |
| 26 | TF + : Appears to be easy to learn and apply, cheap, acceptable burden on patient AVT ? : Test characteristics not immediately clear | yes |
| 27 | TF - : Too expensive AVT + : Differentiation between FM and musculoskeletal local pain | no |

| The painful heat or cold stimuli test |  |
| --- | --- |
| Overall judgment: suitable for use in general practice? YES/NO | |

1. Pressure pain thresholds (PPT) and pressure tolerance thresholds

Background:

Most QST studies use pressure pain thresholds (PPTs) for measuring central sensitization. Pressure pain detection threshold is set at a point where comfortable pressure turns into pain; the pressure pain tolerance threshold is set at a point where the pain is felt intolerable.

Method:
A pressure algometer performs pressure stimulation on different parts of the body. PPTs are measured by increasing pressure. The patient scores the pain on a VAS scale. The patient can press a button when the pain becomes too much. A pressure algometer can be manual (circa 200 euro) or computer-controlled (circa 2000 euro). The manual algometer has proven to be as reliable as the computer controlled algometer (not yet published research René Castien).

Investigated population:

Patients with FM and chronic pain patients.

Results:
Patients with FM had an increased pain perception intensity compared to control patients. Using PPT as measure of pain hypersensitivity had a good ability in distinguishing both chronic neck pain patients and chronic low back patients from healthy control subjects.

Comments from the first round (TF= technical feasibility, AVT= added value test):

|  | Pressure pain thresholds (PPT) and pressure tolerance thresholds | Rating |
| --- | --- | --- |
| 1 | TF + Easy to perform, takes a little less time AVT + Seems very reliable | yes |
| 2 | TF + : Study 1 is in particular feasible, study 2 and 3 are more complicated and big control group. Handheld algometer is affordable AVT + : Study 1 high specificity and high sensitivity | yes |
| 3 | TF-: purchase of a pressure algometer can be a problem. AVT +: suggestion for modification: not merely the pain after pressure, but the reaction after gentle touch | no |
| 4 | TF - : Specialised technique and equipment AVT - : No point in study comparing chronic pain vs healthy controls. Needs to be chronic non-nociceptive pain vs acute / chronic nociceptive pain. | no |
| 5 | TF +: Looks OK, but cost may be an issue AVT ? | ? |
| 6 | TF - : Expensive AVT - : The patient’s history will provide similar information | no |
| 7 | TF + : I know these small devices that can be applied manually. Although (small) variances will be inevitable with respect to the applied force, it can be easily performed in general practice. I do not know the computer-controlled version of the pressure algometer. AVT + : It may support the phenomenon of c.s. in patients with fibromyalgia, chronic low back pain. | yes |
| 8 | TF ? : like before. Many patients which we don't treat in the hospital. For fysiotherapy practice maybe big investment? AVT + : especially for the patient | ? |
| 9 | TF + : Idem 1 AVT ? : Idem 2 Positive points:  - Three studies, three different categories/syndromes.  - Good test characteristics | no |
| 10 | TF + : Easy to use and relatively low-cost. Important to have trained assessors for a standardized assessment AVT + : Possibly a test with added value when used on multiple body locations (indicative of generalized hyperalgesia). More knowledge on reference values is needed. | yes |
| 11 | TF + : Very easy to use in clinical practice and quite cheap. Note: You can use it without the button but using verbal feedback when to stop the pressure. AVT + : Mechanical pain and tolerance thresholds measured at local and distant locations are a good indicator for widespread hypersensitivity in different populations with chronic pain. | yes |
| 12 | TF - : I assume most GPs would choose manual device, and I suspect that this is very unreliable if used in daily practice. Even if the reliability is OK in scientific studies, this is a completely different situation since such studies are based on well-trained test assistants that perform many of such measures in a short time. This is not comparable to a GP practice. The computer device does not have these problems, but I suspect it to be too expensive. AVT ? : The results can be used for explanations to the patients, but I think we should not use assessments that are not reliable or valid, even if the patient finds them appealing. | no |
| 13 | TF +/- : Materials are available in general practice, disadvantage: medium burden on patient AVT + : High specificity | yes |
| 14 | TF + : Easy to apply, feasible interpretation with cut-off scores AVT + : Well validated test for several chronic pain groups | yes |
| 15 | TF + : Useful for multiple pain regions AVT + : Measures a different concept than test 2, really useful. Normative values are known for some regions. Study 2 and 3 used chronic spine patients, I presume they did not all have CS. | yes |
| 16 | TF + : Easy viable in practice AVT ? : Possibly additional in combination with symptoms already present | yes |
| 17 | TF + : AVT - : What is hyperalgesia and what is normal? | no |
| 18 | TF ? : It’s not clear yet: Easy to use? How much time needed? Costs low + AVT +/? : Device has to be linked to practical advice what to do with results (most GPs are not familiar with CS). It is able to distinguish > That’s a + | ? |
| 19 | TF + : This test is not too specific nor does it require specialised knowledge. The required time is acceptable. The price of 200 euro may be an obstacle for some GPs. AVT + : In general, it is impossible to assess the added diagnostic value of an index test that lacks a reliable reference standard and a well-defined target condition. Hypothetically, every standardized mechanical/thermal provocation could be considered a possible test. However, in absence of such empirical evidence, consensus opinion may provide some guidance. From that perspective – and based on the limited, low-quality evidence, I can imagine this test has some added value. | yes |
| 20 | TF + : Easy to use, quantifying of measure can be useful and widespread pain can be measured, no reference values AVT ? : Useful for evaluation of treatment although correlation between PPT and pain in rather low | yes |
| 21 | TF ? : I don't know enough about the operation of the device. 200 euro is no problem, I think 2000 euro would be too much for incidental use AVT - : The distinction between chronic back pain and healthy controls doesn't say much for the utility in daily practice | no |
| 22 | TF + : Simple and easy to handle test. No differences in measuring with mechanical or electric device AVT + | yes |
| 23 | TF + : Manual is probably just as good as computer controlled AVT + : | yes |
| 24 | TF + : When doing it with computer AVT - : Costs are too high, and giving patients intolerable pressure pain might be an ethical dilemma | no |
| 25 | TF ? : Easy implementable daily practice. High costs. AVT + : Reasonable test characteristics | yes |
| 26 | TF + : Appears to be easy to learn and apply, cheap, acceptable burden on patient. 2000 € would be too costly AVT + : Sensitivity and specificity of the 1st study (wireless pressure algometer – I assume this is the cheaper one) adequate. | yes |
| 27 | TF - : Too expensive AVT - : Does not seem to add value above the less expensive “The painful heat or cold stimuli test” | no |

| Pressure pain thresholds (PPT) and pressure tolerance thresholds |  |
| --- | --- |
| Overall judgment: suitable for use in general practice? YES/NO | |

1. Monofilaments (Semmes Weinstein)

Background

Semmes Weinstein monofilaments have been developed as a set of 20 filamets, but a mini set of 5 filaments has also been used.

Method

Mechanical temporal summation and slowly repeated evoked pain (SREP) is assessed using monofilaments of different weights. To measure mechanical temporal summation the monofilament is applied 30 times with a rate of 1/sec and the patients have to rate the pain.

To measure SREP the series of slowly repeated evoked pain consist of 9 supra-threshold painful pressure stimuli with monofilaments 5 seconds in duration with an interstimulus interval of 30 seconds and patients have to rate the pain.

Investigated population

Fibromyalgia patients, rheumatoid arthritis patients, healthy controls

Results

Compared with temporal summation, slowly repeated evoked pain (SREP) demonstrated higher overall diagnostic accuracy (87.7% versus 64.6%), greater sensitivity (0.89 versus 0.57), and greater specificity (0.87 versus 0.73) in discriminating between fibromyalgia and rheumatoid arthritis patients. SREP demonstrated higher specificity in discriminating fibromyalgia and control groups relative to pain threshold or tolerance.

Comments from the first round (TF= technical feasibility, AVT= added value test):

|  | Monofilaments | Rating |
| --- | --- | --- |
| 1 | TF + Easy to perform; what about duration? Full test vs 5 filaments; filaments break easily, needs instruction although AVT ? Used in many studies, but maybe not suitable for general practice as screening tool; should elaborated further before use in general | no |
| 2 | TF + easy method AVT ? Differentiates between FM and RA. In general practice relatively few RA patients. So control group should be healthy controls. | yes |
| 3 | no answer |  |
| 4 | no answer |  |
| 5 | TF ? Complex to administer in general practice  AVT + Discriminates fibromyalgia from other pain conditions | no |
| 6 |  | no |
| 7 | TF ? : Technical feasibility depends on the test algorithm that is used. In my experience the duration of the quantitative sensory testing should be as short as possible, in order to avoid the patient to become distracted, bored, irritated, etc.QST with monofilaments should be able to be performed in general practice, since application of the stimuli is very simple and reproducible, but the duration of the test algorithm should be as short as possible.  AVT + : QST with Von Frey monofilaments has been demonstrated to be able to assess and quantify the presence and severity of both negative and positive sensory symptoms. | yes |
| 8 | FT : easy to use, direct applicable, little time  AVT: can have added value | yes |
| 9 | TF +: easy AVT -: It discriminates between fibromyalgia and RA, but history taking, physical examination and if necessary blood tests discriminate better. Test-re-test reliability varies between fibromyalgia and RA. How should we interpret this? | no |
| 10 | FT ?: I lack sufficient knowledge and practical skills for this test AVT ?: |  |
| 11 | TF +: Cheap materials, easy to use in clinical practice. AVT -: I question the use of these type of filaments for CS testing. Von Frey filaments are mainly used to examine neuropathic pain (i.e. see German Research Network on Neuropathic Pain (DFNS) quantitative sensory testing (QST)). Small fibre neuropathy has been detected in fibromyalgia so this could explain the results mentioned from the study above. | no |
| 12 | TF +: The test is cheap, not too time-consuming and not too burdensome for patients. AVT +: Based on sensitivity and specificity, it might have added value. | yes |
| 13 | TF +/-: Materials needed, high costs, low burden on patients, time 10-15 minutes (too long), easy to learn  AVT -: Results are only based on diagnostic accuracy | no |
| 14 | TF +: Cheap, easy to apply AVT ?: Well validated, however only in FMS groups | yes |
| 15 | TF +: Accessible, wind up ratio concept? AVT +: | yes |
| 16 | TF +: reasonably easy to implement  AVT ?: dubious, risk of medicalization | yes |
| 17 | TF -: Standardisation is an issue: weights, localisation, etc. AVT +: | yes |
| 18 | TF ? I think the time investment is big AVT ? | ? |
| 19 | TF+ This test is not too specific nor does it require specialised knowledge. The required time is acceptable. AVT ? In general, it is impossible to assess the added diagnostic value of an index test that lacks a reliable reference standard and a well-defined target condition. Hypothetically, every standardized mechanical/thermal provocation could be considered a possible test. | yes |
| 20 | TF + Easy to use  AVT + Evaluation and assessment, especially for evaluation of severe (neuropathic) pain | yes |
| 21 | TF+  AVT + rather good at discriminating fibromyalgia and rheumatoid arthritis | yes |
| 22 | TF +: Easy to administer. AVT +: Research is available on diagnostic accuracy and reliability. | yes |
| 23 | TF +: Easy to do AVT +/-: Differs from the original use, but can add extra information | yes |
| 24 | TF ?: Seems quite difficult to do it in a standardized way AVT +/?: | ? |
| 25 | TF ? Not easy to implement in daily practice. Can assistant do the test? AVT + If executed OK. Reasonable predictive value |  |
| 26 | TF ?: I don't see the how to perform exactly: 5 types of filaments, 30 times. Seems good AVT +: seems distinctive | yes |
| 27 | TF +: Not too expensive, one time investment. Easy to learn and can be carried out by assistant.  AVT +: It is very valid measure. The process of sensitisation is elicited. Discussing the test and its results with the patient can be used to explain the CS process. | yes |

| Monofilaments |  |
| --- | --- |
| Overall judgment: suitable for use in general practice? YES/NO | |

1. Clothes peg

Background

A clothes peg can be used to measure pain sensitivity. Clothes pegs can have different clamping forces which can be calibrated.

Method

A calibrated clothes peg is applied for 10 seconds and patients rate the pain intensity on a 0 to 10 numerical rating scale. In one study clothes pegs tests were compared to measurement of pain detection threshold (PPdt) and pressure pain tolerance threshold (PPtt) with a standard (electronic) algometer. The clothes peg test is performed on both middle fingers and ear lobes.

Investigated population

157 inpatients with different pain types (orthopedic, psychosomatic)

Results

Clothes peg values correlate at a clinically meaningful level with pressure pain detection thresholds (PPdt) and pressure pain tolerance thresholds (PPtt) measured by an electronic algometer. Clothes peg values correlated with PPdt values for finger testing with r = -0.54 and for earlobe testing with r = -0.55, values of r > 0,5 correspond to a large correlation.

Comments from the first round (TF= technical feasibility, AVT= added value test):

|  | Clothes peg | Rating |
| --- | --- | --- |
| 1 | TF + simple  AVT - Quite low correlation with PPT, and not enough data available about scientific properties | no |
| 2 | TF + Relative easy  AVT - Two research methods are compared, I don't know how relevant this is | ? |
| 3 |  |  |
| 4 |  |  |
| 5 | TF + Easy to administer  AVT + Compares well with formal instruments | yes |
| 6 |  | yes |
| 7 | FT + The test can be easily performed. However the clamping force – although calibrated in advance – will inevitably differ, depending on how and where the clamp is attached to the earlobe. Perhaps the clamping end of the clamp must be ‘rounded off’ in order to prevent subtle pinching differences between different clamping positions AVT - This method of QST needs further validation in my opinion. | no |
| 8 | FT : easy to use, direct applicable, little time  AVT: can have added value | yes |
| 9 | TF +: easy AVT -: Correlation with other test is interesting > measuring the same mechanism? | no |
| 10 | FT ?: I lack sufficient knowledge and practical skills for this test AVT ?: |  |
| 11 | TF +: Cheap, easy to use. On the other hand, you need to use different clamps to achieve the wright pressure, so using an algometer seems easier and more straightforward to assess the effect of mechanical pressure. AVT -: I believe the use of an algometer allows more specific assessment of the exact pressure applied, and can be used at more test sites in comparison to the clamp, diminishing the added value of the clamp. | no |
| 12 | TF +: Easy, cheap and fast to apply. I do wonder what patients think it the GP presents a clothes peg. AVT +: Reasonable correlations with gold standard. | yes |
| 13 | TF +/-: Materials needed for measurement High costs Low to medium burden on patients (advantage) Time: 15 minutes (too long) Easy to learn AVT +: Medium effect size between patient groups (orthopaedic vs psychosomatic | yes |
| 14 | TF +: Cheap, easy to apply with less effort AVT: Well validated groups of individuals suffering from different forms of chronic pain Moderate to high associations with other indicators of central sensitisation | yes |
| 15 | TF ?:  AVT ?: Unclear how the PPdt of the ear relates to pain in other areas. | no |
| 16 | TF +: reasonably easy to implement AVT ?: dubious, risk of medicalization | yes |
| 17 | TF -: Applied pressure??? Depends on peg? Localisation? AVT -: Prefer PPT if you want to measure mechanical hyperalgesia | no |
| 18 | TF + simple and quick AVT ? Seems interesting, but not good valuable with these results | yes |
| 19 | TF - Required time (i.e. 15 min) too long, costs (amongst others handheld algometer of 270 euro) too high. AVT ? In general, it is impossible to assess the added diagnostic value of an index test that lacks a reliable reference standard and a well-defined target condition. Hypothetically, every standardized mechanical/thermal provocation could be considered a possible test. | no |
| 20 | TF - Questioning the sensitivity, is the ear useful to measure widespread pain/Central sensitisation, maybe only useful in generalized pain syndromes?   AVT - Seems not useful but I don’t know the research on this calibration? | no |
| 21 | TF+ Seems to me that it is easily applicable AVT ? I’m not aware of any additional scientific evaluation, I’m not sure whether the test has sufficiently been tested concerning discriminatory value | yes |
| 22 | TF +: Will be the cheapest instrument of the sensitisation core-set! Seems promising. AVT ?: Evidence is limited. | no |
| 23 | TF +/-: Calibration of a clothes peg seems troublesome, and I guess after having used the peg for a number of times, the spring will loosen up, necessitating further calibration AVT +/-: Pain threshold and pain duration, if the first does not work, the second cannot be don | no |
| 24 | TF +: AVT ?: | yes |
| 25 | TF + No problem to implement AVT + Reasonable predictive value |  |
| 26 | TF ?: it seems to do, but I don't see us seriously hanging a clothes peg in someone’s ear. I prefer a 'real' device AVT +: seems distinctive | no |
| 27 | TF -: A lot of equipment is needed, and the measure needs to be calibrated. Not clear how often nor how much time it takes to calibrate. Investment 270 euro is a drawback too AVT -: Pain endurance and sensitivity are not separated (if I understand right). It is quit painful (the ear lob clothes peg). | no |

| Clothes peg |  |
| --- | --- |
| Overall judgment: suitable for use in general practice? YES/NO | |

6. CPM: combination of ischemic stimuli and PPT

Background:

Conditioned pain modulation (CPM) means that a test-stimulus and a conditioning stimulus are used together in the test. Combinations of different QST (heat, cold, ischemic, electrical and vibrotactile stimuli e.g.) are used as CPM. We will describe a few combinations. In healthy controls the conditioning stimulus leads to a reduction in the perceived intensity of the test-stimulus. In central sensitization there is a smaller reduction of the perceived intensity of the test-stimulus due to reduced inhibition of descending control.

Method:

The QSTs which are used for CPM combine the following: pressure pain thresholds (PPTs) by an algometer; ischemic stimulation by occlusion cuff (also used for blood pressure measurement) inflated on the arm to a painful intensity (VAS 3 of 4)

Investigated population:
Patients with knee osteoarthritis (KOA), chronic patients with whiplash associated disorder (WAD) and healthy controls.

Results:

TS of pressure pain was significantly decreased in healthy controls compared to patients with chronic WAD. In contrast, TS was quite similar prior to and during cuff inflation. This provides evidence for dysfunctional CPM in these patients. Lack of endogenous pain inhibitory pathways provide additional evidence for presence of CS.

Another study showed a significant increase in PPT during cuff stimulation in controls. These patients with KOA had significant facilitation of TS and significantly less CPM as compared with controls.

Comments from the first round (TF= technical feasibility, AVT= added value test):

|  | CPM: combination of ischemic stimuli and PPT | Rating |
| --- | --- | --- |
| 1 | TF + Not difficult to perform. I see no information about how time consuming this test is AVT ? Results seem quite promising, but no data about sensitivity/specificity? Has to be elaborated on further | no |
| 2 | TF ? : Error prone and subjective AVT ? : Study 2 relevant for general practice, study 1 and 3 more for specialists. Study 1 no control group | no |
| 3 | TF - : performance is complex. Uncomfortable for the patient. Physiotherapists usually don’t use an occlusion cuff.  AVT ? : somewhat premature; not validated for chronic pain/MUS | no |
| 4 | TF - : Complicated method and interpretation AVT ? : Better in that demonstrating a problem with a mechanism rather than subjective experience. | no |
| 5 | TF +  AVT ? Looks more of a research tool than a clinical tool | ? |
| 6 | TF + : AVT - : A test has value when it changes our approach to the patient. For patients with MUS or a functional syndrome, the presence or absence of CS won’t change what we should do for the patient. | no |
| 7 | TF - : I reckon this test is way more complex to execute than the tests described above. Is anything known about the increased risk of thromboembolic complications of occluding the arm this long? AVT - : The test results may be too complex for interpretation in general practice | no |
| 8 | TF ? : easy to learn AVT ? : Because this happens also in other pain syndromes, it doesn't make distinction | no |
| 9 | TF - : More complicated than some other tests and probably too much time (no information) AVT - : What does the test add when the diagnosis is known. What is the standard? Positive points: - Two different categories/syndromes - Interesting method/test to try to demonstrate DNIC/CPM | no |
| 10 | TF ? : I lack sufficient knowledge and practical skills for this test AVT ? |  |
| 11 | TF + : Easy to perform and cheap to acquire. AVT - : Tourniquet ischemia activates C fibers, Aβ and Aδ fibers, and these fibers do not only transmit pain. It is possible that specific activation of pain fibers is necessary to induce a pure CPM response. Therefore this protocol should be questioned. So no because of negative added value | no |
| 12 | TF … : The same problems apply as in the PPT. Time needed is missing; I assume costs are comparable to PPT. AVT … : See PPT | no |
| 13 | TF + : Accessible in general practice AVT + : | yes |
| 14 | TF ? : I assume substantial financial and staff effort to run the test AVT ? : Test has been studied only in very specific populations | no |
| 15 | TF ? : Differences in protocol?  AVT + : Important concept | yes |
| 16 | TF + : reasonably easy to implement, but more complex than the first three AVT ? : Possibly additional in combination with symptoms already present | yes |
| 17 | TF + : AVT - : When is response normal/abnormal? | no |
| 18 | TF ? : It’s not clear enough yet: is it easy to use? How much time needed? Costs low + AVT + : Besides diagnostic value for prof it’s insightful for the patient | yes |
| 19 | TF + : This test seems – according to the described studies in the Appendix, I missed the paragraph ‘For all studies’ – easy to use and does require specialised knowledge. The required time is not explicitly described, but seems acceptable. AVT + : In general, it is impossible to assess the added diagnostic value of an index test that lacks a reliable reference standard and a well-defined target condition. Hypothetically, every standardized mechanical/thermal provocation could be considered a possible test. However, in absence of such empirical evidence, consensus opinion may provide some guidance. From that perspective – and based on the limited, low-quality evidence, I can imagine this test has some added value. | yes |
| 20 | TF - : Set up seems a research set up to me AVT - | no |
| 21 | TF - : device is too complicated AVT - : no data about sensitivity or specificity; only compared to healthy controls, not compared to patients which have acute pain or pain of 3-4 weeks | no |
| 22 | TF + AVT + : Important to determine central pain processing/inhibition in addition to measurements on pain sensitivity | yes |
| 23 | TF + : Easy to do AVT + : Clear cut results | yes |
| 24 | TF ? : I am not sure what an algometer is and how that works. Using a pressure band is suitable AVT - : I think it is still unknown what cut-offs you should use in order to establish whether there is CS or not. | no |
| 25 | TF ? : Too expensive. Not quite understandable how to act. Seems difficult AVT ? : Unknown test characteristics | no |
| 26 | TF ? : Appears to be elaborate. Unclear burden on patient. AVT ? : Test characteristics not immediately clear | no |
| 27 | TF + : No large investments needed. Easy to apply. (point of attention is the interference of blood pressure stress with the results) AVT + : Biological instrument, had more face validity for the patient (I presume) | yes |

| CPM: combination of ischemic stimuli and PPT |  |
| --- | --- |
| Overall judgment: suitable for use in general practice? YES/NO | |

7. Sensory hypersensitivity scale

Background

The Sensory Hypersensitivity Scale (SHS) is a 25-item self-report measure of sensory hypersensitivity. The SHS assesses both general sensitivity and modalityspecific sensitivity (e.g. touch, taste, and hearing). The aim in developing this tool was to focus on the sensory aspects of hypersensitivity, largely independent from psychological constructs of depression and anxiety. The SHS appears suitable as a screening measure for sensory hypersensitivity, though additional research is warranted to determine its suitability as a proxy for central sensitization.

Method

Patients have to fill out the questionnaire, the researcher has to score the questions and interpret the results. For the moment, the test is only available in the original publication (E.A. Dixon, G. Benham, J.A. Sturgeon, S. Mackey, K.A. Johnson, J. Younger, Development of the Sensory Hypersensitivity Scale (SHS): a self-report tool for assessing sensitivity to sensory stimuli, Journal of Behavioral Medicine 39(3) (2016) 537-50).

Investigated population

1202 participants (157 individuals with chronic pain, the other healthy controls, most students)

Results

Overall SHS scores showed significant but relatively modest correlations (Pearson’s r) with three measures of sensory testing: cold pain tolerance (−0.34); heat pain tolerance (−0.285); heat pain threshold (−0.271). Women reported significantly higher scores on the SHS than men, although gender-based differences were small. In the chronic pain group, individuals with fibromyalgia demonstrated significantly higher SHS scores than did individuals with osteoarthritis or back pain.

Comments from the first round (TF= technical feasibility, AVT= added value test):

|  | Sensory hypersensitivity scale | Rating |
| --- | --- | --- |
| 1 | TF + Is a questionnaire, can be filled in at home or on line?  AVT ? Has to be established, validated and cross cultural translated | no |
| 2 | TF + AVT + seems to differentiate between arthralgias and chronic pain syndrome/FM | yes |
| 3 |  |  |
| 4 |  |  |
| 5 | FT + Self-complete instrument AVT + Appears to distinguish fibromyalgia from other pain conditions | yes |
| 6 |  | no |
| 7 | TF + : This test can be easily performed. However, a quiet room must be available allowing the patient to fill out the questionnaire.  AVT -: This questionnaire has not yet been validated as far as I know. Why not use the – validated – Central Sensitization Inventory for this purpose? This consists also of 25 questions. | no |
| 8 | FT : easy to use, direct applicable, little time  AVT: can have added value | yes |
| 9 | TF +: Easy to perform AVT -: Already a part of the history taking. Can be useful in an experiment or for follow up. No added value. See also comments on the other tests. Only one study. The test shows little correlation with other tests. How should we interpret this: measuring another concept/modality/entity/mechanism? | no |
| 10 | FT ?: I lack sufficient knowledge and practical skills for this test AVT ?: |  |
| 11 | TF +: Questionnaire easy to use in clinical practice  AVT -: What is the added value of this questionnaire in contrast to asking the patient during anamnesis how they react to different stimuli and examining this during the clinical assessment | yes |
| 12 | TF +: Easy to complete, on paper or online  AVT +: This test is much better focussed on the aspect of hypersensitivity than the CSI. The validity is much higher. | yes |
| 13 | TF +: Light burden on patient Easy ability AVT -: Gender based differences Only tested with patients with fibromyalgia, are there other patient groups as well? | no |
| 14 | TF ?: Short self-report scale; however scale only in original publication available (copyright issues when scale is used in clinical practice?) AVT -: validated only in one study; low correlation with pain tolerance measure: Is that scale a valid measure of central sensitisation? | no |
| 15 | TF +: AVT ?: Correlations are <moderate and cannot replace the physical tests. | yes |
| 16 | TF +: reasonably easy to implement AVT +: additional in combination with symptoms already present | yes |
| 17 | TF +: But are there norm values, cut-offs? AVT +: Like the idea of testing of hypersensitivity independent from psychological factors (in contrast to CSI) But I don’ t know the test. | yes |
| 18 | TF- Takes much time, patient must do this at home or in practice AVT - Small correlations, clinical assessment of physician can do a lot, not much added value | no |
| 19 | TF + Easy-to-use self-report questionnaire that requires little time, effort and expertise of the GP. AVT ? Given the provided information in the Appendix, it is difficult to estimate the added test value. | yes |
| 20 | TF ? Seems interesting although I don’t use this, I am not familiar with it AVT ? The question is if Sensory Hypersensitivity is the same as what we see in patients in pain, interesting to look at correlations, although validity remains a difficult question | no |
| 21 | TF+ Seems to me that it is easily applicable AVT ? I’m not aware of any additional scientific evaluation, I’m not sure whether the test has sufficiently been tested concerning discriminatory value | no |
| 22 | TF +/-: Long list of questions regarding physical signs of sensitisation. Difficult to understand for patients how to score the questions. AVT -: Limited evidence. | no |
| 23 | TF +: Easy to administer AVT -: It does not add much to already existing instruments, and correlation with seems modest, and also not directly related to pain, but more in general to hypersensitivity | no |
| 24 | TF +: Easy to accomplish in daily primary care setting AVT -: Low correlations | no |
| 25 | TF + Easy to fill in Translation seems no problem AVT + Can’t judge added value thoroughly. No information about sensitivity and specificity | yes |
| 26 | TF +: questionnaire with 25 items seems doable   AVT ?: distinctive value seems restricted | yes |
| 27 | TF +: Simple to use, but it first needs to be translated (not difficult) and made available online (not difficult). In due time AVT +: Differentiates between fibromyalgia and other pain disorders which suggests it is a measure for CS. However, more validating studies are needed. | yes |

| Sensory Hypersensitivity Scale |  |
| --- | --- |
| Overall judgment: suitable for use in general practice? YES/NO | |

|  | General remarks |
| --- | --- |
| 1 |  |
| 2 |  |
| 3 | Compliments for your extensive review on an very relevant subject. I filled in the survey as a physiotherapist, specialized in psychosomatics. That is the reason why many tests are not feasible, f.e. lab tests and tests which need an EMG device. Nevertheless, physiotherapists and other professionals who do possess an EMG device (or want to purchase it) might play an important role in diagnosing CS. The value of a test is for me also determined by the amount on which the result of the test is therapeutically useful. |
| 4 | These are research tools, not for routine practice at present. If there was a clear benefit of demonstrating DNIC failure through CPM then it may be appropriate to consider a whole pathway approach, but not a single test. |
| 5 |  |
| 6 | My belief is that Central Sensitization is not the primary cause of MUS, chronic functional syndromes (like FM or IBS) or Bodily Distress Syndrome. I find that CS is the result of psychosocial issues that can be diagnosed and successfully treated. Among these issues are current life stresses, insufficient self-care skills, the prolonged impact of childhood adversity and somatic presentations of depression, PTSD and anxiety. Merely diagnosing CS doesn’t address the underlying cause and leaves the patient no better off. |
| 7 | If you want any more advice of If you need my help in one of your studies, please contact me! |
| 8 |  |
| 9 | Central sensitisation is a useful concept to explain chronic pain and “Medical unexplained pain’. History and physical examination are the basis of the diagnosis and the possible etiology. Starting a treatment from this perspective leads to better results than the concept of end organ damage. In the scientific literature there are a lot of instruments that more or less correlate with the concept/construct of central sensitisation. I wonder if these tests give us more information in clinical practice than the history and physical examination, considering the diagnosis is the gold standard.  If some questions could be answered, I could eventually change mine point of view: - Has research been done to compare the different tests? Is there a correlation between the different tests, including neuro-imaging? Show the different tests the same results in a patient? - Can we predict treatment outcome given the test result? - Is there a greater change of a peripheral abnormality if the test result is negative/lower than a cut-off point? - Can we use it to convince and motivate the patient at the start and by showing progression? Considering these questions and the remarks I made, I would choose tests 1,2,3,10 en 12 to further investigate suitability for clinical practice. |
| 10 | Thank you for the invitation. I added my response for assessments that I have used for research. As I am not a practitioner, my input is mainly based on knowledge from scientific literature and use of tests in scientific experiments |
| 11 | Currently not all the necessary information is always provided to objectively consider the feasibility for the use in general clinical practice. I also wonder of the authors have experience with all these measure to correctly estimate the degree of difficulty for clinicians to use these application and the burden for the patients. |
| 12 | I miss a basic question here: the test characteristics (reliability and validity). An assessment can be technically feasible and have added value for the patient but still measure something else than CS. I think a Delphi study is not the right approach to answer this question, since it needs expert knowledge on all of these tests. The practical aspects of using tests in daily practice can indeed be answered by this study, but in my opinion validity and reliability are the first questions. And these questions can only be answered by experts in the field, based on much more information than provided here. To provide good answers to these questions, I would need to read a lot of literature, so the value of my answers can be questioned (and it seems likely to me that is true for other participants as well). I realize that some of this information is in the review, but this is not a review on the reliability and validity of the tests. It is an exploration of what scientists have used, and only for the questionnaires a short remark on reliability and validity is included. Information on reliability and validity could be in the appendix of the review; I did not have access to that so I did not use that information when answering these questions.  I also noted that the information provided for the different tests differs. Elements that are sometimes but not always included are costs, time needed, the number of patients in the validation study, the burden of patients, the presence of control values, the sensitivity and specificity of the test against a gold standard etc. The presence and absence of these elements and the way the information is presented is might influence the responses of the participants |
| 13 |  |
| 14 | It will be important to implement a battery of tests instead of a single test (e.g., combination of self-report and 1-2 biological markers) |
| 15 | I think it is really important for all the temperature tests to be absolutely sure the cheap solution is indeed capable to do what it should do (warm or cool an application to a specific temperature). Perhaps a guideline with specific products can be provided, otherwise these tests can only be used when using expensive equipment. |
| 16 | In my opinion, CS's characteristic is the individual experience of the patient. Can CS be seen as a disease? I wonder that. In my opinion, it is rather an enlarged individual property that can not be summarized in an instrument. I therefore think that the added value of the instruments mentioned is very limited and that there is especially a risk for medicalisation of such a property when applied. |
| 17 | The big problem with all QST measurements is that we can only interpret them on group level, but not in an individual patient. |
| 18 |  |
| 19 |  |
| 20 |  |
| 21 | It is important with this tests to make the difference between CS and other pain and not between CS and healthy persons. These tests don't make this difference. So I think we are not ready yet for measuring CS. |
| 22 |  |
| 23 | Thanks for the excellent lay-out and added materials (makes it easy and fun to score) |
| 24 |  |
| 25 | Not easy to fill in. Is there enough evidence of all neurophysiological hypothesis? If so, is there enough evidence to already use them in (general) practice. In general poor characteristics. And If so, why implementing diagnostic procedures with e.g. EMG: why not referring to secondary care with much more experience. |
| 26 | I would appreciate a list of abbreviations |
| 27 | Costs are important in determining if the instrument is suitable for regular use by GP. Costs of EMG device https://www.fysiotherapieworks.nl/emg-apparatuur |
