# Supplementary material for: Tests for central sensitization in general practice: a Delphi study
Source: BMC Fam Pract. 2021 Oct 19;22:206. doi: 10.1186/s12875-021-01539-0 (PMC8527602; doi:10.1186/s12875-021-01539-0)
Supplement: Supplementary file 5 — Additional file 5: Appendix 5. Appendix second round [file 12875_2021_1539_MOESM5_ESM.docx]

Appendix 5: appendix second round

Tests for central sensitization in general practice: a Delphi **study**

Carine den Boer, MD^1^

Berend Terluin MD, PhD^1^
Johannes C. van der Wouden PhD^1^
Annette H. Blankenstein MD, PhD^1^
Henriëtte E. van der Horst MD, PhD^1^

1. Amsterdam UMC, location VUmc, Department of General Practice, Amsterdam Public Health research institute, the Netherlands.

Correspondence:

C. den Boer

Amsterdam UMC, location VUmc

Department of General Practice

Amsterdam Public Health research institute

Van der Boechorststraat 7

1081 BT Amsterdam

The Netherlands

Telephone: +31613693267

Email: [c.denboer@amsterdamumc.nl](mailto:c.denboer@amsterdamumc.nl)

Appendix Delphi procedure on measurement instruments for central sensitization round two

Table of contents

Introduction

**The Delphi procedure
Categories on the score form explained**

**Quantitative sensory testing (QST)**

**Conditioned pain modulation (CPM)**

Quantitative sensory tests (QST)

1. Vibratory/vibrotactile stimulus: the electric toothbrush test
2. Thermal stimulus: the painful heat or cold stimuli test
3. Tactile stimulus: pressure pain thresholds (PPT) and pressure tolerance thresholds

4. Monofilaments

5. Clothes peg

Conditioned pain modulation (CPM)

6. CPM: combination of ischemic stimuli and PPT

Questionnaire
7. The sensory hypersensitivity scale

**Appendix 1: Table of measurement instruments from systematic review**

**Search strategy References**

Introduction

Central sensitization (CS) is a mechanism explaining the cause and persistence of symptoms in absence of a specific somatic or psychiatric disease. Various definitions of CS have been proposed. In 1983 Clifford Woolf stated that chronic pain has both a peripheral sensitization component and a central sensitization component with changes in spinal cord activity [1]. This statement resulted in various theories of mechanisms for CS. Different developments such as quantitative sensory testing (QST), neurotransmitter measurement in blood samples and (functional) magnetic resonance imaging ((f)MRI) have been used to acknowledge and measure CS [2, 3].

The International Association for the Study of Pain (IASP) defined CS in 2011: “[CS is] an increased responsiveness of nociceptive neurons in the central nervous system to their normal or sub-threshold afferent input” [4]. Since then authors are using this definition in their articles but also discuss it. Hansson for example expressed his doubts about this definition because it might be too broad [5].

In 2011 Clifford Woolf wrote an article on the implications of CS for the diagnosis and treatment of pain; he concluded that it remains unclear what triggers and sustains CS, and what are the risk factors in individuals for CS [6]. He considered further research necessary to clarify these issues.

CS has been studied in relation to medically unexplained symptoms (MUS) e.g. fibromyalgia (FM), chronic fatigue syndrome (CFS), irritable bowel syndrome (IBS) and chronic pain (pain longer than 3 months) [7-9].

It is important to mention that there is no gold standard for measurement of CS. Many studies imply that specific symptoms and syndromes are linked to central sensitization, or conclude that certain quantitative sensory tests imply CS ; however, some QST can also measure peripheral sensitization.

In conclusion, CS might be the neurophysiological mechanism explaining the abovementioned symptoms. Tests measuring CS have been collected in our systematic review [10]. Until now these tests are not being used in general practice and there are no clear guidelines for the application of these measurement instruments.

To reach consensus on which measurement instruments for CS from our systematic review could be useful in general practice, we are conducting a Delphi procedure to obtain expert opinion. This procedure will consist of two e-mail rounds and possibly a teleconference at the end among doctors and specialists with experience in the field of chronic pain and/or medically unexplained symptoms.

The Delphi procedure

Delphi procedures are conducted when there is contradictory evidence or no scientific evidence on an issue [11-13]. This consensus method is frequently used in developing guidelines and consists of experts rating to what extent they agree with an issue. Disagreement can be resolved in a structured way, in rounds with controlled and repeated feedback, leading to consensus. The threshold for consensus can differ, we have chosen for 70%. Each participant is asked to vote and motivate his or her rating anonymously, this makes the Delphi procedure reliable as the influence of others is ‘neutralized’. The results after the first round are assessed and summarized and used in the next round as additional feedback. Figure 1 provides an overview of our Delphi-procedure.

Figure 1: Delphi procedure


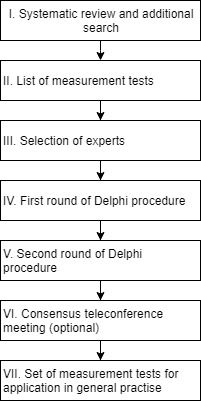


METHODS

Sample of participants

We have compiled a list of potential participants consisting of GPs and other (medical) specialists who have expertise in the domain of MUS and/or chronic pain treatment and research, from the Netherlands as well as from abroad. They received an e-mail with an invitation to participate in this Delphi procedure. Information was provided on the aim of this Delphi-procedure, a summary of the conclusions of our review on definitions, operationalizations and measurement instruments for CS, and an estimation of the time needed to rate the instruments. We offered a modest credit voucher for the effort. A list of participants that agreed on participating is attached in appendix 1.

Selection of measurement instruments

We used the list of the measurement instruments we retrieved from the literature for our systematic review (appendix 2). These comprise various measurement instruments, mostly physical tests, but also questionnaires. We excluded the tests that are inaccessible or too costly for general practice (e.g., brain MRI, fMRI, PET, somatosensory evoked potentials, sensory hypersensitivity scale).

The measurement instruments from our review are divided into categories: quantitative sensory testing (QST), conditioned pain modulation (CPM), measurement of cytokine levels, measurement of neurotrophin levels and questionnaires. We have collected as much relevant information on abovementioned properties for each category, in order to properly rate the measurement instruments. In addition to the articles from the systematic review, we conducted a search in the PubMed database with search terms covering the name of the measurement instrument in relation to central sensitization (appendix 3).

First round

Selected panellists in the Delphi procedure received a list of instruments with the following information:

For each measurement instrument in the survey:

1. Background
2. Method
3. Investigated population
4. Results
5. Score table: the panellists will be asked to rate (with a +/-/?) on two different aspects:

1. technical feasibility; 2. added value of the test, and to motivate their rating. Overall judgement on suitability for use in general practice.

In the appendix this information is extended with (when information is available):

1. Abovementioned information for each study separately

2. Materials needed for measurement

3. Availability of materials needed for measurement

4. Burden for patient;

5. Time needed to apply the test

6. Ability of an assistant or practice nurse to perform the test

7. Reference list.

In case of insufficient expertise with regard to a particular test, the panellist is allowed to refrain from answering. However, we ask the participants to motivate why they can’t provide an answer. Panellists have the opportunity to motivate their rating.

Participants will be asked to complete and return the score form within two weeks by e-mail. When participants want to add tests they think might be relevant, they can add them. These will be added to the first round after assessment by the project team on relevance with regard to measurement of central sensitization.

Assessment of the results

The researchers will summarize the forms returned by the participants. Consensus is reached when 70% or more of the participants (who have returned the form) agree on a test as suitable or not suitable for general practice. If more than 70% conclude that the test is not suitable, we will discard the test. If no agreement has been reached on one or more tests (less agreement than the 70% threshold) or new tests have been added, these will be added to the list for the second round of our Delphi procedure. Each of these tests will have a summary of motivation on different aspects from the panellists.

Second round

Only instruments not reaching the threshold of 70% agreement and added instruments will be included in the second round to the participants. All these instruments will be presented with information on the percentage of agreement per item in the first round, a summary of participant comments and an overview of the participant’s own scores compared to group scores. In this round participants can change their rating of an instrument or motivate their decision again, both in view of the group’s scores. Every participant will be asked to complete a final score and return it via e-mail within two weeks.

Assessment of the results

The researchers will take one week to summarize the results. Similar to after round 1, also in this round instruments will only be included if they reach at least 70% agreement. Tests on which less than 70% agreement has been reached, will be put on a new list with motivations per test. This list will be used when necessary for round 3, which is an organized teleconference with the group of experts to discuss the disagreements. As in round 1 and 2, the threshold for final inclusion of the test is at least 70% consensus of the participants on the test for being suitable in general practice.

Results of the Delphi procedure

The measurement instruments of round 1, 2 and 3 that have been rated by at least 70% of the participants as suitable, will be used in the next phase. Using these results we will develop a diagnostic protocol of measurement instruments to be tested in general practice by general practitioners or their staff.

Explanation of categories score table

Technical feasibility:

How would you rate the feasibility of the test in general practise? Examples of tests not being feasible in general practice are tests requiring too much time, too specific and specialised knowledge or rarely available apparatus. It is obvious that you can have doubts about the technical feasibility.

Added diagnostic value:

What is the added diagnostic value of a test? Does the result of the test enhance the probability of the disease being present of absent, combined with other available information? If a patient already has all the symptoms of a specific MUS syndrome, does this test give any added diagnostic value?

Quantitative sensory testing (QST)

QST describes testing of skin, mucosa or muscle tissue to assess pain or sensory perception pathways. As the name describes, there is a quantification of the patient’s response to types of stimuli (standardized or different types). This can be influenced by the sensitivity of patient’s sensory system or their frame of reference for pain.

The stimulus is usually steadily increasing, and the patient is instructed to notify whenever the sensation in question is for example becoming from comfortable or just pressure to pain. Tolerance can also be used as focus, measuring whenever a stimulus is becoming unbearable for the patient. A(n) (electrical) visual analogue scale (e-VAS) is usually used for assessing the level of sensation or pain.

There are different types of QST: thermal- (cold or heat), vibration- (with different frequencies), pressure- (mechanical) and electrical stimuli. Many studies combine these types of QST. These types will be explained in our measurement instrument list below.

<https://www.youtube.com/watch?v=66B7WiLp-m4>

Conditioned pain modulation (CPM)

Research over the last few decades provided evidence indicating that pain may be dependent of various endogenous pain-inhibitory processes. These are responsible for the experience of pain and operate at different levels of the central nervous system (CNS). Research suggests that (pathological) variations in endogenous pain inhibition could be responsible for the persistence or development of pain.

Investigating the differences of pain inhibition in individuals could be done with the use of CPM. This phenomenon, used to be termed as “Diffuse noxious inhibitory control (DNIC)”, refers to a psychophysical process. A noxious stimulus (the conditioned stimulus) reduces or inhibits the perception of another ‘secondary’ noxious stimulus (the test stimulus) applied to a specific area of the body (dependent of the study). This test surveys the net inhibitory and excitatory effect of nociceptive pathways on sensitivity of pain in a specific body part other than the part of the body being conditioned (with a conditioning stimulus). Reduction of pain perception of the test-stimulus or increase of pain threshold induced by the test-stimulus after receiving a conditioning stimulus is considered as an inhibitory CPM effect. In central sensitization a reduction of inhibition is found due to reduced inhibition of descending control.

1. Vibratory/vibrotactile stimulus: The electric toothbrush test

Background

Producing vibrotactile stimuli by an electric toothbrush can cause a (punctuate and dynamic) mechanical and thermal stimulation with temporal summation (a central nerve system condition with an increased perception to repetitive stimuli related to central sensitization, pain in this case [2]).

Study 1
Method: an electric toothbrush will perform a vibrotactile stimulus with 1 pound pressure for 30 seconds in four different areas. These areas are on the masseter, temporomandibular joint, temporalis and mid-ventral spot of the forearm. At 0, 15, 30 and 60 seconds a pain intensity will be recorded from a 0 to 10 scale.

Investigated population: 14 females with temporomandibular disorders (TMD) with myofascial pain and arthralgia.

Results: Sensitivity of 57% over all 4 areas, specificity of 92% over all 4 areas.

Study 2
Method: see study 1

Investigated population: 16 painful TMD (women), 29 normal control without TMD/chronic orofascial pain (women)

Results: Patients with TMD had higher pain sensitivity and lower pressure point threshold values in trigeminal region (p<0.01) and same results for areas outside the trigeminal region (p<0.01).

For all studies:

Materials needed for measurement: Electric toothbrush, circa 40 euro

Availability of materials needed for measurement: accessible for general practise

Burden on patient: low burden on patient

Time needed to apply the test: 8 minutes (less than 2 minutes per stimulus area)

Ability of an assistant or practice nurse to perform the test: Yes

References
Study 1: D.R. Nixdorf, A. Hemmaty, J.O. Look, E.L. Schiffman, M.T. John, Electric toothbrush application is a reliable and valid test for differentiating temporomandibular disorders pain patients from controls, BMC Musculoskelet Disord 10 (2009) 94.

Study 2: L.B. Campi, P.C. Jordani, H.L. Tenan, C.M. Camparis, D.A. Goncalves, Painful temporomandibular disorders and central sensitization: implications for management-a pilot study, International journal of oral and maxillofacial surgery 46(1) (2017) 104-110.

1. The painful heat or cold stimuli test

Background

Central sensitization is characterised by generalized (widespread) hypersensitivity and enhanced temporal summation of pain. Repetitive painful heat stimuli may induce temporal summation by an enhanced response in dorsal horn neurons by repetitive stimulation of C-fibers. This results in an increased perception of pain with a constant or reduced peripheral input. This is an example of enhanced central excitability, referring to central sensitization.

Study 1
Method: participants received 6 heat stimuli on the hands and shoulders while sitting comfortably. The heat probe was brought on the skin for 10 seconds, from 38 degrees to 44/46. There was an interval of at least 1 minute or until painful aftersensations disappeared between each stimulus. During this test participants used an electronic visual analog scale (e-VAS) for rating their pain intensity.

Investigated population: 36 FM patients (as chronic widespread pain), 24 local musculoskeletal pain patients (LMP), 23 control participants

Results:
Shoulder: Post hoc rating showed significant difference only between control and FM patients (p<0.02), other group comparisons were not different (p>0.05).
Hands: Post hoc testing showed higher significant pain ratings in FM than other groups, also LMP subjects had significantly higher pain ratings compared to control group.

Study 2
Method: participants received the heat and cold stimuli in the inside of the forearm using a thermode (from 0 to 50 degrees). This change in temperature was linear and 1 degree/second, starting from the baseline set on 32 degrees. Perception and pain thresholds were assessed.

Investigated population: 85 FM patients, 40 control participants

Results: Thresholds for cold and heat sensation were similar for FM patients and control participants. FM patients had significantly lower cold and heat pain thresholds (p<0.001 and p=0.005). Cold tolerance was much lower (by 66%) in patients with FM compared to control participants (p<0.001).

For all studies:

Materials needed for measurement: thermal sensory analyser (circa 4500 euro for a basic thermal monitoring analyser), Peltier thermode (circa 10 euro)

Availability of materials needed for measurement: accessible for primary care

Burden on patient: low to medium burden on patient, mean VAS 3, max VAS 4,7 (on a scale of 10)

Time needed to apply the test: <10 minutes

Ability of an assistant or practice nurse to perform the test: yes

References

Study 1: R. Staud, E.E. Weyl, D.D. Price, M.E. Robinson, Mechanical and heat hyperalgesia highly predict clinical pain intensity in patients with chronic musculoskeletal pain syndromes, The journal of pain : official journal of the American Pain Society 13(8) (2012) 725-35.

Study 2: J.A. Desmeules, C. Cedraschi, E. Rapiti, E. Baumgartner, A. Finckh, P. Cohen, P. Dayer, T.L. Vischer, Neurophysiologic evidence for a central sensitization in patients with fibromyalgia, Arthritis and rheumatism 48(5) (2003) 1420-9.

3. Pressure pain thresholds (PPTs) and pressure tolerance thresholds

Background

Most QST studies use pressure pain thresholds (PPT) for measuring central sensitization. In addition there are tests which measure increased perceived pain intensity to slowly repeated evoked pain stimuli (SREP). Next studies evaluate pressure pain thresholds (PPT) and thresholds for tolerance in patients with FM, chronic low back pain and neck pain by using an e-VAS and pain sensitization in FM patients in response to SREP.

Study 1

Method
The intensity of subjective evoked pain is assessed (using a VAS) by applying pressure stimulation on the fingernail with a wireless pressure algometer. The marker of central sensitization is slowly repeated evoked pain (SREP). 9 pain stimuli are given with an unknown timing and duration of the SREP stimuli, with each pain stimulus lasting for 5 seconds with a constant pressure.

Investigated population
24 women with FM and 24 healty participants as control group

Results

Sensitivity of 79% for SREP
Specificity of 92% for SREP

Perception of pain intensity during the SREP stimuli increased in the patients with fibromyalgia and did not increase for the healthy participants.

Study 2

Method: pain pressure threshold and tolerance thresholds were measured using an electronic pressure algometer. Pressure started at 0kPa and increased to a pressure of maximum 1000kPa (30kPa/sec). These stimuli were applied in the suprascapular region, second toe, neck (with most severe pain, cases only), a non-painful site caudal to painful area of neck (5 cm caudal, cases only) and lower back.

Investigated population: 40 chronic neck pain patients and 300 control subjects

Results
Crude AUC: 1). Detection: site of the most severe pain at neck 0.93 (0.87–1.00). 2) Tolerance: site of the most severe pain at neck 0.87 (0.78–0.95). Fitted AUC: 1) Detection: site of the most severe pain at neck 0.94 (0.88–1.00) and 2) Tolerance: site of the most severe pain at neck 0.87 (0.78–0.97).

Results:
Pressure stimulation on the most severe pain sites is the most appropriate QST for distinguishing patients with chronic neck pain from asymptomatic control subjects.

Study 3

Method: pain pressure threshold and tolerance thresholds were measured using an electronic pressure algometer. Pressure started at 0kPa and increased to a pressure of maximum 1000kPa. These stimuli were applied in the suprascapular region, second toe, lower back (with most severe pain, cases only), a non-painful site cranial to painful area of lower back (5 cm cranial, cases only) and lower back in absence of painful area (for the controls).

Investigated population: 40 chronic low back pain patients and 300 control subjects

Results:

The best ranked tests with ROC curve >0.80 were the following:
- pressure pain threshold of suprascapular AUC (95% CI) of 0.80 (0.71-0.89) and sites of most severe pain in lower back AUC 0.87 (0.81-0.94)
- Tolerance threshold of most severe pain in lower back AUC (95%) 0.80 (0.71-0.89)

Pressure pain thresholds as measure of pain hypersensitivity had the highest ability in distinguishing chronic low back pain from control subjects.

For all studies:

Materials needed for measurement: (non)electronic pressure algometer (not electronic circa 200 euro). The manual algometer has proven to be as reliable as the computer controlled algometer (not yet published research René Castien).

Availability of materials needed for measurement: accessible for primary care

Burden on patient: medium burden on patient, patients were instructed to press a button once the stimulus became too uncomfortable

Time needed to apply the test: average of 5 to 10 minutes

Ability of an assistant or practice nurse to perform the test: yes

References

Study 1: P. de la Coba, S. Bruehl, M. Moreno-Padilla, G.A. Reyes Del Paso, Responses to Slowly Repeated Evoked Pain Stimuli in Fibromyalgia Patients: Evidence of Enhanced Pain Sensitization, Pain medicine (Malden, Mass.) 18(9) (2017) 1778-1786.

Study 2: A.Y. Neziri, A. Limacher, P. Juni, B.P. Radanov, O.K. Andersen, L. Arendt-Nielsen, M. Curatolo, Ranking of tests for pain hypersensitivity according to their discriminative ability in chronic neck pain, Regional anesthesia and pain medicine 38(4) (2013) 308-20.

Study 3: A.Y. Neziri, M. Curatolo, A. Limacher, E. Nuesch, B. Radanov, O.K. Andersen, L. Arendt-Nielsen, P. Juni, Ranking of parameters of pain hypersensitivity according to their discriminative ability in chronic low back pain, Pain 153(10) (2012) 2083-91.

1. QST: monofilaments (Von Frey/ Semmes Weinstein)

Background

Max von Frey used horse hair of various thickness to test sensibility. Later Semmes and Weinstein developed nylon variants. A normal testkit consists of 20 filaments, a mini set of 5 filaments has also been used.

Method

Allodynia, mechanical temporal summation and SREP (slowly repeated evoked pain) is assessed using monofilaments of different weights. To measure allodynia the monofilament is applied a few times and patients were asked to rate the pain. To measure mechanical temporal summation the monofilament is applied 30 times with a rate of 1/sec and the patients have to rate the pain.

To measure SREP the SREP series consists of 9 suprathreshold painful pressure stimuli 5 seconds in duration with an interstimulus interval of 30 seconds and patients have to rate the pain.

Investigated population

Fibromyalgia patients, rheumatoid arthritis patients, healthy controls

For all studies:

Materials needed for measurement: set of monofilaments

Availability of materials needed for measurement: easy to buy, a standardized set of 5 filaments is reliable enough and costs about 100 euro.

Burden on patient: low burden on patients

Time needed to apply the test: a filament is placed 1.5 sec. on the skin, kept for 1.5 sec on the skin and in 1.5 sec. removed. The filaments 1.65-4.08 must been placed 3x after each other, that is in one session to assess the pressure pain threshold. Than all filaments are 3x tested (so the first tested filaments are 9x tested). The monofilament has to be placed perpendicular to the skin and pressure must be carried out until the filament bows in a C. Total time 10-15 minutes.
Ability of an assistant or practice nurse to perform the test: easy to learn

Study 1:

Method: a standard mechanical TSP protocol (10 stimuli of 1-second duration at the thenar eminence using a 300-g monofilament with 1 second interstimulus interval) and the SREP protocol (9 suprathreshold pressure stimuli of 5-second duration applied to the fingernail with a 30-second interstimulus interval). To evaluate reliability for both protocols, they were repeated in a second session 4-7 days later.

To assess subjective pain intensity, a 10-cm visual analogue scale (VAS) was completed after each stimulus, with the anchors being “no pain” and “extremely painful.” The difference in pain assessment formats between the TSP and SREP protocols was necessitated by the different rates at which pain stimuli were presented in each. The standard TSP protocol uses a verbal NRS response format because of the need for rapid assessment after each pain stimulus (in our case, 1 pain rating/second). The SREP protocol is much slower and allows for the use of a written VAS (1 pain rating/30 seconds). For consistency with their originally published SREP protocol, the authors elected to maintain the VAS to assess the SREP pain stimuli, while using the required NRS format for pain assessment in the TSP protocol. To minimize any potential confounding influence of the different pain assessment formats and to insure that responses to both were as similar as possible, each participant received instructions and training in use of both NRS and VAS ratings before the primary study procedures

Investigated population: Thirty-five fibromyalgia (FM) patients and 30 rheumatoid arthritis (RA) patients completed, in pseudorandomized order

Results: Evidence for significant pain sensitization over trials (increasing pain intensity ratings) was observed for SREP in FM (p < .001) but not in RA (p = .35), whereas significant sensitization was observed in both diagnostic groups for the TSP protocol (p < .008). Compared with TSP, SREP demonstrated higher overall diagnostic accuracy (87.7% versus 64.6%), greater sensitivity (0.89 versus 0.57), and greater specificity (0.87 versus 0.73) in discriminating between FM and RA patients. Test-retest reliability of SREP sensitization was good in FM (intraclass correlations = 0.80), and moderate in RA (intraclass correlations = 0.68).

Study 2

Method: A SREP protocol was administered to all subjects, consisting of a single series of nine low-intensity pressure stimuli of five-second duration and thirty-second interstimulus interval. Subjective evoked pain intensity was assessed with a visual analogical scale. Clinical fibromyalgia pain was assessed with the McGill Pain Questionnaire.

Investigated population: twenty-four fibromyalgia patients and 24 healthy participants

Results: perceived pain intensity increased during the SREP protocol in fibromyalgia patients but not in healthy participants. Neither pain threshold nor pain tolerance was associated with SREP. Degree of SREP sensitization was associated with McGill Pain Questionnaire-Sensory ratings of fibromyalgia pain. The effect size for differences between the fibromyalgia and healthy control groups was greater, and the overlaps of the groups distributions lower, for SREP sensitization than for traditional evoked pain measures of pain threshold and tolerance. SREP demonstrated higher specificity in discriminating fibromyalgia and control groups relative to pain threshold or tolerance.

References:

Study 1: P. de la Coba, S. Bruehl, M. Moreno-Padilla, G.A. Reyes Del Paso, Responses to Slowly Repeated Evoked Pain Stimuli in Fibromyalgia Patients: Evidence of Enhanced Pain Sensitization. Pain Medicine (Malden, Mass.) 18(9) (2017) 1778-1786.

Study 2: P. de la Coba, S. Bruehl, C.M. Galvez-Sanchez, G.A. Reyes Del Paso, Slowly Repeated Evoked Pain as a Marker of Central Sensitization in Fibromyalgia: Diagnostic Accuracy and Reliability in Comparison With Temporal Summation of Pain. Psychosom Med 80(6) (2018) 573-580.

1. Clothes peg

Background

A clothes peg can be used to measure pain sensitivity. Clothes pegs can have different clamping forces which can be calibrated.

Method

A calibrated clothes peg was applied for 10 seconds and patients rated the pain intensity on a 0 to 10 numerical rating scale. Pressure pain detection threshold (PPdt) and pressure pain tolerance threshold (PPtt) were measured with a standard electronic algometer. Both methods were performed on both middle fingers and ear lobes.

To measure pressure pain detection threshold on the middle finger pressure is gradually increased until the pressure is considered painful, this was performed 3 times. To measure pressure pain tolerance threshold was measured applying the algometer to the middle finger of the dominant hand until maximally tolerable pain level, this was performed one time.

The pressure of the clothes peg on the middle finger is considered to be beneath or slightly above the pressure pain threshold. The pressure pain of the clothes peg on the earlobe is perceived as consistently and clearly above the pain threshold and assesses the ability of the patient to endure pain. Therefore, clothes peg exposure tests integrate aspects of pain sensitivity which are otherwise tested separately.

Investigated population

We tested 157 in-patients with different pain types (orthopedic, psychosomatic). In a subgroup of 47 patients repeatability (test-retest reliability) was calculated.

Materials needed for measurement: calibrated clothes peg

Availability of materials needed for measurement: clothes peg cheap, equipment to calibrate the clothes peg needed. In this study clothes pegs were used with a clamping force of 10 Newton at an extension of 5 mm.

Burden on patient: low to medium burden on patients

Time needed to apply the test: 15 minutes

Ability of an assistant or practice nurse to perform the test: easy to learn

Results

Clothes peg values correlate at a clinically meaningful level with pressure pain detection tresholds (PPdt) and pressure pain tolerance thresholds (PPtt) measured by an electronic algometer.

Clothes peg values correlated with PPdt values for finger testing with r = -0.54 and for earlobe testing with r = -0.55 (all p-values < 0.001). Clothes peg values also correlated with PPtt values for finger testing with r = -0.55 (p < 0.001). R (rho) is the spearmans rank correlation coefficient. According to Cohen’s conventions, r=0.3-0.5 corresponds to a correlation size of a medium effect and r>0.5 corresponds to a large effect.

Test-retest reliability (repeatability) showed equally stable results for clothes peg algometry and the electronic algometer (all r-values > 0.89, all p-values < 0.001). Conclusions: Information on pain sensitivity provided by a calibrated clothes peg and an established algometer correlate at a clinically meaningful level.

**Pain characteristics of the two clinical groups**. Our aim was to compare the two algometric test methods in a wide range of pain types. Therefore we recruited patients from the orthopaedic department and the medical-psychosomatic department. **The left figure** illustrates the distribution of the baseline pain values (NRS) in both groups. **The right figure**illustrates the distribution of the pain sensitivity values (NRS) of the ear lobe provoked by clothes pegs. The box-and-whisker-plots show the median with interquartile range (box: 25^th^and 75^th^percentile) and 5^th^and 95th percentile (whiskers) of the data distribution.

References:

N. Egloff, N. Klingler, R. von Kanel, R.J. Camara, M. Curatolo, B. Wegmann, E. Marti, M.L. Ferrari, Algometry with a clothes peg compared to an electronic pressure algometer: a randomized cross-sectional study in pain patients. BMC Musculoskelet Disord 12 (2011) 174

1. CPM: combination of ischemic stimuli and PPT

Background:

Conditioned pain modulation (CPM) means that a test-stimulus and a conditioning stimulus are used together in the test. A combination of ischemic stimuli and PPTs is used here as CPM. In healthy controls the conditioning stimulus leads to a reduction in the perceived intensity of the test-stimulus. In central sensitization there is a smaller reduction of the perceived intensity of the test-stimulus due to reduced inhibition of descending control.

Study 1
Method: in comfortable recumbent position CPM is evaluated by conditioning tonic stimulation of pain using ischemic compression (with a tourniquet cuff) on the left arm until pain was rated as 4cm on e-VAS and PPT from peripatellar region.

Investigated population: 17 knee osteoarthritis patients

Results multiple-regression model: 55% of variance in peak pain intensity in KOA patients (p=0.001). Significant correlations (P < 0.05): PPTs by handheld pressure algometry in peripatellar region vs. TA (R = 0.94), PPTs by computer controlled pressure algometry vs. handheld pressure algometry in peripatellar region (R = 0.71), PPTs by computer-controlled pressure algometry in peripatellar region vs. handheld pressure algometry on TA (R = 0.71) and temporal summation at the knee vs. TA (R = 0.73).

Study 2
Method: CPM was induced by inflating an occlusion cuff at the subject’s left arm to a painful intensity. The occlusion cuff was inflated at a rate of 20 mmHg/s until ‘the first sensation of pain’ and maintained for 30 s. Afterwards, pain intensity, as a result of cuff inflation, was rated on a numerical rating scale (0 = no to 10 = worst possible pain). Next, cuff inflation was increased or decreased until pain intensity at left arm was rated as 3/10. TS assessment was then repeated during maintenance of the cuff inflation.

TS was provoked by means of 10 consecutive pulses at previously determined pressure pain threshold at each location. TS started 2 min after pressure pain threshold measurement. For each pulse, pressure was gradually increased at a rate of 2 kg/s to the determined pressure pain threshold and maintained for 1 s before being released (1 s interstimulus interval). Pain intensity of first, fifth, and 10th pulse was rated on a numerical rating scale (0 = no to 10 = worst possible pain). Afterwards, a rest period of 5 min was allowed.

Investigated population: 35 chronic patients with whiplash associated disorder (WAD), 31 healthy controls

Results: TS of pressure pain was significantly depleted among healthy controls. In contrast, TS was quite similar prior to and during cuff inflation in chronic WAD, providing evidence for dysfunctional CPM in patients with chronic WAD. The present study demonstrates a lack of endogenous pain inhibitory pathways, and in particularly CPM, in patients with chronic WAD, and hence provides additional evidence for the presence of central sensitization in chronic WAD.

Study 3
Method: pressure pain modulation by heterotopic descending noxious inhibitory control (DNIC), ischemic compression of the arm. A 7.5cm wide tourniquet cuff (VBM, Germany) was wrapped around the left arm. The lower rim of the tourniquet cuff was at 3cm proximal to the cubital fossa. The cuff control unit (Aalborg University, Denmark) was programmed to maintain the pressure at 36kPa (above the systolic pressure). After the target pressure was reached, the patient was asked to repeat hand grip for 10 times or more until 4 on the VAS was reached. The patients rated the contraction-evoked pain on an electronic VAS on which “0” represented “no pain” and “10” represented “maximal pain”. When “4” on VAS was reached, PPTs on all test sites and control sites were assessed. The cuff was released once PPT assessments were finished. PPT assessment was repeated 5min after cuff-evoked pain subsided.

Investigated population: 48 pts knee OA, 2 groups 24 VAS >6 ,24<6, 24 controls

Results: There were no significant increases in PPT at the peripatellar region during cuff stimulation in neither group A nor group B. PPT increased significantly during cuff stimulation in controls (ANOVA: F_2,146_=6.1, p<0.01; SNK: p<0.05). Significant increase of PPT during cuff stimulation was found at TA in groups A, B and controls and at forearm in group A and controls (ANOVA: F_2,146_=5.6, p<0.01; SNK: p<0.05;). Patients with osteo-arthrosis had a significant facilitation of temporal summation from both the knee and tibialis anterior, significantly less DNIC as compared with controls.

References:

Study 1: S.T. Skou, T. Graven-Nielsen, L. Lengsoe, O. Simonsen, M.B. Laursen, L. Arendt-Nielsen, Relating clinical measures of pain with experimentally assessed pain mechanisms in patients with knee osteoarthritis, Scandinavian journal of pain 4(2) (2013) 111-117.

Study 2: L. Daenen, J. Nijs, N. Roussel, K. Wouters, M. Van Loo, P. Cras, Dysfunctional pain inhibition in patients with chronic whiplash-associated disorders: An experimental study, Clinical rheumatology 32(1) (2013) 23-31.

Study 3: L. Arendt-Nielsen, H. Nie, M.B. Laursen, B.S. Laursen, P. Madeleine, O.H. Simonsen, T. Graven-Nielsen, Sensitization in patients with painful knee osteoarthritis, Pain 149(3) (2010) 573-81.

1. Sensory hypersensitivity scale

The Sensory Hypersensitivity Scale (SHS) is a 25-item self-report measure of sensory hypersensitivity. The SHS assesses both general sensitivity and modalityspecific sensitivity (e.g. touch, taste, and hearing). The aim of the development of this tool was to focus on the sensory aspects of hypersensitivity, largely independent from psychological constructs of depression and anxiety.

Five studies were performed to validatie this questionnaire with 1202 participants (157 individuals with chronic pain), The SHS demonstrated an adequate overall internal reliability (Cronbach’s alpha) of 0.81, suggesting the tool can be used as a cross-modality assessment of sensitivity. SHS scores demonstrated only modest correlations (Pearson’s r) with depressive symptoms (0.19) and anxiety (0.28), suggesting a low level of overlap with psychiatric complaints. Overall SHS scores showed significant but relatively modest correlations (Pearson’s r) with three measures of sensory testing: cold pain tolerance (−0.34); heat pain tolerance (−0.285); heat pain threshold (−0.271). Women reported significantly higher scores on the SHS than did men, although gender-based differences were small. In a chronic pain sample, individuals with fibromyalgia syndrome demonstrated significantly higher SHS scores than did individuals with osteoarthritis or back pain. The SHS appears suitable as a screening measure for sensory hypersensitivity, though additional research is warranted to determine its suitability as a proxy for central sensitization.

Materials needed for measurement: test on paper or online (not yet available)
Availability of materials needed for measurement: test not available online, only from the research article

Burden on patient: light, answering 25 questions on a 5-point scale

Time needed to apply the test: 10 minutes to fill in, 5 minutes to score

Ability of an assistant or practice nurse to perform the test: easy

The Sensory Hypersensitive Scale (Likert scale 1-5):

I suffer from allergies

I am allergy-free

I have a number of allergies

I often feel too hot in an environment where others don’t seem to be bothered

I am easily disturbed by high temperatures

I often feel too cold in an environment where others don’t seem to be bothered

I am easily disturbed by low temperatures

My eyes are sensitive to sunlight

I am sensitive to bright light

I am not really bothered by bright lights

I am quite sensitive to pain

I can tolerate a large amount of pain

Things that would ordinarily hurt others are not painful to me

I often react to odors that other do not initially notice

I seem to notice smells that other people do not

I rarely notice smells

When I read, it must be totally quiet

I cannot study or read if there is any conversation or noise around

I can work even in noisy circumstances

I tend to be a picky eater

There are many foods that taste bad to me

I can eat almost anything

I am generally unable to wear clothes made of rough material

I am sensitive to rough textures

I can wear almost any kind of fabric without it bothering me

References:

E.A. Dixon, G. Benham, J.A. Sturgeon, S. Mackey, K.A. Johnson, J. Younger, Development of the Sensory Hypersensitivity Scale (SHS): a self-report tool for assessing sensitivity to sensory stimuli, Journal of Behavioral Medicine 39(3) (2016) 537-50.

Appendix 1: measurement instruments from our systematic review

| Measurement instrument | What is measured? | Examples |
| --- | --- | --- |
| Quantitative sensory testing (QST) | Hyperalgesia, allodynia, temporal summation | Thermal stimuli: thresholds for cold pain, heat pain, cold detection and heat detection; e.g., putting the hand in an iced water bath  Tactile stimuli: pressure pain thresholds (PPTs)  Vibratory or vibrotactile stimuli: detection thresholds for vibration or combination of tactile and vibratory stimuli, e.g., electric toothbrush  Electrical stimuli: reaction to electrical pulses with electrodes  Distention: distending the rectum or oesophagus with an inflatable balloon  Ischemic stimuli: ischemic compression of the arm with a cuff  Reaction on specific pain mediators, e.g. reaction on injection with hypertonic saline |
| Two different quantitative sensory tests together | Conditioned pain modulation (CPM) | Tonic phasic stimulation: phasic heat test with counter irritation of cold [  Ischemic stimulation: inflating an occlusion cuff, comparing pressure pain prior to and during cuff inflation  The nociception withdrawal reflex e.g. H(offman) reflex: stimulation of median nerve with an EMG device, measurement of H wave (a compound muscle action potential)  Measurement of the cutaneous silent period (CSP): a brief pause in muscle action potentials following strong stimulation of a cutaneous nerve during a sustained voluntary contraction |
| MRI*, fMRI*, PET*, somatosensory evoked potentials (SEP) | Structural and functional brain changes | Measurement of changes in brain morphology (global and regional gray matter volumes), changes in density and changes in signaling |
| Measurement of cytokine levels | Laboratory evaluation | Measurement of serum levels of pro-inflammatory interleukines (Il-1, IL-6, IL-8) and anti-inflammatory interleukines (IL-4, IL-10); serum levels of TNF-alpha, a pro-inflammatory cytokine |
| Measurement of neurotrophin levels | Laboratory evaluation | Measurement of serum levels of nerve growth factor (NGF) and brain derived neurotrophic factor (BDNF) |
| Questionnaires | Symptoms, history of functional syndromes | Central sensitization Inventory (CSI) |
|  | Sensory aspects of hypersensitivity | Sensory Hypersensitivity Scale (SHS) |

Search strategy Delphi procedure

We selected 20 studies from our systematic review.

We conducted additional searches in PubMed, the search was conducted per measurement instrument category.

Search terms:

“Central Nervous System Sensitization”[Mesh]

“Central Sensitization” OR “Central Sensitisation” OR “Central Nervous System Sensitization” OR “Central Nervous System Sensitisation”.

AND

(all the following search terms were combined individually with abovementioned search terms)

Thermal stimulation

Tactile stim* OR “Pressure pain threshold*”

Vibratory OR vibrotactile stim*

Electrical stimuli

Ischemic stimulat*

Tonic phasic stimulation

Nociception withdrawal reflex OR H reflex OR Hoffman reflex

Cutaneous silent period

Cytokine*

Neurotrophin* OR Nerve growth factor OR NGF OR Brain derived neurotrophic factor (BDNF)

Central sensitization Inventory OR CSI

Sensory Hypersensitivity Scale OR SHS

Filter: humans

We wanted to select measurement instruments, which are suitable for use in general practice. It is important to know:

1. The properties of the tests: background, methods and costs, investigated population, diagnostic test performance and the results.
2. How the test must be carried out: availability of instruments or materials needed, time needed to perform the test, burden on patient and possibility of an assistant performing the test.

This information and an overview of the corresponding empirical evidence will be provided for all the instruments.

We excluded publications that are not focussing on CS, written in other languages than English, German, French or Dutch and publications that report on animal studies. Two authors (CdB and CG) screened the search results and independently selected publications based on title and abstract. Both authors only included selected publications that are available as full text. These publications were discussed together.
In case the search provided too many publications (>100), the following search term was added:
AND specif*

Figure 3: Flowchart search first round (first 12 instruments)


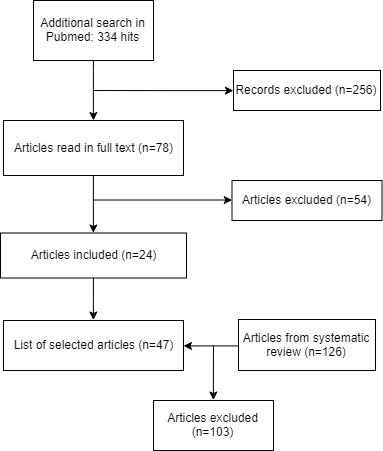


Vibratory or vibrotactile stimuli

From systematic review: 1 [14]

Additional search terms: ((“Central Sensitization” OR “Central Sensitisation” OR “Central Nervous System Sensitization” OR “Central Nervous System Sensitisation” OR “Central Nervous System Sensitization”[Mesh])) AND (Vibratory OR vibrotactile stim*)

Hits: 5

Full text read: 2

Included: 1 [15]

Thermal stimuli

From systematic review: 0

Additional search terms: ((((“Central Nervous System Sensitization”[Mesh] OR “Central Sensitization” OR “Central Sensitisation” OR “Central Nervous System Sensitization” OR “Central Nervous System Sensitisation”)) AND (Thermal stimulation OR Thermal)))

114 hits, added specificity: 6 hits

Full text read: 3

Included: 2 [16, 17]

Tactile stimuli

From systematic review: 1 [18]

Additional search terms: (((((((“Central Nervous System Sensitization”[Mesh] OR “Central Sensitization” OR “Central Sensitisation” OR “Central Nervous System Sensitization” OR “Central Nervous System Sensitisation”))))) AND (tactile stim* OR pressure pain thresholds OR PPT OR pressure pain threshold)))

187 hits, added specificity: 7 hits

Full text read: 5

Included: 2 [19, 20]

Electrical stimuli

From systematic review: 1 [17]

Additional search terms: (((((((“Central Nervous System Sensitization”[Mesh] OR “Central Sensitization” OR “Central Sensitisation” OR “Central Nervous System Sensitization” OR “Central Nervous System Sensitisation”))))))) AND Electrical stimuli

Hits: 38

Full text read: 2

Included: 1 [21]

Ischemic stimuli

From systematic review: 0

Additional search terms: (((((“Central Nervous System Sensitization”[Mesh] OR “Central Sensitization” OR “Central Sensitisation” OR “Central Nervous System Sensitization” OR “Central Nervous System Sensitisation”))))) AND (ischemic OR ischemic stim*)

Hits: 18

Full text read: 3

Included: 0

CPM: Tonic phasic stimulation

From systematic review: 2 [22, 23]

Additional search terms: ((((((“Central Nervous System Sensitization”[Mesh] OR “Central Sensitization” OR “Central Sensitisation” OR “Central Nervous System Sensitization” OR “Central Nervous System Sensitisation”))))) AND (Tonic phasic stimulation OR tonic OR phasic OR counterirritation))

Hits: 26

Full text read: 6

Included: 1 [24]

CPM: ischemic stimulus and pressure point thresholds (PPT)

From systematic review: 2 [25, 26]

Additional search terms: : (((((“Central Nervous System Sensitization”[Mesh] OR “Central Sensitization” OR “Central Sensitisation” OR “Central Nervous System Sensitization” OR “Central Nervous System Sensitisation”))))) AND (ischemic OR ischemic stim*)

Hits: 18

Full text read: 3

Included: 0

The nociception withdrawal reflex (NFR)

From systematic review: 1 [17]

Additional search terms: ((((((“Central Nervous System Sensitization”[Mesh] OR “Central Sensitization” OR “Central Sensitisation” OR “Central Nervous System Sensitization” OR “Central Nervous System Sensitisation”))))) AND (The nociception withdrawal reflex OR NFR OR H-reflex OR Hoffman)

Hits: 18

Full text read: 8

Included: 1[27]

The cutaneous silent period (CSP)

From systematic review: 2 [28, 29]

Additional search terms: (((((“Central Nervous System Sensitization”[Mesh] OR “Central Sensitization” OR “Central Sensitisation” OR “Central Nervous System Sensitization” OR “Central Nervous System Sensitisation”))))) AND (cutaneous silent period OR CSP)

Hits 7

Full text read: 3

Included: 1[30]

Cytokines: TNF-alpha, pro-inflammatory (IL-1, IL-6, IL-8) and anti-inflammatory (IL-4, Il-10)

From systematic review: 2 [31, 32]

Additional search terms: ((((((((“Central Nervous System Sensitization”[Mesh] OR “Central Sensitization” OR “Central Sensitisation” OR “Central Nervous System Sensitization” OR “Central Nervous System Sensitisation”))))) AND ((TNF)))) OR (interleukins AND cytokin*))

Hits: 19

Full text read: 12

Included: 2 [33, 34]

NGF and BDNF

From systematic review: 3 [3, 31, 35]

Search terms: (("Central Nervous System Sensitization"[Mesh] OR "Central Sensitization" OR "Central Sensitisation" OR "Central Nervous System Sensitization" OR central nervous system sensitisation)) AND ((NGF OR nerve growth factor) OR (BDNF OR brain derived neurotrophic factor))

Hits: 88

Full text read: 12

Included: 2 [36]

Central sensitization inventory

From systematic review: 7 [37-43]

Search terms: (((("Central Nervous System Sensitization"[Mesh] OR "Central Sensitization" OR "Central Sensitisation" OR "Central Nervous System Sensitization" OR central nervous system sensitisation)))) AND central sensitization inventory

Hits: 69

Full text reading: 19

Included: 11 [44-54]

Sensory hypersensitivity scale (SHS)

From systematic review: [55]

Search terms: (("Central Nervous System Sensitization"[Mesh] OR "Central Sensitization" OR "Central Sensitisation" OR "Central Nervous System Sensitization" OR central nervous system sensitisation)) AND sensory hypersensitivity scale

15 hits

Full text reading: 0

The SHS is not available, only in the article it is mentioned, therefore it was excluded in the first round.

Search strategy additional three measurement instruments:

Sensory hypersensitivity scale

From systematic review: 1 [55]

Search terms: (("Central Nervous System Sensitization"[Mesh] OR "Central Sensitization" OR "Central Sensitisation" OR "Central Nervous System Sensitization" OR central nervous system sensitisation)) AND sensory hypersensitivity scale

15 hits

Full text reading: 0

Selected: 0

Monofilaments

From systematic review: 0

Search terms: (("Central Nervous System Sensitization"[Mesh] OR "Central Sensitization" OR "Central Sensitisation" OR "Central Nervous System Sensitization" OR central nervous system sensitisation)) AND monofilaments

6 hits

Full text reading: 4

Selected: 0

Additional search terms:

From systematic review: 0

((“Central Sensitization” OR “Central Sensitisation” OR “Central Nervous System Sensitization” OR “Central Nervous System Sensitisation” OR “Central Nervous System Sensitization”[Mesh])) AND temporal summation AND specif*

Hits: 17

Full text read: 5

Included: 2 ([18, 56]

Clothes peg

From systematic review: 0

Search terms: clothes peg

Hits: 71

Full text read: 1

Selected: 1 [57]

References

[1] C.J. Woolf, Evidence for a central component of post-injury pain hypersensitivity, Nature 306(5944) (1983) 686-8.

[2] B. Walitt, M. Čeko, J.L. Gracely, R.H. Gracely, Neuroimaging of central sensitivity syndromes: Key insights from the scientific literature, Curr. Rheumatol. Rev. 12(1) (2016) 55-87.

[3] S.A. Zanette, J.A. Dussan-Sarria, A. Souza, A. Deitos, I.L. Torres, W. Caumo, Higher serum S100B and BDNF levels are correlated with a lower pressure-pain threshold in fibromyalgia, Mol. Pain 10 (2014) 46.

[4] IASP taxonomy, 2018. <https://www.iasp-pain.org/Taxonomy>.

[5] P. Hansson, Translational aspects of central sensitization induced by primary afferent activity: What it is and what it is not, Pain 155(10) (2014) 1932-1934.

[6] C.J. Woolf, Central sensitization: implications for the diagnosis and treatment of pain, Pain 152(3 Suppl) (2011) S2-15.

[7] S. Kaya, L. Hermans, T. Willems, N. Roussel, M. Meeus, Central sensitization in urogynecological chronic pelvic pain: A systematic literature review, Pain Physician 16(4) (2013) 291-308.

[8] N. Stabell, A. Stubhaug, T. Flægstad, E. Mayer, B.D. Naliboff, C.S. Nielsen, Widespread hyperalgesia in adolescents with symptoms of irritable bowel syndrome: Results from a large population-based study, The Journal of Pain 15(9) (2014) 898-906.

[9] J. Nijs, M. Meeus, J. van Oosterwijck, K. Ickmans, G. Moorkens, G. Hans, L.S. de Clerck, In the mind or in the brain? Scientific evidence for central sensitisation in chronic fatigue syndrome, Eur. J. Clin. Invest. 42(2) (2012) 203-212.

[10] L.D. C. den Boer, B. Terluin, J.C. van der Wouden, A. H. Blankenstein, C. P. van Wilgen, P. Lucassen, H. E. van der Horst, Central sensitization in chronic pain and medically unexplained symptom research: A systematic review of definitions, operationalizations and measurement instruments, J. Psychosom. Res. 117(2 (2019)) 32-40.

[11] J. Jones, D. Hunter, Consensus methods for medical and health services research, BMJ 311(7001) (1995) 376-80.

[12] O.R. Maarsingh, J. Dros, H.C. van Weert, F.G. Schellevis, P.J. Bindels, H.E. van der Horst, Development of a diagnostic protocol for dizziness in elderly patients in general practice: a Delphi procedure, BMC Fam. Pract. 10 (2009) 12.

[13] R. Meijer, D. Ihnenfeldt, M. Vermeulen, R. De Haan, J. Van Limbeek, The use of a modified Delphi procedure for the determination of 26 prognostic factors in the sub-acute stage of stroke, Int. J. Rehabil. Res. 26(4) (2003) 265-70.

[14] D.R. Nixdorf, A. Hemmaty, J.O. Look, E.L. Schiffman, M.T. John, Electric toothbrush application is a reliable and valid test for differentiating temporomandibular disorders pain patients from controls, BMC Musculoskelet. Disord. 10 (2009) 94.

[15] L.B. Campi, P.C. Jordani, H.L. Tenan, C.M. Camparis, D.A. Goncalves, Painful temporomandibular disorders and central sensitization: implications for management-a pilot study, Int. J. Oral Maxillofac. Surg. 46(1) (2017) 104-110.

[16] R. Staud, E.E. Weyl, D.D. Price, M.E. Robinson, Mechanical and heat hyperalgesia highly predict clinical pain intensity in patients with chronic musculoskeletal pain syndromes, J. Pain 13(8) (2012) 725-35.

[17] J.A. Desmeules, C. Cedraschi, E. Rapiti, E. Baumgartner, A. Finckh, P. Cohen, P. Dayer, T.L. Vischer, Neurophysiologic evidence for a central sensitization in patients with fibromyalgia, Arthritis Rheum. 48(5) (2003) 1420-9.

[18] P. de la Coba, S. Bruehl, M. Moreno-Padilla, G.A. Reyes Del Paso, Responses to Slowly Repeated Evoked Pain Stimuli in Fibromyalgia Patients: Evidence of Enhanced Pain Sensitization, Pain Med. 18(9) (2017) 1778-1786.

[19] A.Y. Neziri, A. Limacher, P. Juni, B.P. Radanov, O.K. Andersen, L. Arendt-Nielsen, M. Curatolo, Ranking of tests for pain hypersensitivity according to their discriminative ability in chronic neck pain, Reg. Anesth. Pain Med. 38(4) (2013) 308-20.

[20] A.Y. Neziri, M. Curatolo, A. Limacher, E. Nuesch, B. Radanov, O.K. Andersen, L. Arendt-Nielsen, P. Juni, Ranking of parameters of pain hypersensitivity according to their discriminative ability in chronic low back pain, Pain 153(10) (2012) 2083-91.

[21] J. Van Oosterwijck, J. Nijs, M. Meeus, L. Paul, Evidence for central sensitization in chronic whiplash: a systematic literature review, Eur. J. Pain 17(3) (2013) 299-312.

[22] J.B. Correa, L.O. Costa, N.T. de Oliveira, K.A. Sluka, R.E. Liebano, Central sensitization and changes in conditioned pain modulation in people with chronic nonspecific low back pain: a case-control study, Exp. Brain Res. 233(8) (2015) 2391-9.

[23] J.S. Heymen, Central processing of noxious stimuli in patients with irritable bowel syndrome compared to healthy controls, ProQuest Information & Learning, US, 2007, pp. 1976-1976.

[24] S.T. Skou, T. Graven-Nielsen, L. Lengsoe, O. Simonsen, M.B. Laursen, L. Arendt-Nielsen, Relating clinical measures of pain with experimentally assessed pain mechanisms in patients with knee osteoarthritis, Scand J Pain 4(2) (2013) 111-117.

[25] L. Arendt-Nielsen, H. Nie, M.B. Laursen, B.S. Laursen, P. Madeleine, O.H. Simonsen, T. Graven-Nielsen, Sensitization in patients with painful knee osteoarthritis, Pain 149(3) (2010) 573-81.

[26] L. Daenen, J. Nijs, N. Roussel, K. Wouters, M. Van Loo, P. Cras, Dysfunctional pain inhibition in patients with chronic whiplash-associated disorders: An experimental study, Clin. Rheumatol. 32(1) (2013) 23-31.

[27] V. Skljarevski, N.M. Ramadan, The nociceptive flexion reflex in humans -- review article, Pain 96(1-2) (2002) 3-8.

[28] S.H. Baek, H.Y. Seok, Y.S. Koo, B.J. Kim, Lengthened Cutaneous Silent Period in Fibromyalgia Suggesting Central Sensitization as a Pathogenesis, PLoS One 11(2) (2016) e0149248.

[29] O. Kilinc, S. Sencan, T. Ercalik, P.K. Koytak, H. Alibas, O.H. Gunduz, T. Tanridag, K. Uluc, Cutaneous silent period in myofascial pain syndrome, Muscle Nerve 57(1) (2018) E24-e28.

[30] O. Sahin, S. Yildiz, N. Yildiz, Cutaneous silent period in fibromyalgia, Neurol. Res. 33(4) (2011) 339-43.

[31] A. Deitos, J.A. Dussan-Sarria, A. Souza, L. Medeiros, G. Tarrago Mda, F. Sehn, M. Chassot, S. Zanette, A. Schwertner, F. Fregni, I.L. Torres, W. Caumo, Clinical Value of Serum Neuroplasticity Mediators in Identifying the Central Sensitivity Syndrome in Patients With Chronic Pain With and Without Structural Pathology, Clin. J. Pain 31(11) (2015) 959-67.

[32] E. Generaal, N. Vogelzangs, G.J. Macfarlane, R. Geenen, J.H. Smit, J. Dekker, B.W. Penninx, Basal inflammation and innate immune response in chronic multisite musculoskeletal pain, Pain 155(8) (2014) 1605-12.

[33] F. Euteneuer, M.J. Schwarz, A. Hennings, S. Riemer, T. Stapf, V. Selberdinger, W. Rief, Psychobiological aspects of somatization syndromes: contributions of inflammatory cytokines and neopterin, Psychiatry Res. 195(1-2) (2012) 60-5.

[34] H. Wang, M. Schiltenwolf, M. Buchner, The role of TNF-alpha in patients with chronic low back pain-a prospective comparative longitudinal study, Clin. J. Pain 24(3) (2008) 273-8.

[35] W. Caumo, A. Deitos, S. Carvalho, J. Leite, F. Carvalho, J.A. Dussán-Sarria, M.G.L. Tarragó, A. Souza, I.L.S. Torres, F. Fregni, Motor cortex excitability and BDNF levels in chronic musculoskeletal pain according to structural pathology, Front. Hum. Neurosci. 10(2016JULY) (2016).

[36] H. Wang, M. Buchner, M.T. Moser, V. Daniel, M. Schiltenwolf, The role of IL-8 in patients with fibromyalgia: a prospective longitudinal study of 6 months, Clin. J. Pain 25(1) (2009) 1-4.

[37] J. Kregel, P.J. Vuijk, F. Descheemaeker, D. Keizer, R. van der Noord, J. Nijs, B. Cagnie, M. Meeus, P. van Wilgen, The Dutch Central Sensitization Inventory (CSI): Factor Analysis, Discriminative Power, and Test-Retest Reliability, Clin. J. Pain 32(7) (2016) 624-30.

[38] T.G. Mayer, R. Neblett, H. Cohen, K.J. Howard, Y.H. Choi, M.J. Williams, Y. Perez, R.J. Gatchel, The development and psychometric validation of the central sensitization inventory, Pain Pract 12(4) (2012) 276-85.

[39] R. Neblett, M.M. Hartzell, H. Cohen, T.G. Mayer, M. Williams, Y. Choi, R.J. Gatchel, Ability of the central sensitization inventory to identify central sensitivity syndromes in an outpatient chronic pain sample, Clin. J. Pain 31(4) (2015) 323-32.

[40] R. Neblett, M.M. Hartzell, T.G. Mayer, H. Cohen, R.J. Gatchel, Establishing Clinically Relevant Severity Levels for the Central Sensitization Inventory, Pain Pract 17(2) (2017) 166-175.

[41] R. Neblett, H. Cohen, Y. Choi, M.M. Hartzell, M. Williams, T.G. Mayer, R.J. Gatchel, The Central Sensitization Inventory (CSI): establishing clinically significant values for identifying central sensitivity syndromes in an outpatient chronic pain sample, J. Pain 14(5) (2013) 438-45.

[42] T. Scerbo, J. Colasurdo, S. Dunn, J. Unger, J. Nijs, C. Cook, Measurement Properties of the Central Sensitization Inventory: A Systematic Review, Pain Pract 18(4) (2018) 544-554.

[43] C.P. van Wilgen, P.J. Vuijk, J. Kregel, L. Voogt, M. Meeus, F. Descheemaeker, D. Keizer, J. Nijs, Psychological Distress and Widespread Pain Contribute to the Variance of the Central Sensitization Inventory: A Cross-Sectional Study in Patients with Chronic Pain, Pain Pract 18(2) (2018) 239-246.

[44] A. Chiarotto, C. Viti, A. Sulli, M. Cutolo, M. Testa, D. Piscitelli, Cross-cultural adaptation and validity of the Italian version of the Central Sensitization Inventory, Musculoskelet Sci Pract 37 (2018) 20-28.

[45] K. Tanaka, T. Nishigami, A. Mibu, M. Manfuku, S. Yono, Y. Shinohara, A. Tanabe, R. Ono, Validation of the Japanese version of the Central Sensitization Inventory in patients with musculoskeletal disorders, PLoS One 12(12) (2017) e0188719.

[46] R. Neblett, T.G. Mayer, The Central Sensitization Inventory (CSI): some background and current trends, Spine J 17(11) (2017) 1766-1767.

[47] J. Gervais-Hupe, J. Pollice, J. Sadi, L.C. Carlesso, Validity of the central sensitization inventory with measures of sensitization in people with knee osteoarthritis, Clin. Rheumatol. 37(11) (2018) 3125-3132.

[48] T. Nishigami, K. Tanaka, A. Mibu, M. Manfuku, S. Yono, A. Tanabe, Development and psychometric properties of short form of central sensitization inventory in participants with musculoskeletal pain: A cross-sectional study, PLoS One 13(7) (2018) e0200152.

[49] R.A. Coronado, S.Z. George, The Central Sensitization Inventory and Pain Sensitivity Questionnaire: An exploration of construct validity and associations with widespread pain sensitivity among individuals with shoulder pain, Musculoskelet Sci Pract 36 (2018) 61-67.

[50] J. Kregel, C. Schumacher, M. Dolphens, A. Malfliet, D. Goubert, D. Lenoir, B. Cagnie, M. Meeus, I. Coppieters, Convergent Validity of the Dutch Central Sensitization Inventory: Associations with Psychophysical Pain Measures, Quality of Life, Disability, and Pain Cognitions in Patients with Chronic Spinal Pain, Pain Pract 18(6) (2018) 777-787.

[51] W. Caumo, L.C. Antunes, J.L. Elkfury, E.G. Herbstrith, R. Busanello Sipmann, A. Souza, I.L. Torres, V. Souza Dos Santos, R. Neblett, The Central Sensitization Inventory validated and adapted for a Brazilian population: psychometric properties and its relationship with brain-derived neurotrophic factor, J. Pain Res. 10 (2017) 2109-2122.

[52] A. Knezevic, R. Neblett, M. Jeremic-Knezevic, S. Tomasevic-Todorovic, K. Boskovic, P. Colovic, A. Cuesta-Vargas, Cross-Cultural Adaptation and Psychometric Validation of the Serbian Version of the Central Sensitization Inventory, Pain Pract 18(4) (2018) 463-472.

[53] R. Neblett, M.M. Hartzell, M. Williams, K.R. Bevers, T.G. Mayer, R.J. Gatchel, Use of the Central Sensitization Inventory (CSI) as a treatment outcome measure for patients with chronic spinal pain disorder in a functional restoration program, Spine J 17(12) (2017) 1819-1829.

[54] A.I. Cuesta-Vargas, C. Roldan-Jimenez, R. Neblett, R.J. Gatchel, Cross-cultural adaptation and validity of the Spanish central sensitization inventory, Springerplus 5(1) (2016) 1837.

[55] E.A. Dixon, G. Benham, J.A. Sturgeon, S. Mackey, K.A. Johnson, J. Younger, Development of the Sensory Hypersensitivity Scale (SHS): a self-report tool for assessing sensitivity to sensory stimuli, J. Behav. Med. 39(3) (2016) 537-50.

[56] P. de la Coba, S. Bruehl, C.M. Galvez-Sanchez, G.A. Reyes Del Paso, Slowly Repeated Evoked Pain as a Marker of Central Sensitization in Fibromyalgia: Diagnostic Accuracy and Reliability in Comparison With Temporal Summation of Pain, Psychosom. Med. 80(6) (2018) 573-580.

[57] N. Egloff, N. Klingler, R. von Kanel, R.J. Camara, M. Curatolo, B. Wegmann, E. Marti, M.L. Ferrari, Algometry with a clothes peg compared to an electronic pressure algometer: a randomized cross-sectional study in pain patients, BMC Musculoskelet. Disord. 12 (2011) 174.
