# Supplementary material for: Tests for central sensitization in general practice: a Delphi study
Source: BMC Fam Pract. 2021 Oct 19;22:206. doi: 10.1186/s12875-021-01539-0 (PMC8527602; doi:10.1186/s12875-021-01539-0)
Supplement: Supplementary file 6 — Additional file 6: Appendix 6. List of participants [file 12875_2021_1539_MOESM6_ESM.docx]

**Appendix 6: list of participants**

Tests for central sensitization in general practice: a Delphi **study**

Carine den Boer, MD^1^

Berend Terluin MD, PhD^1^
Johannes C. van der Wouden PhD^1^
Annette H. Blankenstein MD, PhD^1^
Henriëtte E. van der Horst MD, PhD^1^

1. Amsterdam UMC, location VUmc, Department of General Practice, Amsterdam Public Health research institute, the Netherlands.

Correspondence:

C. den Boer

Amsterdam UMC, location VUmc

Department of General Practice

Amsterdam Public Health research institute

Van der Boechorststraat 7

1081 BT Amsterdam

The Netherlands

Telephone: +31613693267

Email: [c.denboer@amsterdamumc.nl](mailto:c.denboer@amsterdamumc.nl)

**Appendix 6: list of participants**

| Profession | Country | Research expertise | MUS expertise | Chronic pain expertise |
| --- | --- | --- | --- | --- |
| General practitioner | Netherlands | x | x |  |
| General practitioner | Netherlands | x | x |  |
| General practitioner | Netherlands | x | x |  |
| General practitioner | Netherlands |  | x |  |
| General practitioner | Netherlands |  | x |  |
| General practitioner | Netherlands |  | x |  |
| General practitioner | Netherlands | x |  |  |
| General practitioner | Netherlands | x |  |  |
| General practitioner | Netherlands | x |  |  |
| General practitioner | Netherlands | x |  | x |
| General practitioner | UK | x | x |  |
| General practitioner | UK | x | x |  |
| Physiotherapist, rehabilitation scientist | Netherlands | x | x |  |
| Physiotherapist, epidemiologist, human movement scientist | Netherlands | x |  | x |
| Physiotherapist | Netherlands |  | x |  |
| Physiotherapist | Netherlands |  | x |  |
| Physiotherapist | Netherlands | x |  | x |
| Physiotherapist, epidemiologist, psychologist | Netherlands | x |  | x |
| Physiotherapist, epidemiologist | Belgium | x |  | x |
| Physiotherapist | Belgium | x |  | x |
| Human movement scientist | Netherlands | x |  |  |
| Gastroenterologist | USA | x | x |  |
| Neurologist | Netherlands | x | x |  |
| Anesthesiologist | Netherlands | x |  | x |
| Professor of psychosomatic medicine | Netherlands | x | x |  |
| Psychologist | New Zealand | x | x |  |
| Psychologist | Netherlands | x | x |  |
